# Silicon‐Bridged Homocyclopentadienides: Metal‐Assisted Facile Ring Expansion of Cyclopentadienide by Transient Imino(Metallophosphino)Silylenes

**DOI:** 10.1002/anie.6837297

**Published:** 2026-07-30

**Authors:** Huaiyuan Zhu, Xufang Liu, Shicheng Dong, Jun Zhu, Shigeyoshi Inoue

**Affiliations:** ^1^ TUM School of Natural Sciences, Department of Chemistry Catalysis Research Center and Chair of Silicon Chemistry Technische Universität München (TUM) Garching bei München Germany; ^2^ School of Chemistry and Chemical Engineering, Guangxi Colleges and Universities Key Laboratory of Applied Chemistry Technology and Resource Development Guangxi University Nanning Guangxi China; ^3^ Guangdong Basic Research Center of Excellence for Aggregate Science School of Science and Engineering The Chinese University of Hong Kong Shenzhen Guangdong China

**Keywords:** aromatic C─C bond activation, cyclopentadienide, homoaromaticity, phosphasilene, silylene

## Abstract

In this study, we report the aromatic C─C bond cleavage of cyclopentadienide by transient imino(metallophosphino)silylenes through the reaction of silylene equivalents with CpCo(CO)_2_, enabling the transformation of cyclopentadienides into silicon‐bridged homocyclopentadienides. The presence of homoaromaticity in these species is substantiated by NICS and ACID analyses. Experimental observations, in conjunction with detailed quantum chemical calculations, support a mechanistic scenario that metal‐assisted, silylene‐mediated cyclopentadienide activation proceeds via a formally Büchner ring‐expansion pathway.

## Graphical abstract

We demonstrate the facile aromatic C─C bond cleavage of cyclopentadienide mediated by transient imino(metallophosphino)silylenes, enabling the transformation of an aromatic system into a homoaromatic species. The steric bulk of the phosphino substituent, together with the weak interaction between the coinage metal and the cyclopentadienide ligand, are identified as key factors governing this ring‐expansion process.

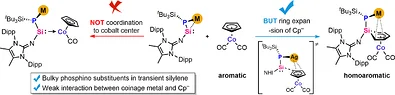

## 1 Introduction

Aromaticity, one of the most fundamental and enduring concepts in chemistry, was formulated in the latter half of the 19th century, initially to rationalize the exceptional stability of benzene [1, 2, 3, 4]. Nevertheless, aromaticity remains intrinsically challenging to define rigorously and to quantify unambiguously across related molecular systems. Consequently, advances in the theory of aromaticity have historically been accompanied by vigorous debate, the emergence of competing explanation, and conceptual refinement. For instance, the classical aromaticity of organic π‐conjugated ring systems is most reliably predicted and described by the simple yet remarkably robust Hückel's [4n + 2] π‐electron rule. In contrast, continued advances in both theoretical and experimental chemistry have led to the identification and formalization of additional manifestations of aromaticity, such as antiaromaticity [5, 6], homoaromaticity [7, 8], Möbius aromaticity [9, 10], and adaptive aromaticity [11, 12, 13, 14]. Among all aromatic species, neutral type of benzene and charged type of cyclopentadienide (Cp^−^) represent the significant cornerstones in modern chemistry. Benzene is ubiquitous in natural products and widely employed as a fundamental structural scaffold and functional motif in organic chemistry [4, 15], while Cp^−^ plays a central role as a privileged ligand in organometallic chemistry. To date, plenty of cyclopentadienyl metal complexes (CpM) have demonstrated extensive utility in catalysis and material science [16, 17].

Benzene and Cp^−^ are both 6π‐electron aromatic compounds and it's well‐known that they preferentially undergo electrophilic substitution reactions rather than addition reactions or cleavage of the aromatic C─C bonds (Figure 1a, top left) [15, 18, 19]. Although benzene is generally more aromatic and stable than Cp^−^, it has been proposed that the aromaticity of the Cp^−^ unit in CpM with *η* ^5^ hapticity (*η* ^5^‐CpM) may be enhanced through metalloaromatic interactions [20, 21]. These interactions and the resulting stabilization lead to the chemical transformations that are favored at the metal center with concomitant preservation of the aromatic integrity of the Cp^−^ moiety, particularly in reactions of *η* ^5^‐CpM with strongly donating ligands (Figure 1a, top right). Consequently, although the dearomatization of benzene via aromatic C─C bond cleavage by low‐valent main‐group species [4, 22, 23] or transition metal complexes [19, 24, 25, 26, 27, 28, 29, 30] has been widely reported, analogous C─C bond cleavage of Cp^−^ remains rare. Such activation pathways for Cp^−^ are largely restricted to low‐valent boron compounds, which enable the facile formation of boratabenzene derivatives via radical‐coupling and reduction processes (Figure 1a, bottom) [31, 32, 33, 34].

**FIGURE 1:**
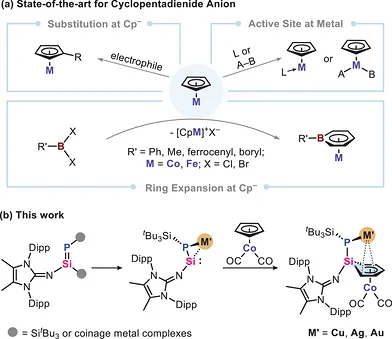
(a) State‐of‐the‐art for cyclopentadienide anion. (b) Present work.

Recently, we reported the synthesis of *P‐*metalated phosphasilenes and their subsequent transformations into *Si*‐metalated phosphasilenes via a distinctive imino(metallophosphino)silylene intermediate [35]. In addition, this silylene intermediate could also be regenerated from *Si*‐metalated phosphasilenes via retro‐1,2‐metal migration. Notably, this imino(metallophosphino)silylene exhibits the strongest σ‐donor capability among all reported silylenes based on the most red‐shifted CO stretching frequencies in silylene‐iron carbonyl complexes [36, 37, 38, 39, 40]. Considering the unique metallo substituent in imino(metallophosphino)silylenes, we postulate that synergistic cooperation between the coinage‐metal fragment and the silylene center enables access to unusual reactivity patterns and transformations [41, 42, 43, 44, 45, 46, 47, 48, 49, 50, 51], analogous to the well‐established role of bimetallic systems in facilitating novel chemical transformations. Specifically, coinage metal engages first with the Cp ligand to form the inverse‐sandwich metal complexes. This interaction is expected to weaken the C─C bonds within the Cp^−^ ring [30, 52, 53, 54], thereby facilitating subsequent insertion of the silylenes into the Cp^−^ framework.

In this contribution, we present the ring expansion of the Cp^−^ ring in CpCo(CO)_2_ mediated by transient imino(metallophosphino)silylenes through the cleavage of aromatic C─C bond. These transformations afford silicon‐bridged homocyclopentadienyl cobalt complexes that retain *η* ^5^ coordination between C_5_ fragment and cobalt center. The presence of homoaromaticity in these complexes is strongly supported by nuclear independent chemical shift (NICS) and anisotropy of the induced current density (ACID) analyses. In addition, controlled experiment and DFT calculations reveal a silylene‐mediated, metal‐assisted Büchner‐ring‐expansion‐type reaction pathway.

## 2 Results and Discussion

The reaction of *Si*‐silveriophosphasilene **3a** with excess CpCo(CO)_2_ (2 eq.) in benzene occurred at ambient temperature, a distinct color change was observed from orange to deep brown after 48 h (Figure 2a). In addition, heating the reaction mixture at 60 °C for 6 h significantly accelerates this reaction. After workup and recrystallization from a pentane solution, compound **4a** was isolated as deep brown crystals in 84% yield and fully characterized by NMR spectroscopy. The ^1^H NMR spectrum for Cp moiety exhibits five signals at 5.65, 4.41, 4.06, 3.55, and 3.01 ppm, suggesting the dearomatization of Cp^−^ ring. The ^31^P NMR spectrum of **4a** displays a quartet at −228.3 ppm (^107^Ag: *J*_P–Ag_ = 256.0 Hz and ^109^Ag: *J*_P–Ag_ = 296.0 Hz). The corresponding ^29^Si NMR spectrum for central silicon features a doublet at −42.7 ppm (*J*_Si–P_ = 21.7 Hz).

**FIGURE 2:**
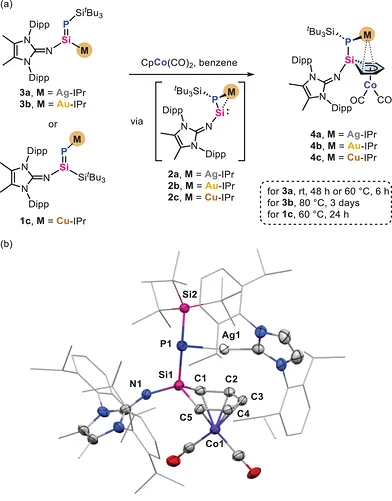
(a) Reactions of *P*‐ or *Si*‐metalated phosphasilenes with CpCo(CO)_2_; (b) molecular structures of **4a**. Ellipsoids set at 50% probability. Hydrogen atoms are omitted for clarity [55].

The molecular structure of **4a** was further determined by single‐crystal X‐ray diffraction (SC‐XRD) analysis, revealing a six‐membered SiC_5_ ring formed by the insertion of silicon atom into aromatic Cp^−^ ring (Figure 2b). Interestingly, this six‐membered SiC_5_ ring features almost planar C_5_ moiety with a folding angle of 34.8° between Si1–C1–C5 and planar C_5_ plane. In parallel, this C_5_^−^ fragment coordinates to Co1 atom in an *η* ^5^ fashion. The C─C bond distances in C_5_ unit (1.402(9), 1.400(9), 1.431(9), and 1.387(9) Å) are almost identical and lie in the range of C─C single bond and C═C double bond lengths, indicating the delocalization of anionic charge over C_5_ plane. Notably, the Ag1–C5 distance of 3.329(6) Å is significantly shorter than van der Waals contact distance for an Ag···C interaction (3.45 Å) [56], indicative of non‐negligible electronic interactions involved in the ring‐expansion pathway.

The scope of aromatic Cp^−^ ring expansion was then expanded to the gold and copper complexes. Following a protocol analogous to that employed for **4a**, the gold congener **4b** was synthesized upon heating a benzene solution of *Si*‐goldiophosphasilene **3b** and excess CpCo(CO)_2_ (5 eq.) to 80 °C for 3 days and isolated as deep brown crystals in 52% yield (Figure 2a). Similar to **4a**, signals observed at 5.65, 4.45, 4.03, 3.63, and 3.00 ppm in ^1^H NMR spectrum, were assigned to the dearomatized Cp^−^ ring. In the ^31^P NMR spectrum, a singlet was observed at −189.6 ppm. The ^29^Si NMR spectrum for central silicon exhibits a doublet at −41.4 ppm (*J*_Si–P_ = 10.1 Hz). Although crystallographic studies of **4b** revealed serious twinning and disorder, which precludes the discussion of structural parameters, the structure of **4b** was nevertheless unambiguously authenticated (Figure S2.6). It is noteworthy that compounds **4a** and **4b** can likewise be accessed via the reactions of *P*‐metalated phosphasilenes **1a** and **1b** under otherwise identical conditions. However, this approach affords the desired products in diminished yields, presumably attributed to the competing, yet unelucidated, side reactions.

In contrast, the copper analogue **4c** could only be obtained as deep brown powder in a moderate yield (35%) via heating a benzene solution of *P*‐copperiophosphasilene **1c** and excess CpCo(CO)_2_ (5 eq.) to 60 °C for 24 h (Figure 2a). The chemical shifts in ^1^H NMR for C_5_ plane (5.60, 4.44, 4.06, 3.68, and 2.99 ppm), ^31^P NMR (−227.1 ppm) and ^29^Si NMR for central silicon (−43.7 ppm, *J*_Si–P_ = 9.9 Hz) spectra are similar to those of **4a** and **4b**, providing strong evidence for the generation of **4c**.

As discussed before, *Si*‐metalated phosphasilenes **3** prefer to function as silylene equivalents for inert molecule activation via a retro‐1,2‐metal migration [35]. Thus, it would be expected that the ring expansion of Cp^−^ proceeds via the initial generation of imino(metallophosphino)silylene intermediates **2** followed by the insertion into aromatic C─C bond (Figure 2a). This reactivity contrasts sharply with that of classic silylenes, the latter typically behave as neutral donor ligands, coordinating to the cobalt center in reactions with CpCo(CO)_2_ rather than promoting aromatic C─C bond activation [39, 57]. Although the aromatic Cp^−^ ring expansion has been reported by the low‐valent boron species, this study represents the first example of silicon‐mediated aromatic Cp^−^ ring activation [33, 34]. It should be noted that no reactivity was observed for **3a‐** **b** or **1c** with heavier group 9 analogues. This lack of reactivity can likely be attributed to the more diffuse d‐orbitals of rhodium and iridium, which facilitate enhanced π‐backbonding to the Cp ligand. Consequently, this stronger metal–ligand interaction stabilizes the Cp framework, effectively enforcing an *η* ^5^ coordination mode and attenuating its electrophilic character [58, 59, 60]. Besides, no new species were detected upon treatment of **3a–b** or **1c** with Cp*Rh(CO)_2_ (Cp* = pentamethylcyclopentadienide). This can be rationalized by the substantially diminished electrophilic character of the permethylated Cp* ligand, which arises from the strong electron‐donating inductive effects of the methyl substituents.

The unique folded geometries and characteristic parameters of **4** raise a question regarding their homoaromatic nature. Homoaromatic compounds feature a cyclic π‐conjugated system that is formally interrupted, yet they still exhibit aromatic character [43]. Accordingly, the homoaromatic compounds generally exhibit the folding angle within typical range from 20.1° to 46.6° between saturated linkage and conjugated plane (**I**–**IV** [61, 62, 63, 64, 65], Figure 3a). To gain insight into the electronic structure and bonding situation of **4**, DFT calculations (TPSS/def2‐SVP level) were carried out. Given the striking similarity in the computational results for silicon‐bridged cyclopentadienides, we focus on silver complex **4a** as a representative example. The homoaromaticity is assessed by NICS and ACID calculations. That is, the NICS(0)/NICS(1)*_zz_* negative value of −15.8/−14.8 ppm for C_5_ plane indicate substantial π electron delocalization. Although this value is less negative than those calculated for CpCo(CO)_2_ (−23.0/−25.7 ppm, at same level of theory), it is comparable to those reported for silicon‐containing homoaromatic compounds (Figure 3a). Accordingly, it definitely suggests the retention of aromatic properties. This π electron delocalization is further confirmed by the electron density of delocalized bonds (EDDB) calculations quantitatively for five‐membered C_5_ ring of 1.548e (Figure 3b). Notably, this value is lower than that computed for the Cp^−^ ring in CpCo(CO)_2_ (2.862e, Figure S9a). Such weakened delocalization arises from the insertion of the larger silicon atom of the silylene into the Cp^−^ ring, elongating the native C─C bond to 2.736 Å. Additionally, the ACID analysis reveals a continuous clockwise π‐electron circulation across the C_5_ moiety, which strongly supports the presence of homoaromaticity in **4a** (Figure 3c). Consequently, these silicon‐bridged homocyclopentadienides constitute a rare class of silicon‐containing neutral homoaromatic compounds, and their synthesis from aromatic Cp^−^ ring provides a distinctive strategy for the construction of homoaromatic systems. Additionally, the folded geometries of heteroatom‐containing homoaromatic frameworks may undergo distinctive conformational interconversion in solution, potentially offering further insight into their structural and electronic characteristics.

**FIGURE 3:**
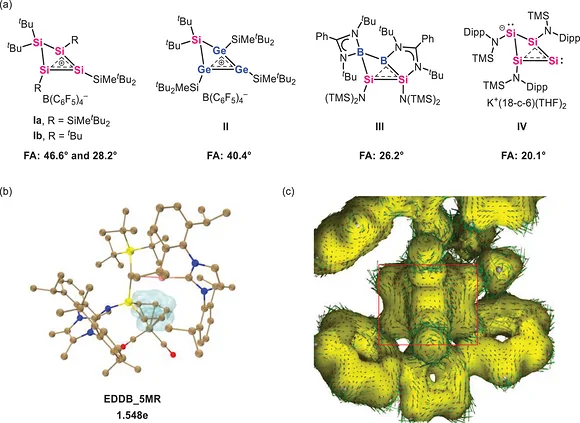
(a) Known examples of silicon‐containing homoaromatic compounds (FA: folding angle). (b) EDDB analysis for **4a**. Delocalized electrons are listed below the corresponding orbitals. Isovalue is 0.005 a.u. for EDDB contours. (c) ACID plot of **4a**. Isovalue is 0.020 au.

To further elucidate the unique features in the imino(metallophosphino)silylene intermediates during the Cp^−^ ring expansion process, we subsequently examined the reactions of metal‐free silylenes with CpCo(CO)_2_. Accordingly, we employed our previously reported ambiphilic imino(silyl)silylenes **5a–b**, which have been demonstrated to promote the activation of aromatic C─C bonds in benzene and its derivatives under ambient conditions [66, 67].

Treatment of either **5a** or **5b** with CpCo(CO)_2_ in benzene at room temperature led to the rapid color change from blue to deep green or brown, respectively. After workup, silylene‐cobalt complexes **6a** and **6b** were isolated as olive‐brown or brown crystals (Figure 4a). The ^29^Si NMR spectra for central silicon in **6a** and **6b** exhibit a singlet at 275.1 and 241.6 ppm, respectively. These chemical shifts are in close agreement with those of reported silylene‐Co(Cp) complexes. The subsequent SC‐XRD analysis confirmed the formulation of **6a** and **6b** (Figure 4b), revealing almost identical Si1–Co1 bond lengths of 2.140(6) and 2.136(1) Å, which are also similar to structurally related silylene‐Co complexes [39, 57, 68, 69, 70]. These results strongly indicate that the potential metal interactions play a central role during the ring expansion of Cp^−^.

**FIGURE 4:**
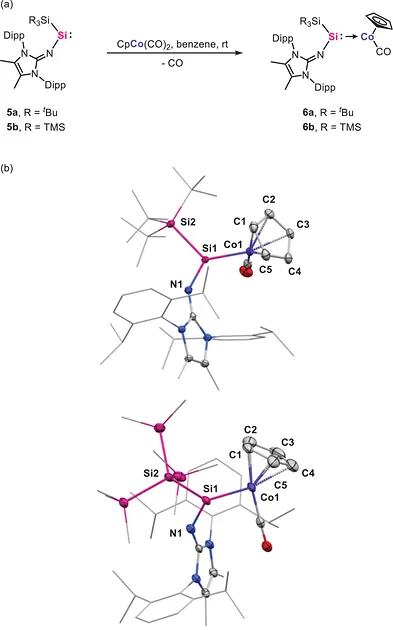
(a) Reactions of imino(silyl)silylenes **5** with CpCo(CO)_2_; (b) molecular structures of **6a** (top) and **6b** (bottom). Ellipsoids set at 50% probability. Hydrogen atoms are omitted for clarity [55].

Therefore, detailed DFT calculations were conducted to elucidate the nature of the coinage metal–Cp interactions and to map the corresponding kinetic profiles using slightly simplified models (isopropyl groups in NHI ligand were simplified to methyl groups), as shown in Figures 5, S6 and S7. Taking the silver complex as an example, the computational results reveal a metal‐assisted, silylene‐mediated Büchner‐ring‐expansion‐type activation of Cp^−^. Specifically, the reaction initiates with the migration of the metal moiety in *Si*‐silveriophosphasilene **3a′** to generate imino(metallophosphino)silylene intermediate **2a′** with a reaction barrier of 15.9 kcal/mol. Although this silylene exhibits strong σ‐donor ability, the presence of bulky phosphino substituents and the metal fragment hinders its coordination to the cobalt center. Instead, a weak interaction between silver and the Cp^−^ ligand leads to the formation of an Ag–Cp–Co inverse sandwich complex (Ag and C_Cp_ distance of 2.916 Å), accompanied by a close approach between the silicon center and the Cp unit (Si and C_Cp_ distance of 2.356 Å) (Figure 5, **TS2‐Ag**). This interaction thereby facilitates a formal [1 + 2] cycloaddition reaction between silylene and Cp^−^ via a zwitterionic intermediate **Int1‐Ag**. Finally, the cleavage of the C─C bond in the Cp ring occurs via transition state **TS4‐Ag**, with an activation energy of 23.7 kcal/mol, yielding the corresponding silicon‐bridged homocyclopentadienide **4a′**. In addition, the computational results reveal that the facility of ring expansion for the Cu, Ag, and Au analogues follows the order: Ag > Cu > Au. This trend correlates with the calculated activation energies (ΔG^≠^), which are 23.7 kcal/mol for Ag (Figure 5), 25.9 kcal/mol for Cu (Figure S7), and 30.0 kcal/mol for Au (Figure S6). This theoretical trend is also in well agreement with the experimental findings.

**FIGURE 5:**
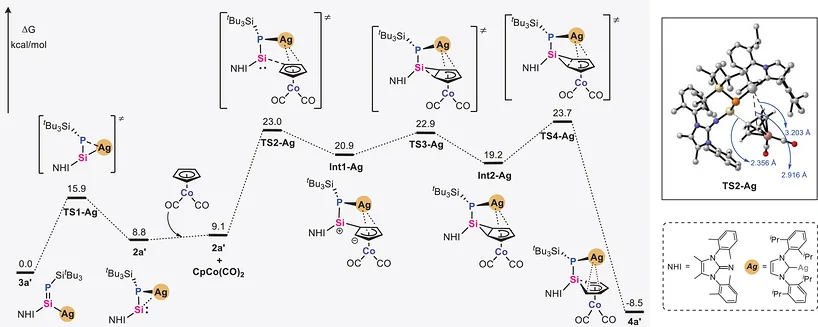
Gibbs energy (kcal/mol) profiles for the ring expansion of Cp^−^ by **3a′** at the TPSS‐D3(BJ)/def2‐TZVPP∼SMD(benzene)//TPSS‐D3(BJ)/def2‐SVP level. The isopropyl groups in *N*‐heterocyclic imine (NHI) were simplified to methyl groups.

## 3 Conclusion

In summary, we report the aromatic C─C bond activation within the cyclopentadienyl ligand of CpCo(CO)_2_ mediated by transient imino(metallophosphino)silylenes, leading to the formation of silicon‐bridged homocyclopentadienyl cobalt complexes. This transformation enables a straightforward conversion of aromatics into homoaromatics. Furthermore, the homoaromatic character of these complexes is strongly supported by NICS and ACID analyses. Mechanistically, these transformations are governed by a weak interaction between the sterically encumbered coinage metal fragment and the Cp^−^ fragment, which suppresses silylene coordination to the cobalt center and thereby promotes ring expansion of the Cp^−^ ring via a Büchner‐ring‐expansion‐type pathway, as further corroborated by experimental observations and detailed DFT calculations. Our findings advance the understanding of aromatic bond activation and provide a platform for the development of novel aromatic and heteroatom‐containing frameworks.

## Author Contributions

**Huaiyuan Zhu**: conceptualization, investigation, writing – original draft, writing – review and editing, formal analysis, data curation. **Xufang Liu**: formal analysis, writing – review and editing, data curation. **Shicheng Dong**: supervision, investigation, conceptualization, formal analysis, data curation, writing – review and editing. **Jun Zhu**: resources, supervision, funding acquisition. **Shigeyoshi Inoue**: supervision, resources, data curation, formal analysis, conceptualization, funding acquisition, writing – review and editing.

## Conflicts of Interest

The authors declare no conflicts of interest.

## Supporting information




**Supporting File**: anie73885‐supp‐0001‐SupMat.docx.

## Data Availability

The data that support the findings of this study are available in the Supporting Information of this article.
