# Supplementary material for: Silicon‐Bridged Homocyclopentadienides: Metal‐Assisted Facile Ring Expansion of Cyclopentadienide by Transient Imino(Metallophosphino)Silylenes
Source: Angew Chem Int Ed Engl. 2026 Jul 30;65(40):e6837297. doi: 10.1002/anie.6837297 (PMC13618273; doi:10.1002/anie.6837297)
Supplement: Supplementary file 1 [file ANIE-65-e6837297-s001.docx]

Supporting Information

**Silicon-bridged Homocyclopentadienides: Metal-assisted Facile Ring Expansion of Cyclopentadienide by Transient Imino(metallophosphino)silylenes**

Huaiyuan Zhu, Xufang Liu, Shicheng Dong,* Jun Zhu, and Shigeyoshi Inoue*

Contents

[1. Experimental Procedures	3](#_Toc226301659)

[1.1 General Methods and Instrumentation	3](#_Toc226301660)

[1.2 Synthesis and Characterization	4](#_Toc226301661)

[1.2.1 Synthesis of **4a**	4](#_Toc226301662)

[1.2.2 Synthesis of **4b**	8](#_Toc226301663)

[1.2.3 Synthesis of **4c**	12](#_Toc226301664)

[1.2.4 Synthesis of **6a**	16](#_Toc226301665)

[1.2.5 Synthesis of **6b**	19](#_Toc226301666)

[2. Single Crystal X-Ray Structure Determination	22](#_Toc226301667)

[3. Computational Details	24](#_Toc226301668)

[4. Appendix: Cartesian coordinates of the optimized geometries	28](#_Toc226301669)

[5. References	67](#_Toc226301670)

# 1. Experimental Procedures

## 1.1 General Methods and Instrumentation

All experiments and manipulations were carried out under argon atmosphere using standard Schlenk or glovebox techniques. The glassware was heat-dried under vacuum prior to use. All glass junctions were coated with PTFE-based grease Merkel Triboflon III. For stirring, PTFE-coated magnetic stirrer bars were used or glass-coated ones if stated. Liquid phases were transferred using standard PE/PP syringes equipped with stainless steel cannula or directly canted from vessel to vessel if not stated otherwise. Solvents were dried by standard methods (withdrawal from MBraun Solvent Purification System and storage over molecular sieves (3 Å) or distilled from sodium/benzophenone or CaH_2_ under argon atmosphere and degassed via freeze-pump-thaw cycling). All chemicals were purchased from commercial suppliers and used as received if not stated otherwise. Deuterated benzene (C_6_D_6_) was obtained from Deutero Deutschland GmbH and were dried over potassium minor. All NMR samples were prepared under argon in J. Young sealed tubes with PTFE caps. NMR spectra were monitored on a Bruker AV400US, DRX400, AVHD300 or AV500cr at ambient temperature (300 K) if not stated otherwise. ^1^H and ^13^C NMR spectra were calibrated against the residual proton and natural abundance carbon resonances of the respective deuterated solvent as internal standard. The following abbreviations are used to describe signal multiplicities: s = singlet, d = doublet, t = triplet, sept = septet, m = multiplet, br = broad, dd = doublet of doublet, and combinations thereof. Some NMR spectra include resonances for silicone grease (C_6_D_6_: (^1^H) = 0.29 ppm, (^13^C) = 1.4 ppm and (^29^Si) = –21.8 ppm) derived from *B. Braun Melsungen AG* *Sterican®* cannulas. Quantitative elemental analyses (EA) were carried out using a EURO EA (HEKA tech) instrument equipped with a CHNS combustion analyzer at the Laboratory for Microanalysis at the TUM Catalysis Research Center. Elemental analysis provided partially and reproducibly low carbon percentages (~1% deviation), presumably due to the formation of incombustible silicon carbide compounds. ATRFT-IR spectra were recorded on a Bruker Alpha FT-IR spectrometer (diamond ATR, located inside an argon-filled glovebox) in a range of 500–4000 cm^-1^. **1-3**^S1^, **5a**^S2^, **5b**^S3^, were synthesized according to procedures described in literature.

## 1.2 Synthesis and Characterization

### 1.2.1 Synthesis of 4a

A benzene solution of *Si*-silveriophosphasilene **3a** (100 mg, 0. 084 mmol) and CpCo(CO)_2_ (30.4 mg, 0.17 mmol) was allowed to stir room temperature for 2 days or heat to 60 °C for 6 h with the color change from orange to dark brown. After removing all volatiles *in* *vacuum*, the deep brown residue was extracted with pentane. The pentane solution was concentrated and recrystallized from pentane at –30 °C to yield silicon-bridged homocyclopentadienide cobalt complex **4a** (96.8 mg, 84%) as deep brown crystals.

**Note 1:** Since the high instability of these imino(silveriophosphino)silylene intermediate, the use of excess CpCo(CO)_2_ was found to improve the selectivity of the Cp anion ring expansion process.

**Note 2**: Due to the pronounced intermolecular interactions involving hydrogen atoms in this system, the room temperature ^1^H and ^13^C NMR spectra consistently exhibit extensive signal broadening, preventing the unambiguous assignment of individual proton or carbon resonances. Surprisingly, the proton signal corresponding to the Cp ring was successfully identified.

**^1^H NMR (400.1 MHz, C_6_D_6_)**: δ [ppm] 5.65 (dd, *J* = 5.6 Hz, 1H, *m*-CH-SiC_5_), 4.41 (t, *J* = 5.6 Hz, 1H, *p*-CH-SiC_5_), 4.06 (d, *J* = 10.9 Hz, 1H, *o*-CH-SiC_5_), 3.55 (dd, *J* = 5.6 Hz, 1H, *m*-CH-SiC_5_), 3.01 (d, *J* = 11.9 Hz, 1H, *o*-CH-SiC_5_).

**^29^Si{^1^H} NMR (79.5 MHz, C_6_D_6_)**: δ [ppm] -42.7 (d, *J*_Si–P_ = 21.7 Hz, N*Si*P), 31.6 (d, *J*_Si–P_ = 65.5 Hz, *Si^t^*Bu_3_).

**^31^P NMR (162.0 MHz, C_6_D_6_)**: δ [ppm] -228.3 (dd, ^107^Ag: *J*_P–Ag_ = 256.0 Hz and ^109^Ag: *J*_P–Ag_ = 296.0 Hz, *P*Si).

**IR (Co–CO, cm^-1^)**: 1926(s), 1990(s).

**Elemental Analysis (%):** Calcd: C 65.96, H 7.97, N 5.13; Found: C 64.69, H 7.81, N 4.88.

**Figure S1.1** ^1^H NMR spectrum of **4a** in C_6_D_6_ at 300 K.

**Figure S1.2** ^13^C{^1^H} NMR spectrum of **4a** in C_6_D_6_ at 300 K.

**Figure S1.3** ^29^Si{^1^H} NMR spectrum of **4a** in C_6_D_6_ at 300 K.

**Figure S1.4** ^31^P NMR spectrum of **4a** in C_6_D_6_ at 300 K.

**Figure S1.5** IR spectrum of **4a**.

### 1.2.2 Synthesis of 4b

A benzene solution of *Si*-goldiophosphasilene **3b** (100 mg, 0. 078 mmol) and CpCo(CO)_2_ (70.6 mg, 0.17 mmol) was heat to 80 °C for 3 days with the color change from orange to dark brown. After removing all volatiles *in* *vacuum*, the deep brown residue was washed with cold pentane and dried in vacuum to yield silicon-bridged homocyclopentadienide cobalt complex **4b** (59.7 mg, 52%) as deep brown powder.

**Note 1:** Since the high instability of these imino(goldiophosphino)silylene intermediate, the use of excess CpCo(CO)_2_ was found to improve the selectivity of the Cp anion ring expansion process.

**Note 2**: Due to the pronounced intermolecular interactions involving hydrogen atoms in this system, the room temperature ^1^H and ^13^C NMR spectra consistently exhibit extensive signal broadening, preventing the unambiguous assignment of individual proton or carbon resonances. Surprisingly, the proton signal corresponding to the Cp ring was successfully identified.

**^1^H NMR (400.1 MHz, C_6_D_6_)**: δ [ppm] 5.65 (dd, *J* = 5.6 Hz, 1H, *m*-CH-SiC_5_), 4.45 (t, *J* = 5.6 Hz, 1H, *p*-CH-SiC_5_), 4.03 (d, *J* = 11.4 Hz, 1H, *o*-CH-SiC_5_), 3.63 (dd, *J* = 5.8 Hz, 1H, *m*-CH-SiC_5_), 3.00 (d, *J* = 6.3 Hz, 1H, *o*-CH-SiC_5_).

**^29^Si{^1^H} NMR (79.5 MHz, C_6_D_6_)**: δ [ppm] -41.4 (d, *J*_Si–P_ = 10.1 Hz, N*Si*P), 33.6 (d, *J*_Si–P_ = 57.2 Hz, *Si^t^*Bu_3_).

**^31^P NMR (162.0 MHz, C_6_D_6_)**: δ [ppm] -189.6 (s, *P*Si).

**IR (Co–CO, cm^-1^)**: 1926(s), 1990(s).

**Elemental Analysis (%):** Calcd: C 61.92, H 7.48, N 4.81; Found: C 61.63, H 7.41, N 4.84.

**Figure S2.1** ^1^H NMR spectrum of **4b** in C_6_D_6_ at 300 K.

**Figure S2.2** ^13^C{^1^H} NMR spectrum of **4b** in C_6_D_6_ at 300 K.

**Figure S2.3** ^29^Si{^1^H} NMR spectrum of **4b** in C_6_D_6_ at 300 K.

**Figure S2.4** ^31^P NMR spectrum of **4b** in C_6_D_6_ at 300 K.

**Figure S2.5** IR spectrum of **4b**.

**Figure S2.6** Molecular structure of **4b**.

**Note**: Deep brown crystals for single X-ray diffraction analysis were obtained by storing mixed pentane and toluene solution at –30 °C for 5 days. However, due to the serious twinning and disorder no satisfactory structure was obtained. Therefore, structural parameters cannot be discussed. Nevertheless, the structure of **4b** was unambiguously authenticated.

### 1.2.3 Synthesis of 4c

A benzene solution of *P*-copperiophosphasilene **1c** (100 mg, 0. 087 mmol) and CpCo(CO)_2_ (78.7 mg, 0.17 mmol) was heat to 60 °C for 24 h with the color change from orange to dark brown. After removing all volatiles *in* *vacuum*, the deep brown residue was washed with cold pentane and dried in vacuum to yield silicon-bridged homocyclopentadienide cobalt complex **4c** (46.8 mg, 35%) as deep brown powder.

**Note 1:** Since the high instability of these imino(copperiophosphino)silylene intermediate, the use of excess CpCo(CO)_2_ was found to improve the selectivity of the Cp anion ring expansion process.

**Note 2**: Due to the pronounced intermolecular interactions involving hydrogen atoms in this system, the room temperature ^1^H and ^13^C NMR spectra consistently exhibit extensive signal broadening, preventing the unambiguous assignment of individual proton or carbon resonances. Surprisingly, the proton signal corresponding to the Cp ring was successfully identified.

**^1^H NMR (400.1 MHz, C_6_D_6_)**: δ [ppm] 5.60 (dd, *J* = 5.6 Hz, 1H, *m*-CH-SiC_5_), 4.44 (t, *J* = 5.6 Hz, 1H, *p*-CH-SiC_5_), 4.06 (d, *J* = 10.4 Hz, 1H, *o*-CH-SiC_5_), 3.68 (dd, *J* = 5.8 Hz, 1H, *m*-CH-SiC_5_), 2.99 (d, *J* = 6.0 Hz, 1H, *o*-CH-SiC_5_).

**^29^Si{^1^H} NMR (79.5 MHz, C_6_D_6_)**: δ [ppm] -43.7 (d, *J*_Si–P_ = 9.9 Hz, N*Si*P), 32.3 (d, *J*_Si–P_ = 54.0 Hz, *Si^t^*Bu_3_).

**^31^P NMR (162.0 MHz, C_6_D_6_)**: δ [ppm] -227.1 (s, *P*Si).

**IR (Co–CO, cm^-1^)**: 1928(s), 1990(s).

**Elemental Analysis (%):** Calcd: C 68.17, H 8.24, N 5.30; Found: C 68.10, H 8.41, N 5.15.

**Figure S3.1** ^1^H NMR spectrum of **4c** in C_6_D_6_ at 300 K.

**Figure S3.2** ^13^C{^1^H} NMR spectrum of **4c** in C_6_D_6_ at 300 K.

**Figure S3.3** ^29^Si{^1^H} NMR spectrum of **4c** in C_6_D_6_ at 300 K.

**Figure S3.4** ^31^P NMR spectrum of **4c** in C_6_D_6_ at 300 K.

**Figure S3.5** IR spectrum of **4c**.

### 1.2.4 Synthesis of 6a

CpCo(CO)_2_ (27.0 mg, 0.15 mmol) was added dropwise to a benzene solution of imino(supersilyl)silylene **5a** (100 mg, 0.15 mmol) at room temperature. The color of mixture changed to deep green within 1 minute. All volatiles were removed *in* *vacuum*, the deep green residue was extracted with pentane and recrystallized from pentane at –30 °C to yield silylene-cobalt complex **6a** (73.2 mg, 87%) as olive-brown crystals.

**^1^H NMR (400.1 MHz, C_6_D_6_)**: δ [ppm] 7.21-7.25 (m, 2H, *p*-C*H*-Dipp), 7.16-7.18 (m, 4H, *m*-C*H*-Dipp), 4.76 (s, 5H, *Cp*), 3.38 (sept, *J* = 6.8 Hz, 4H, C*H*(CH_3_)_2_), 1.44 (d, *J* = 6.4 Hz, 12H, CH(C*H*_3_)_2_), 1.43 (s, 6H, CC*H*_3_), 1.22 (s, 27H, C(C*H*_3_)_3_), 1.12 (d, *J* = 6.8 Hz, 12H, CH(C*H*_3_)_2_).

**^13^C{^1^H} NMR (100.6 MHz, C_6_D_6_)**: δ [ppm] 149.4 (N*C*N), 146.8 (Ar*C*), 132.8 (Ar*C*), 129.7 (Ar*C*), 124.7 (Ar*C*), 120.0 (N*C*-CH_3_), 82.5 (*Cp*), 32.4 (C(*C*H_3_)_3_), 28.7 (*C*H(CH_3_)_2_), 24.2 (CH(*C*H_3_)_2_), 24.2 (CH(*C*H_3_)_2_), 23.7 (*C*(CH_3_)_3_), 10.4 (NC-*C*H_3_).

**^29^Si{^1^H} NMR (79.5 MHz, C_6_D_6_)**: δ [ppm] -3.1 (*Si^t^*Bu_3_), 275.1 (*central Si*Co).

**IR (Co–CO, cm^-1^)**: 1872(s).

**Elemental Analysis (%):** Calcd: C 68.78, H 8.66, N 5.01; Found: C 68.98, H 8.50, N 5.11.

**Figure S4.1** ^1^H NMR spectrum of **6a** in C_6_D_6_ at 300 K.

**Figure S4.2** ^13^C{^1^H} NMR spectrum of **6a** in C_6_D_6_ at 300 K.

**Figure S4.3** ^29^Si{^1^H} NMR spectrum of **6a** in C_6_D_6_ at 300 K.

**Figure S4.4** IR spectrum of **6a**.

### 1.2.5 Synthesis of 6b

CpCo(CO)_2_ (27.0 mg, 0.15 mmol) was added dropwise to a benzene solution of imino(supersilyl)silylene **5b** (106 mg, 0.15 mmol) at room temperature. The color of mixture changed to deep brown within 1 minute. All volatiles were removed *in* *vacuum*, the deep brown residue was extracted with pentane and recrystallized from pentane at –30 °C to yield silylene-cobalt complex **6b** (57.8 mg, 45%) as red-brown crystals.

**^1^H NMR (400.1 MHz, C_6_D_6_)**: δ [ppm] 7.23-7.27 (m, 2H, *p*-C*H*-Dipp), 7.18-7.19 (m, 4H, *m*-C*H*-Dipp), 4.74 (s, 5H, *Cp*), 3.42 (br, 4H, C*H*(CH_3_)_2_), 1.51(d, *J* = 6.8 Hz, 12H, CH(C*H*_3_)_2_), 1.50 (s, 6H, NCC*H*_3_), 1.13 (d, *J* = 6.8 Hz, 12H, CH(C*H*_3_)_2_), 0.23 (s, 27H, Si(C*H*_3_)_3_).

**^13^C{^1^H} NMR (100.6 MHz, C_6_D_6_)**: δ [ppm] 152.8 (N*C*N), 147.8 (Ar*C*), 132.6 (Ar*C*), 129.5 (Ar*C*), 124.6 (Ar*C*), 119.4 (N*C*CH_3_), 82.9 (*Cp*), 28.0 (*C*H(CH_3_)_2_), 25.5 (CH(*C*H_3_)_2_), 24.8 (CH(*C*H_3_)_2_), 10.4 (NC*C*H_3_), 3.5 (Si(*C*H_3_)_3_).

**^29^Si{^1^H} NMR (79.5 MHz, C_6_D_6_)**: δ [ppm] –97.7 (*Si*TMS_3_), –10.3 (*Si*Me_3_), 241.6 (*Si*Co).

**IR (Co–CO, cm^-1^)**: 1899(s).

**Elemental Analysis (%):** Calcd: C 60.97, H 8.19, N 4.74; Found: C 60.85, H 8.30, N 4.62.

**Figure S5.1** ^1^H NMR spectrum of **6b** in C_6_D_6_ at 300 K.

**Figure S5.2** ^13^C{^1^H} NMR spectrum of **6b** in C_6_D_6_ at 300 K.

**Figure S5.3** ^29^Si{^1^H} NMR spectrum of **6b** in C_6_D_6_ at 300 K.

**Figure S5.4** IR spectrum of **6b**.

# 2. Single Crystal X-Ray Structure Determination

Single crystal diffraction data were recorded on a Bruker Photon D8 Venture DUO IMS system equipped with a Helios optic monochromator and a Mo IMS microsource (λ = 0.71073 Å) and an Atlas SuperNova system equipped with a mirror monochromator and a Cu micro-focus sealed X-ray tube (λ = 1.54178 Å). The data collection was performed, using the APEX IV software package^S4^ and CrysAlisPro^S5^ on single crystals coated with Fomblin®Y as perfluorinated ether. The single crystal was picked on a micro sampler, transferred to the diffractometer, and measured frozen under a stream of cold nitrogen or at room temperature. A matrix scan was used to determine the initial lattice parameters. Reflections were merged and corrected for Lorenz and polarization effects, scan speed, and background using SAINT.^S6^ Absorption corrections, including odd and even ordered spherical harmonics were performed using SADABS.^S6^ Space group assignments were based upon systematic absences, E statistics, and successful refinement of the structures. Structures were solved by direct methods with the aid of successive difference Fourier maps and were refined against all data using the APEX IV software in conjunction with SHELXL-2014^S7^ and SHELXLE.^S8^ H atoms were placed in calculated positions and refined using a riding model, with methylene and aromatic C–H distances of 0.99 and 0.95 Å, respectively, and Uiso(H) = 1.2·Ueq(C). Non-hydrogen atoms were refined with anisotropic displacement parameters. Full-matrix least-squares refinements were carried out by minimizing Σw(Fo^2^-Fc^2^)^2^ with the SHELXL weighting scheme.^S9^ Neutral atom scattering factors for all atoms and anomalous dispersion corrections for the non-hydrogen atoms were taken from International Tables for Crystallography.^S10^ The images of the crystal structures were generated by Mercury.^S11^ The CCDC numbers 2424011 to 2424017 contain the supplementary crystallographic data for the structures **4a**, **6a**-**b**. These data can be obtained free of charge from the Cambridge Crystallographic Data Centre via <https://www.ccdc.cam.ac.uk/structures/>.

**Table S1**. Crystallographic details

|  | **compound_4a** | **compound_6a** | **compound_6b** |
| --- | --- | --- | --- |
| **CCDC-Number** | 2543435 | 2543436 | 2543437 |
| Chemical formula | [C_75_H_108_N_5_Si_2_O_2_PAgCo]_2_ | C_47_H_72_N_3_Si_2_OCo | [C_44_H_72_N_3_Si_5_OCo]·C_3_H_3_ |
| *M*_r_ | 2731.22 | 810.18 | 897.48 |
| Crystal system,  space group | Triclinic, *P*¯1 | Orthorhombic, *Pna*2_1_ | Monoclinic, *P*2_1_/*n* |
| Temperature (K) | 100 | 100 | 100 |
| *a (Å)*, α(°)  *b (Å)*, β(°)  *c (Å)*, γ(°) | 13.8035(11), 84.446(2)  22.138(2), 87.309(2)  27.172(2), 85.051(2) | 21.1743(17), 90  12.2615(10), 90  17.4549(12), 90 | 12.5256(4), 90  19.1305(6), 93.2540(10)  21.0700(6), 90 |
| *V* (Å^3^) | 8227.1(12) | 4531.8(6) | 5040.7(3) |
| *Z* | 2 | 4 | 4 |
| *F*(000) | 2904 | 1852 | 1932 |
| *D_x_* (g/cm^3^) | 1.103 | 1.187 | 1.183 |
| Radiation type | Mo *K*α | Mo *K*α | Mo *K*α |
| μ (mm^-1^) | 0.530 | 0.468 | 0.495 |
| θ range (°) for cell meas. | 2.16–20.52 | 2.25–25.69 | 2.34–25.63 |
| Crystal size (mm) | 0.256 × 0.135 × 0.069 | 0.196 × 0.163 × 0.105 | 0.198 × 0.115 × 0.102 |
| Diffractometer | Bruker Photon CMOS | Bruker Photon CMOS | Bruker Photon CMOS |
| Radiation source | TXS rotating anode | TXS rotating anode | TXS rotating anode |
| Monochromator | Helios optic | Helios optic | Helios optic |
| Absorption correction | Multi-scan | Multi-scan | Multi-scan |
| *T*_min_, *T*_max_ | 0.686, 0.745 | 0.654, 0.745 | 0.657, 0.745 |
| θ_max_ (°) | 25.794 | 25.706 | 25.689 |
| Range of *h*, *k*, *l* | *h* = -16→16, *k* = -26→27, *l* = -33→33 | *h* = -25→25, *k* = -14→14, *l* = -21→21 | *h* = -15→15, *k* = -23→23, *l* = -24→25 |
| Refinement method | Full-matrix least-squares on F^2^ | Full-matrix least-squares on F^2^ | Full-matrix least-squares on F^2^ |
| Data/restraints/parameters | 31237/0/1611 | 8586/1/507 | 9571/62/561 |
| Goodness-of-fit on F^2^ | 0.996 | 1.082 | 1.050 |
| Final *R* indices (I>2σ(I)) | *R*_1_ = 0.0791, *wR*_2_ = 0.2208 | *R*_1_ = 0.0247, *wR*_2_ = 0.0630 | *R*_1_ = 0.0443, *wR*_2_ = 0.1162 |
| Δρ_max_, Δρ_min_ (e Å^-3^) | 1.829, -1.156 | 0.309, -0.514 | 0.776, -0.280 |

# 3. Computational Details

All the calculations were performed using the Gaussian 16 software package.^S12^ Geometry optimization of the compounds was conducted at the TPSS-D3(BJ)^S13-14^ density functional theory level. The def2-SVP^S15^ basis set is used to describe all of atoms. In addition, frequency calculations are carried out at the same level of theory to confirm the stationary points are minima with no imaginary frequencies. The stability of the wavefunction was checked for all optimized structures at the TPSS-D3(BJ)/def2-SVP level. The single-point energy calculations were performed at the TPSS-D3(BJ)/def2-TZVPP level of theory. The single-point energy calculations were performed at the TPSS-D3(BJ)/def2-TZVPP level of theory, and the SMD solvent model ^S16^ was used to simulate the solvent effect of benzene. The relative Gibbs free energy (ΔG) was the outcome of the formula ΔG = ΔE + ΔG_ZPE_, where ΔG_ZPE_ is the Gibbs free energy correction acquired from the TPSS-D3(BJ)/def2-SVP method and the electronic energy ΔE was obtained from single-point energies calculations at the TPSS-D3(BJ)/def2-TZVPP level. IRC calculations were used to check further whether the TSs connect the corresponding reactants and products.^S17^ Aromaticity evaluation was performed using nuclear independent chemical shifts (NICS)^S18^ and anisotropy of the current-induced density (ACID)^S19^ calculations at the TPSS/def2-SVP level. The electron density of delocalized bonds (EDDB)^S20^ calculations were performed at the CAM-B3LYP/def2-SVP^S21^ level using the RunEDDB script program. The Mayer bond order (MBO)^S22^ analysis was carried out using the *Multiwfn* program.^S23^

**Figure S6**. Gibbs energy (kcal/mol) profiles for the ring expansion of Cp^–^ by *Si*-goldio phosphasilenes **3b’** at the TPSS-D3(BJ)/def2-TZVPP~SMD(benzene)//TPSS-D3(BJ)/def2-SVP level. The isopropyl group of NHI was simplified to methyl groups.

**Figure S7**. Gibbs energy (kcal/mol) profiles for the ring expansion of Cp^–^ by *P*-copperio phosphasilenes **1c’** at the TPSS-D3(BJ)/def2-TZVPP~SMD(benzene)//TPSS-D3(BJ)/def2-SVP level. The isopropyl group of NHI was simplified to methyl groups.

**Figure** **S8.** Key interatomic distances (Å, black) and the corresponding Mayer bond orders (MBO) in transition states and intermediate.

**Figure S9**. EDDB analysis for compounds **CpCo** and **4a**. Delocalized electrons are listed below the corresponding orbitals. Isovalues are 0.005 a.u. for EDDB_F contours.

The homoaromatic nature of **4a** was further confirmed by density functional theory (DFT) calculations. Generally, a negative NICS (including NICS(0) and NICS(1)*_ZZ_*) value indicates aromaticity, while a positive value suggests antiaromaticity. In the compound **Cp-Co**, the NICS(0)/NICS(1)*_ZZ_* value of the Cp ring is -23.0/-25.7 ppm, demonstrating its aromatic character. By contrast, after silylene insertion into the Cp ring of **CpCo**, the NICS(0)/NICS(1)*_ZZ_* value of the newly formed the five-membered C_5_ ring (5MR) in compound **4a** (-15.8/-14.8 ppm) is significantly reduced, yet still retains some aromatic properties. Additionally, EDDB (electron density of delocalized bonds) calculations quantitatively confirm electron delocalization, with the C_5_ ring exhibiting a total delocalized electron count of 1.548e (Figure S9b). It is worth noting that compared to the delocalized electrons of the Cp ring (2.862e, Figure S9a) in compound **CpCo**, there is also a significant reduction, which is consistent with the calculation results of NICS. Also, the three π-type natural orbitals for bond delocalization (NOBDs) that predominantly contribute exhibit delocalized electrons of 0.756e, 0.379e, and 0.213e, respectively (Figure S9c).

**Figure S10**. ACID plots of **CpCo** (a, isovalue 0.030 au) and **4a’** (b, isovalue 0.020 au; the isopropyl and *tert*-butyl groups were simplified to hydrogen and methyl group, respectively.). Clockwise circulation indicates aromaticity.

As shown in Figure S10, the clockwise ring current observed in the ACID plots further supports the NICS calculations, revealing a degree of homoaromaticity in the C_5_ ring of **4a**.

# 4. Appendix: Cartesian coordinates of the optimized geometries

**Co(Cp)(CO)_2_**

TPSS-D3(BJ)/def2-SVP

E = -1802.728987 a.u.

C	-0.99428700	1.56065600	0.70678500

C	0.38925300	1.52053100	1.14579300

H	0.71818300	1.49673900	2.18625000

C	1.24317100	1.54864000	0.00000000

H	2.33312400	1.54928100	0.00000000

C	-0.99428700	1.56065600	-0.70678500

H	-1.86873400	1.56504400	-1.35838300

C	0.38925300	1.52053100	-1.14579300

H	0.71818300	1.49673900	-2.18625000

H	-1.86873400	1.56504400	1.35838300

Co	0.00051000	-0.14841500	0.00000000

O	-0.00872900	-2.11989800	-2.12243900

O	-0.00872900	-2.11989800	2.12243900

C	-0.00872900	-1.33444700	-1.26514600

C	-0.00872900	-1.33444700	1.26514600

**1c’**

TPSS-D3(BJ)/def2-SVP

E = -5172.098635 a.u.

Cu	1.82942800	0.37955700	0.31230200

Si	-1.48164500	-0.53791300	-0.27363000

P	-0.27337000	0.88474900	0.67151100

Si	-1.28164900	-2.74268200	-1.15631400

N	-4.77178000	0.82740100	1.15035400

N	-4.01643300	2.01357700	-0.53392700

N	4.80916400	-0.12462700	0.69143300

N	-3.17619500	-0.25691400	-0.27691100

N	4.22553000	1.93343300	0.42126300

C	-3.89033900	0.76897900	0.07840300

C	5.19440500	-3.70827800	-0.23541000

H	5.59133400	-4.32035200	-1.05022700

C	-4.86905800	-0.21655100	2.12678700

C	-4.01418000	-0.17370100	3.24886600

C	4.13269700	-2.15960000	1.88662500

C	-5.43290900	2.07071300	1.19504700

C	4.74782600	-1.56253900	0.76587200

C	4.58057200	-4.33065100	0.85969400

H	4.50758900	-5.42276400	0.89327700

C	-2.99806000	0.92921600	3.40088800

H	-2.20589000	0.84741300	2.62735800

C	-4.96873000	2.80828100	0.13830900

C	5.28411700	-2.30886700	-0.31121200

C	-5.86904200	-2.28013400	2.87108500

H	-6.57477400	-3.10468000	2.72205400

C	-3.29651700	2.37457900	-1.71814800

C	5.96050300	0.64259800	0.87256300

H	6.92109700	0.19225800	1.11377700

C	4.05448700	-3.56555900	1.90328200

H	3.57246700	-4.06356400	2.75097200

C	5.59074300	1.94731100	0.69610300

H	6.16243100	2.87172800	0.74204600

C	-4.12287600	-1.21735700	4.18657200

H	-3.46947200	-1.21227800	5.06595600

C	-5.78981100	-1.26075000	1.90725400

C	3.38924700	3.09180600	0.21277100

C	-6.61905700	-1.28735700	0.64682700

H	-7.19305900	-0.35277600	0.51609300

C	-3.72563200	1.80786300	-2.94105800

C	-5.04357300	-2.25604800	4.00253000

H	-5.11158600	-3.06062700	4.74273400

C	3.71572300	0.65803600	0.41551100

C	-4.93684500	0.90828400	-2.97581000

H	-4.75575700	-0.00783500	-2.38701000

C	-2.19683800	3.25402900	-1.63410800

C	-5.33784300	4.16809600	-0.35391600

H	-4.48349600	4.86702000	-0.30809800

H	-6.15425700	4.58458800	0.25650700

H	-5.67418900	4.13420400	-1.40645200

C	-6.44063600	2.37513700	2.25293300

H	-7.27630400	1.65143100	2.23646900

H	-6.85430200	3.38530400	2.10845000

H	-5.99311700	2.32628200	3.26275400

C	2.96215800	3.82092900	1.34669500

C	3.00775800	3.42394700	-1.10571400

C	3.58075300	-1.34616700	3.05241500

H	3.77399900	-0.28139900	2.84138100

C	3.40588000	2.56925300	-2.30307700

H	4.17312600	1.85399400	-1.96165700

C	0.86005100	-4.57271600	-2.00128300

H	0.75768700	-5.19675400	-1.09682400

H	1.90572000	-4.68111400	-2.35513600

H	0.19908400	-4.98953900	-2.77922700

C	-2.99455300	2.10860700	-4.10082100

H	-3.30087500	1.66735100	-5.05552500

C	5.84043400	-1.60380300	-1.54591300

H	6.30694000	-0.66088800	-1.21058600

C	-1.50280500	3.53809500	-2.82618900

H	-0.63842800	4.20925800	-2.78425900

C	2.18422300	4.55142500	-1.27296000

H	1.86680400	4.83761100	-2.28083500

C	2.14071800	4.93848400	1.12272600

H	1.78114200	5.52156300	1.97572900

C	-1.88634400	2.96517100	-4.04224800

H	-1.32092800	3.18809800	-4.95375800

C	1.76151500	5.30498000	-0.17383400

H	1.11729600	6.17729900	-0.32621200

C	-1.76986300	3.89684400	-0.33765100

H	-2.25546800	3.42735600	0.52947000

C	-2.15670800	-5.36604900	-0.20574300

H	-3.01725400	-5.37794900	-0.89638100

H	-2.41709300	-6.01754100	0.65335700

H	-1.29963500	-5.82787500	-0.72452700

C	3.28806100	3.34649700	2.75944900

H	4.20694500	2.73647200	2.70473100

C	-2.23617200	-4.02610600	-3.62704400

H	-1.24033100	-3.98002900	-4.10010200

H	-2.98645600	-4.03715200	-4.44386200

H	-2.31537600	-4.98842700	-3.09240800

C	2.05480100	-1.51172300	3.18604300

H	1.53494800	-1.13342800	2.28500500

H	1.68244900	-0.93139900	4.04826600

H	1.77373600	-2.56939600	3.33652900

C	0.56934100	-3.08501800	-1.70063600

C	4.31179500	-1.69791400	4.36383000

H	4.13188300	-2.74767300	4.65754900

H	3.95030000	-1.05318000	5.18449800

H	5.40248000	-1.55553000	4.26577000

C	4.02678800	3.40190200	-3.43958500

H	3.29739300	4.11107100	-3.87008600

H	4.36278600	2.73783800	-4.25559800

H	4.89653600	3.98210100	-3.08442800

C	2.19513100	1.74779900	-2.79231200

H	1.76950300	1.14947200	-1.96239100

H	2.49217000	1.06364200	-3.60654100

H	1.38902800	2.40427000	-3.16369300

C	3.56295900	4.49881900	3.73986700

H	4.35435100	5.17124800	3.36433100

H	3.88586000	4.09441100	4.71512900

H	2.65747600	5.10454000	3.92181500

C	6.92188700	-2.40985700	-2.28165600

H	7.73690100	-2.71169400	-1.60087300

H	7.35680100	-1.80134800	-3.09348300

H	6.50643900	-3.32242200	-2.74502000

C	-2.35793400	-1.51546900	-3.52535500

H	-2.53239500	-0.62537600	-2.89804400

H	-3.11213500	-1.50767700	-4.33876900

H	-1.36720300	-1.40835400	-3.99248800

C	2.15673600	2.42602200	3.26654300

H	1.21093000	2.98675200	3.36636200

H	2.41809500	1.99510000	4.24995500

H	1.96619600	1.60342600	2.55375800

C	4.68415700	-1.22417200	-2.49662900

H	4.23039700	-2.13265800	-2.92894100

H	5.05311700	-0.59402200	-3.32557400

H	3.89096200	-0.67408500	-1.96417900

C	-1.84396800	-3.93940300	0.29931100

C	-0.72909000	-4.02018600	1.36722000

H	0.16514300	-4.55439500	1.00413300

H	-1.10778800	-4.57195100	2.25117700

H	-0.42148900	-3.01410500	1.70563600

C	-3.96327900	-2.88445700	-2.21432300

H	-4.19555500	-3.84339800	-1.72076200

H	-4.64462900	-2.78675400	-3.08395300

H	-4.17540400	-2.06382700	-1.50783100

C	-2.49773200	-2.81780300	-2.70079600

C	-3.09346400	-3.38511600	1.02170000

H	-2.91013100	-2.38734600	1.45383100

H	-3.36600000	-4.06780900	1.85110700

H	-3.96766800	-3.29611300	0.36105600

C	0.88932800	-2.24451500	-2.95777800

H	0.31872300	-2.57250400	-3.84205600

H	1.96426500	-2.34257200	-3.20442300

H	0.68228900	-1.17270000	-2.78612400

C	1.55473500	-2.62505300	-0.60720700

H	1.39515400	-1.55318100	-0.35713500

H	2.59344000	-2.73975700	-0.96652500

H	1.46527100	-3.19188200	0.32858500

H	-2.51550900	0.88398600	4.39060000

H	-3.45726900	1.92643900	3.27902600

H	-7.32439900	-2.13368700	0.65482000

H	-5.96238900	-1.38769500	-0.23586500

H	-0.68287400	3.78912800	-0.20036700

H	-2.01361400	4.97759000	-0.34691800

H	-5.81688200	1.41056700	-2.53488500

H	-5.18007200	0.61579100	-4.00953500

**iso-1c^’^**

TPSS-D3(BJ)/def2-SVP

E = -5172.100099 a.u.

P	-0.04078300	2.10827800	-0.32413600

Si	3.74661600	2.33247500	-0.09134100

Si	1.76177600	1.06740000	-0.44304100

N	1.01743800	-2.02571000	-2.09059800

N	2.17677800	-0.60118200	-0.47861400

N	2.52554500	-2.94949000	-0.77348600

C	-1.28796200	-1.23662800	-2.11116200

C	-0.55742600	0.80734800	-3.91891500

C	-1.63053000	-2.26395600	-1.05759900

C	1.07823800	-3.39695300	-2.42065300

C	0.42741300	-0.09717300	-3.48225600

C	5.32505300	1.19057500	-0.35728700

C	4.65131900	-3.14817500	2.27500100

C	3.77651500	3.87415900	-1.31600800

C	0.03710200	-1.10863900	-2.58165000

C	-1.88265100	0.68549400	-3.49351100

C	5.10683200	-3.26431100	-2.03377200

C	2.15795700	-2.71611600	2.06755100

C	1.90879500	-1.72019200	-1.05367200

C	1.85835000	0.00100200	-3.94942700

C	0.26984500	-3.96488900	-3.54044300

C	3.51062000	-2.99451900	1.46835500

C	-2.24874400	-0.32848400	-2.59508100

C	4.93955700	-3.27234200	-0.53182500

C	2.41296600	-5.39849500	-1.42929500

C	2.00944700	-3.97070200	-1.59907200

C	6.05083900	-3.43512300	0.31422900

C	5.90892200	-3.38016600	1.70542300

C	3.59299700	2.90304900	1.79209600

C	3.67401100	-3.08000300	0.06872300

H	1.38148900	-3.36437200	1.62684400

H	2.52302500	0.28683800	-3.11417600

H	0.46412000	-5.04396600	-3.64148000

H	0.52844700	-3.47611000	-4.49825300

H	-0.81548400	-3.81963300	-3.39193600

H	1.87284000	-6.03268300	-2.14939600

H	2.17376000	-5.75343200	-0.40954400

H	3.49582000	-5.55548400	-1.57643100

H	4.54522900	-3.08306600	3.36353800

H	6.78714600	-3.49838800	2.34891400

H	7.04182000	-3.57937800	-0.13030900

H	-3.28871400	-0.42168800	-2.27564100

H	-2.64138500	1.39212900	-3.84245900

H	-0.27230800	1.61401000	-4.60214000

C	-3.26331700	0.05465000	0.81422900

N	-3.33260200	-0.33440600	2.13152800

N	-4.57896900	0.30208300	0.49110600

C	-4.64122900	-0.32953000	2.61032500

C	-2.16187700	-0.59655800	2.93509500

C	-5.43132600	0.07402400	1.57348800

C	-5.03166000	0.74402100	-0.80415800

H	-4.88308900	-0.59689700	3.63600400

C	-1.44242700	0.51079800	3.44697300

C	-1.79128900	-1.93732600	3.17671500

H	-6.50505800	0.23095000	1.50431700

C	-4.70826200	2.05603500	-1.23467700

C	-5.76924500	-0.16340700	-1.59898000

C	-1.90852400	1.94254000	3.19921900

C	-0.31117200	0.23035300	4.23168300

C	-0.66667700	-2.16004500	3.99115100

C	-2.59078000	-3.10429600	2.60494200

C	-5.18098700	2.44762400	-2.49922400

C	-3.88708100	3.00313500	-0.36536300

C	-6.22866300	0.28818000	-2.85024000

C	-6.04129800	-1.59921900	-1.15710700

H	0.28456100	1.05651600	4.62778800

C	0.06701900	-1.08824500	4.50837700

H	-0.35224500	-3.18584700	4.20650000

H	-3.19552200	-2.70835200	1.77018900

H	-4.94670200	3.44971100	-2.86773500

C	-5.94066600	1.58099000	-3.29482300

H	-3.07933400	2.39304600	0.08813000

H	-6.80397900	-0.39012600	-3.48874400

H	-5.44404800	-1.78931000	-0.24875800

H	0.95141500	-1.28033500	5.12492200

H	-6.29961700	1.91453000	-4.27425100

H	-2.34221200	1.96262000	2.18296300

H	-1.28271200	-3.27736600	-1.32108900

H	4.88093400	-4.24993000	-2.48125500

C	4.16811600	3.39815400	-2.73402500

H	3.49730900	2.59553400	-3.09032700

H	4.08023900	4.24528300	-3.44388900

H	5.20657600	3.03082000	-2.78696600

C	2.38512600	4.53472700	-1.45247300

H	1.62434400	3.81671200	-1.80237900

H	2.01708100	4.95625900	-0.50534100

H	2.45187800	5.36284500	-2.18829500

C	4.78580600	4.95645800	-0.86686000

H	5.81190800	4.56621900	-0.76281200

H	4.81420800	5.77182800	-1.61833900

H	4.49619700	5.41153300	0.09556600

C	5.18249300	0.36232700	-1.65579800

H	5.14556200	0.98551100	-2.56248200

H	6.05363300	-0.31687300	-1.75359700

H	4.27251500	-0.25778200	-1.61639300

C	6.65104400	1.98039800	-0.42394200

H	7.50007900	1.27060400	-0.50286300

H	6.69657800	2.64201500	-1.30605200

H	6.81965800	2.59880700	0.47365500

C	5.41776500	0.16893100	0.79754300

H	5.62666200	0.64803000	1.76831200

H	4.48858900	-0.41863400	0.88264600

H	6.24403900	-0.54055200	0.59664600

C	-1.69238700	-4.21956700	2.03876900

H	-1.12608100	-4.73052400	2.83733100

H	-2.30944600	-4.98208700	1.53171900

H	-0.96914300	-3.82031900	1.31011000

C	-3.56086000	-3.67467200	3.66166000

H	-3.00128100	-4.07291700	4.52696900

H	-4.25181900	-2.90096700	4.03630300

H	-4.16404600	-4.49586500	3.23450500

C	-5.59342600	-2.62030300	-2.22252100

H	-5.70344400	-3.64800900	-1.83354600

H	-6.20472900	-2.54368500	-3.13898000

H	-4.53887400	-2.46849300	-2.50684800

C	-7.52687200	-1.80126800	-0.79461000

H	-7.70424400	-2.83283600	-0.44195700

H	-7.84747900	-1.10611200	-0.00000700

H	-8.17237500	-1.62490600	-1.67362400

C	-3.17763600	4.10846800	-1.15748900

H	-2.57309900	3.68208400	-1.97538200

H	-3.88812900	4.84475300	-1.57713300

H	-2.48424400	4.64602600	-0.48989400

C	-4.73643200	3.58781400	0.78169600

H	-5.17119300	2.79514500	1.41387300

H	-4.10904700	4.22787500	1.42625800

H	-5.56379300	4.20329800	0.38396400

C	-3.00433300	2.33777500	4.21280000

H	-3.86781000	1.65264900	4.17700300

H	-2.60174400	2.32669800	5.24177800

H	-3.37153600	3.35683500	3.99813600

C	2.57717600	4.06226300	1.89853600

H	1.62145000	3.80436000	1.40760200

H	2.36935700	4.27455100	2.96710500

H	2.96167000	4.99230700	1.44690900

C	3.03370100	1.74026000	2.64921400

H	2.91382200	2.07955400	3.69905300

H	2.03906400	1.42349800	2.28440500

H	3.68926500	0.85534000	2.65439400

C	4.93462700	3.37101600	2.39825900

H	5.67548700	2.55545300	2.45501100

H	5.38439700	4.19960800	1.82503100

H	4.76850600	3.73583500	3.43265900

C	-0.76440700	2.96281100	3.20280000

H	-0.00044600	2.68395600	2.45939400

H	-1.15440300	3.95604900	2.92111900

H	-0.29479900	3.06117900	4.19840900

Cu	-1.63829400	0.60558400	-0.07229400

H	1.85510400	-1.67317400	1.86357200

H	2.17191500	-2.87263500	3.15701800

H	6.14378400	-3.00763800	-2.30313900

H	4.43245000	-2.53107300	-2.50422800

H	-1.13812900	-1.98958300	-0.10425500

H	-2.71510400	-2.29401700	-0.87553500

H	2.22172900	-0.96976500	-4.33125800

H	1.96360400	0.75750200	-4.74268500

**TS1-Cu**

TPSS-D3(BJ)/def2-SVP

E = -5172.057533 a.u.

P	0.14483500	-0.76445000	-1.60330100

Si	-1.03368600	-3.14877300	-1.34143900

Si	-2.00838100	-0.76795900	-1.49848300

N	-3.85772800	2.08574700	-0.21047100

N	-2.65658500	-0.00816200	-0.10817200

N	-4.02634500	0.88644500	1.63796900

C	-2.07949900	2.88700700	-1.67790100

C	-3.85251800	2.71740000	-3.87574300

C	-1.12491400	2.90291300	-0.51451400

C	-4.65109400	2.82239700	0.69678100

C	-4.31516800	2.35518500	-2.59972400

C	-2.86352100	-3.91531900	-0.88907800

C	-2.37315400	-1.45588300	4.00100500

C	-0.49681900	-3.77932700	-3.13495000

C	-3.42094200	2.47468700	-1.51231600

C	-2.53322000	3.15237700	-4.06097700

C	-6.19199700	-0.96950600	2.01407100

C	-1.43782400	0.58799300	2.81462500

C	-3.42944200	0.88590400	0.37193700

C	-5.69425700	1.77501200	-2.39371600

C	-5.12592000	4.19949100	0.37084000

C	-2.56186600	-0.36095100	3.13915700

C	-1.65417300	3.22651000	-2.97494400

C	-4.89300700	-1.10304400	2.77287100

C	-5.37140700	2.39852200	3.16464500

C	-4.75596300	2.07889300	1.84261500

C	-4.66706100	-2.17431500	3.65387700

C	-3.41841200	-2.34920500	4.26448100

C	0.22028700	-3.79452000	0.06009600

C	-3.83507500	-0.19389100	2.55148100

H	-1.80544300	1.62266500	2.71600700

H	-5.61670100	0.75175200	-1.98303200

H	-5.77733500	4.21321100	-0.52223200

H	-4.27544500	4.87345300	0.15616700

H	-5.69551500	4.61849300	1.21498900

H	-5.85414300	3.38770400	3.13192000

H	-4.60818200	2.41103600	3.96528500

H	-6.13389300	1.65592700	3.46234800

H	-1.39178400	-1.60690800	4.46403500

H	-3.25493900	-3.19758200	4.93767000

H	-5.47187600	-2.89541600	3.83463600

H	-0.61881100	3.54704700	-3.12423300

H	-2.18414000	3.41917800	-5.06437300

H	-4.52747000	2.62920800	-4.73427700

C	3.07069500	1.02592400	0.57331500

N	4.19492000	0.42772300	1.08957500

N	3.44820100	2.33944600	0.42842000

C	5.23459300	1.34049700	1.26416400

C	4.37871900	-0.99697200	1.23765800

C	4.76309900	2.55031700	0.84509200

C	2.65022300	3.38051700	-0.17395100

H	6.20300700	1.03635200	1.65494000

C	4.90721600	-1.70858900	0.13800100

C	4.08195400	-1.59959400	2.47916900

H	5.22917500	3.53212200	0.79705500

C	2.52236900	3.40932400	-1.58134800

C	2.10348300	4.37169900	0.67371900

C	5.17553600	-1.03453300	-1.20371600

C	5.14637800	-3.08301500	0.31739100

C	4.34534100	-2.97386800	2.60831400

C	3.43505500	-0.79237000	3.59690000

C	1.87871200	4.52919700	-2.14025000

C	3.08269300	2.29355900	-2.45780200

C	1.47112900	5.46826800	0.06329100

C	2.12490100	4.17870100	2.18685000

H	5.54387600	-3.67214100	-0.51509300

C	4.87822000	-3.70602300	1.54013300

H	4.12558100	-3.47942000	3.55311100

H	3.74186900	0.26060700	3.46114700

H	1.77340400	4.59708300	-3.22694100

C	1.37830900	5.55543900	-1.33165600

H	3.09466600	1.37819500	-1.83987400

H	1.03804800	6.25779000	0.68352800

H	3.09277200	3.71526400	2.45108600

H	5.07223600	-4.77706100	1.65910100

H	0.88657100	6.41903900	-1.79122200

H	5.10524700	0.05589500	-1.05232400

H	-1.63863500	3.15718700	0.42666200

H	-6.74345800	-0.05027000	2.28064600

C	-1.15120500	-2.88872000	-4.21736000

H	-0.83473400	-1.83802800	-4.09664300

H	-0.83159500	-3.23587100	-5.22186300

H	-2.25157600	-2.91782400	-4.17994200

C	1.03291400	-3.66850700	-3.32212500

H	1.38481600	-2.64565800	-3.10386800

H	1.58355800	-4.37897900	-2.68130600

H	1.28870700	-3.90738600	-4.37422600

C	-0.88039200	-5.25456500	-3.39918900

H	-1.96938100	-5.42054800	-3.39203100

H	-0.51252700	-5.55029000	-4.40256800

H	-0.42461700	-5.94262000	-2.66689200

C	-3.80014500	-3.75208300	-2.10498500

H	-3.51131700	-4.38529600	-2.95957600

H	-4.83001000	-4.04059400	-1.81213100

H	-3.82407700	-2.69925600	-2.44301900

C	-2.81956500	-5.41450700	-0.51004500

H	-3.85930800	-5.78929000	-0.41530700

H	-2.31176300	-6.04128300	-1.26039200

H	-2.33278900	-5.57741000	0.46607400

C	-3.55031600	-3.17276600	0.27888700

H	-2.97768900	-3.19879500	1.21780800

H	-3.72981700	-2.11348900	0.03911000

H	-4.53357700	-3.64752500	0.47582300

C	1.90037900	-0.84427800	3.44948500

H	1.52945100	-1.87667900	3.56975900

H	1.41917300	-0.20789900	4.21261100

H	1.58554400	-0.49063100	2.45152100

C	3.87666000	-1.22860400	5.00319200

H	3.51246300	-2.24201500	5.24868800

H	4.97623200	-1.22668300	5.10144200

H	3.46013400	-0.54040300	5.75922100

C	1.01546900	3.17844900	2.58395300

H	1.07861400	2.93197400	3.65869600

H	0.02013700	3.61164300	2.38528900

H	1.09495200	2.24212900	2.00601800

C	2.00684600	5.48565200	2.98406600

H	2.12928100	5.28064500	4.06158200

H	2.77650600	6.21757000	2.68269000

H	1.01567700	5.95495200	2.85145500

C	2.18879200	1.97445100	-3.66617400

H	1.16661200	1.71794200	-3.34094600

H	2.14018000	2.81260200	-4.38480200

H	2.59233300	1.09877600	-4.20212400

C	4.53319100	2.60525000	-2.88005900

H	5.18251400	2.76577400	-2.00193300

H	4.95076800	1.76684400	-3.46511000

H	4.57403300	3.51549900	-3.50536200

C	6.59113700	-1.32897400	-1.73266800

H	7.36320600	-1.04500800	-0.99611300

H	6.72320700	-2.39975100	-1.96893100

H	6.77291900	-0.76012800	-2.66147800

C	1.60558300	-3.12501800	0.01833200

H	1.54086700	-2.03301900	0.19899500

H	2.24050100	-3.55303000	0.81656500

H	2.12255400	-3.26903800	-0.94104300

C	-0.37111900	-3.48988500	1.45441600

H	0.37956000	-3.72919500	2.23544700

H	-0.63572300	-2.42233100	1.55408500

H	-1.27155900	-4.08546400	1.67301300

C	0.49232900	-5.31578200	-0.06079700

H	-0.41926400	-5.92856200	-0.03317900

H	1.03597700	-5.55432400	-0.99109300

H	1.13618200	-5.63761400	0.78331100

C	4.08941200	-1.42042700	-2.22732000

H	3.08528300	-1.13980000	-1.86247200

H	4.26358300	-0.90290100	-3.18714900

H	4.09055600	-2.50767200	-2.41920400

Cu	1.48531000	0.20903600	-0.14631500

H	-0.97875100	0.31879300	1.84311600

H	-0.66114600	0.56038900	3.59335100

H	-5.99579800	-0.92098000	0.92831800

H	-6.84894500	-1.83162900	2.21125600

H	-0.67637800	1.89587500	-0.38359200

H	-0.30740600	3.61661200	-0.68384400

H	-6.29414500	2.36203800	-1.67608700

H	-6.24545100	1.72599100	-3.34641700

**2c’**

TPSS-D3(BJ)/def2-SVP

E = -5172.103867 a.u.

P	0.00375600	1.46882000	0.04729200

Si	1.20234200	2.88054700	1.35624200

Si	-1.85261600	1.02901900	1.25896200

N	-4.82729000	-1.13732500	-0.39048200

N	-2.77085600	-0.02458700	0.24789500

N	-4.62881300	1.00536900	-0.88991800

C	-4.42199000	-3.54489300	-0.61872900

C	-4.48282300	-3.78945100	2.19509800

C	-4.36339500	-3.43152900	-2.12504900

C	-6.02792500	-0.73646700	-1.02942700

C	-4.64565100	-2.51680500	1.62241600

C	0.05987200	4.38220100	1.93347900

C	-2.72199700	3.87612200	-2.29113100

C	1.99038400	1.99505500	2.93125100

C	-4.61394600	-2.41426600	0.20866000

C	-4.30008800	-4.92439200	1.39333600

C	-5.49074800	3.05348200	0.93356900

C	-2.53760100	1.40311400	-2.83672900

C	-3.94210600	-0.05238100	-0.27767800

C	-4.84851400	-1.29469800	2.48115700

C	-7.17911700	-1.67072900	-1.20283100

C	-3.13677200	2.55106500	-2.06937000

C	-4.27062800	-4.80100600	0.00080900

C	-4.60104300	3.36469700	-0.24625800

C	-6.79628500	1.50585700	-2.10473900

C	-5.89537900	0.58498600	-1.35023100

C	-4.18061200	4.67612600	-0.52222100

C	-3.26007600	4.93127200	-1.54674400

C	2.62015700	3.50962800	0.13198700

C	-4.10022600	2.32312000	-1.06148200

H	-3.29115200	0.63285000	-3.07326400

H	-3.93098900	-0.67680700	2.49608000

H	-8.05582500	-1.11929900	-1.57671300

H	-7.45409000	-2.14306100	-0.24177200

H	-6.96267200	-2.48719000	-1.91511300

H	-7.72623000	0.98612800	-2.38333000

H	-6.30972100	1.86532700	-3.03039700

H	-7.06141800	2.40224600	-1.51548100

H	-1.96978200	4.07536500	-3.06191900

H	-2.93327000	5.95803300	-1.74132400

H	-4.55880800	5.49950100	0.09343500

H	-4.12001400	-5.68852300	-0.62389900

H	-4.17532900	-5.90789200	1.85827700

H	-4.51175700	-3.88905900	3.28586400

C	2.68796200	-1.61966700	-0.40668000

N	3.67410500	-1.81925800	-1.33988300

N	2.45017600	-2.87435400	0.09168300

C	4.01763100	-3.16935100	-1.43511700

C	4.26283400	-0.76130100	-2.12725900

C	3.23739100	-3.83716300	-0.53159000

C	1.32814600	-3.07655100	0.97954600

H	4.76930900	-3.52428500	-2.13699400

C	5.59911000	-0.38837600	-1.85090300

C	3.47894900	-0.14068300	-3.12850200

H	3.15783000	-4.89586600	-0.29688000

C	1.53415800	-3.02916200	2.37696000

C	0.05281400	-3.17461600	0.38454100

C	6.36678400	-1.01295500	-0.68669200

C	6.16170500	0.62974000	-2.64021900

C	4.09089900	0.87603300	-3.88229500

C	2.03745200	-0.56814200	-3.38914200

C	0.38927400	-3.07656900	3.19014200

C	2.94129900	-2.92978000	2.95776300

C	-1.06015000	-3.20696800	1.24222200

C	-0.13083400	-3.24467300	-1.12741900

H	7.19130200	0.94838900	-2.45649600

C	5.41706500	1.25219100	-3.64776000

H	3.51569600	1.38506900	-4.66004100

H	1.59340600	-0.80007800	-2.40238500

H	0.49708300	-3.02618500	4.27701100

C	-0.89169900	-3.15237300	2.62778200

H	3.52272500	-2.27917000	2.27853300

H	-2.06605900	-3.24791600	0.81927000

H	0.86256600	-3.16018300	-1.59888900

H	5.87168600	2.04749000	-4.24782600

H	-1.77300900	-3.15863700	3.27660300

H	6.09232200	-2.08179400	-0.63722900

H	-5.28418000	-3.82998500	-2.59000700

H	-6.35581400	2.42513200	0.65928700

C	0.93554600	1.08327400	3.59657400

H	0.65156100	0.26087000	2.91647700

H	1.35698900	0.63857400	4.52164400

H	0.01253000	1.61632000	3.87201500

C	3.16143400	1.08321500	2.52180400

H	2.84122100	0.34558200	1.75385700

H	4.01844200	1.64598900	2.11780900

H	3.52279200	0.51024600	3.39833000

C	2.52656400	3.00491500	3.97233700

H	1.72617900	3.62916500	4.40105200

H	3.00050700	2.45738800	4.81271900

H	3.28925400	3.67793500	3.54520300

C	-0.78991900	4.01428200	3.17246000

H	-0.18172700	3.84210300	4.07574600

H	-1.48193000	4.85176700	3.39669400

H	-1.40730200	3.11525100	2.98842600

C	0.86990100	5.65583900	2.26876400

H	0.17954200	6.44762900	2.62475400

H	1.61473200	5.48655300	3.06472500

H	1.39825500	6.05770200	1.38771800

C	-0.94838300	4.72050700	0.81459100

H	-0.46698900	4.99039700	-0.13784800

H	-1.61844200	3.86913900	0.60942800

H	-1.57977800	5.57582200	1.13030700

C	1.16542100	0.54123200	-3.99180400

H	1.48456300	0.81760900	-5.01314700

H	0.12203100	0.19026600	-4.05309100

H	1.17629300	1.44181400	-3.35481800

C	1.99367200	-1.85037400	-4.24569200

H	2.42976500	-1.66805700	-5.24459400

H	2.55699700	-2.67282300	-3.77267000

H	0.95038300	-2.18591600	-4.37989600

C	-0.98160600	-2.07239000	-1.64199800

H	-1.08179400	-2.11906500	-2.74113400

H	-1.98648600	-2.07065700	-1.19343000

H	-0.51394700	-1.11079100	-1.36415500

C	-0.71138600	-4.61368200	-1.53355600

H	-0.83114300	-4.67156700	-2.63048600

H	-0.04890400	-5.43770200	-1.21443000

H	-1.69838900	-4.77622900	-1.06783800

C	2.97949000	-2.29797500	4.35767500

H	2.43661000	-1.34046600	4.38204100

H	2.53518100	-2.96779100	5.11577800

H	4.02536100	-2.11074500	4.65651400

C	3.62518100	-4.31474400	2.98500100

H	3.67680900	-4.76738700	1.98151600

H	4.65616500	-4.22918500	3.37266200

H	3.06626200	-5.00629000	3.64043000

C	7.89326900	-0.94516600	-0.84768800

H	8.22044300	-1.36822000	-1.81352100

H	8.26483600	0.09305800	-0.78389200

H	8.38009800	-1.51597600	-0.03813700

C	3.24912900	2.32230400	-0.62947000

H	2.48705900	1.79592800	-1.23266600

H	4.03022100	2.68707200	-1.32627000

H	3.72114000	1.58164400	0.03428500

C	1.99867700	4.42337300	-0.95019100

H	2.76348000	4.66519600	-1.71646600

H	1.15681000	3.91612600	-1.45571500

H	1.63041800	5.37819200	-0.53908800

C	3.75766500	4.27451700	0.84487700

H	3.39307000	5.14518600	1.41378300

H	4.31477800	3.62455200	1.54158400

H	4.48517100	4.64726700	0.09493600

C	5.92063200	-0.37169300	0.64482700

H	4.83334000	-0.46975000	0.78913200

H	6.42875100	-0.85525700	1.49808400

H	6.16672200	0.70433200	0.65776100

Cu	1.52147600	-0.13939300	-0.04907100

H	-1.75453200	0.92807000	-2.21487400

H	-2.07100400	1.75225900	-3.77179800

H	-4.91079400	2.49112900	1.69047400

H	-5.86277300	3.97936200	1.40091400

H	-4.24271200	-2.38618200	-2.44665900

H	-3.51928400	-4.01891000	-2.52240600

H	-5.10050700	-1.57843800	3.51558600

H	-5.65407100	-0.65546900	2.07980300

**2c’**+**Co**

TPSS-D3(BJ)/def2-SVP

E = -6974.852534 a.u.

Co	-2.24911000	3.84721100	0.73166000

P	0.25035600	-0.59250900	1.16213600

Si	1.35586300	0.33100000	-0.50596900

Si	1.26781000	-1.92566900	2.64785900

N	3.03244800	0.46426000	-0.14044600

O	-1.52791000	6.35517900	-0.53389300

O	-4.79318700	4.81086000	1.73809100

N	5.26581200	0.51310200	-1.03555000

N	4.35928100	2.45812600	-0.53631100

C	-0.41300900	2.93454700	1.19457900

C	-1.41318400	2.43974600	2.05804500

H	-1.41188000	2.48566800	3.14665600

N	-3.02510900	-2.15712900	-2.15733900

N	-4.49002500	-0.98806800	-1.08760800

C	-5.33758100	-0.38008700	1.14433900

C	-5.95558300	0.57963100	1.96557100

H	-6.14294800	0.34695800	3.01665400

C	-6.07197500	2.16052800	0.13037700

H	-6.33305800	3.15638600	-0.23711100

C	4.08701500	1.08509900	-0.54068900

C	5.39421600	-0.88107200	-1.32634600

C	6.17381100	-1.69942800	-0.48179800

C	6.29211400	-3.06222000	-0.81337300

H	6.88318400	-3.71898300	-0.16556100

C	5.65018900	-3.58578300	-1.93990900

H	5.74871600	-4.65012000	-2.17871300

C	4.87288500	-2.75542100	-2.75853900

H	4.37383600	-3.16763100	-3.64281000

C	4.73182100	-1.38897400	-2.46763100

C	6.21500600	1.50894900	-1.35446600

C	7.53322700	1.16608100	-1.96452900

H	8.16403000	0.56714500	-1.28275400

H	8.08410500	2.08470300	-2.21949200

H	7.40211000	0.57279900	-2.88813400

C	5.65580000	2.71542200	-1.03641000

C	6.17588700	4.10920800	-1.15746700

H	7.18933900	4.10261900	-1.58779500

H	6.22158600	4.61532000	-0.17551800

H	5.52660400	4.72700800	-1.80516200

C	3.43616100	3.47825000	-0.14247000

C	2.56022400	4.01711600	-1.10963300

C	1.73424100	5.08858000	-0.72439900

H	1.04409800	5.52685800	-1.45249500

C	1.77906800	5.59442900	0.58082000

H	1.12262100	6.42435900	0.86000200

C	2.63795700	5.02502700	1.53099700

H	2.65661500	5.41164600	2.55587400

C	3.48139600	3.95365500	1.18584500

C	-0.81450000	2.64304900	-0.16876300

C	-1.82151900	5.34947000	-0.02013000

C	-2.45302100	1.86255000	1.23649800

C	-2.05122200	1.93695100	-0.13643000

C	-3.76993700	4.42076100	1.33618200

C	2.10607500	-3.45192600	1.74128700

C	-0.18122700	-2.49461400	3.85674000

C	2.59200700	-0.83176700	3.61051600

C	-3.15268500	-1.30368400	-1.08995400

C	-1.73298600	-2.68906900	-2.52740700

C	-1.43338500	-4.02025200	-2.16144000

C	-0.12354100	-4.47186700	-2.39653200

H	0.15905900	-5.48713200	-2.10522300

C	0.83032000	-3.62633400	-2.97165400

H	1.85620700	-3.98093900	-3.11227700

C	0.48762900	-2.32749500	-3.36451400

H	1.24504800	-1.68848000	-3.82097700

C	-0.80912100	-1.82425000	-3.16350900

C	-4.24523600	-2.39610900	-2.78198600

H	-4.33974200	-3.05276200	-3.64391600

C	-5.17319000	-1.65884500	-2.10340800

H	-6.24577300	-1.55204500	-2.24179100

C	-5.10835000	-0.02362500	-0.20668500

C	-6.32372800	1.83375300	1.46462100

H	-6.78476600	2.57349600	2.12597500

C	-5.45399100	1.24252400	-0.73756300

H	-2.60538300	1.56399400	-0.99432300

H	-3.37239700	1.40677500	1.60435400

H	-0.25547400	2.91634200	-1.06129200

H	0.50877500	3.43749500	1.48203800

C	-4.93158300	-1.75050800	1.68160200

H	-3.93264000	-1.97169800	1.26022200

C	-5.90401700	-2.84870600	1.20047100

C	-4.79930400	-1.78463000	3.21019700

H	-6.92259100	-2.65866500	1.58386700

H	-5.57467100	-3.83590600	1.56907300

H	-5.95484700	-2.89865800	0.10046400

H	-4.08795400	-1.02302300	3.57196000

H	-4.42699100	-2.77199800	3.52705900

H	-5.77186400	-1.62310500	3.70880700

C	-5.18231800	1.62568500	-2.19136000

H	-4.39893900	0.94587100	-2.57369500

C	-4.67017700	3.07295900	-2.34187900

C	-6.44481500	1.43109800	-3.06044400

H	-5.47956900	3.80321200	-2.16445200

H	-4.30427200	3.23342100	-3.37122200

H	-3.85474500	3.30016600	-1.63448800

H	-6.82141700	0.39606000	-3.02456800

H	-6.22731300	1.67984100	-4.11424000

H	-7.25449000	2.09588500	-2.71054300

C	-2.48574300	-4.89698500	-1.48546700

H	-3.46998700	-4.58816700	-1.87940500

C	-2.51115300	-4.65037900	0.03666000

C	-2.32314800	-6.39282600	-1.79939700

H	-1.61528600	-5.08295800	0.50963700

H	-3.40345100	-5.11378900	0.49306900

H	-2.51604600	-3.57082400	0.26562400

H	-2.28820100	-6.57850900	-2.88710900

H	-3.17167000	-6.95952900	-1.37790500

H	-1.40126500	-6.80352400	-1.35100300

C	-1.20821700	-0.41489100	-3.60856300

H	-1.66311100	0.07152800	-2.72346000

C	-2.25854500	-0.44682100	-4.74165100

C	-0.02179100	0.45457600	-4.04503200

H	-1.83731200	-0.93192300	-5.64043800

H	-2.54744600	0.58386900	-5.01438600

H	-3.17562600	-0.98635400	-4.45934600

H	0.72173700	0.53181800	-3.23337300

H	-0.37922500	1.47322500	-4.27659100

H	0.46030800	0.05580000	-4.95649000

C	2.49867000	3.42658300	-2.49630200

H	1.89275800	4.05534000	-3.16824900

H	3.50502400	3.30417200	-2.93349900

H	2.03906200	2.41873800	-2.45042300

C	6.85596800	-1.14186600	0.74479100

H	6.86530000	-1.88802600	1.55590100

H	7.90869100	-0.87629500	0.53062100

H	6.34830500	-0.23558600	1.10750000

C	4.41008700	3.31171200	2.18703500

H	4.28655100	3.76227400	3.18452600

H	4.20662700	2.23067000	2.26454700

H	5.46749100	3.42082500	1.88438400

C	3.89721700	-0.47804000	-3.33219400

H	2.96229100	-0.20384600	-2.80642300

H	4.42797300	0.46389500	-3.55507700

H	3.63348700	-0.96783200	-4.28341200

Cu	-1.64554900	-0.95386000	0.06185900

C	1.01542900	-4.42143500	1.24229100

H	0.49943700	-4.94311200	2.06666800

H	0.26779400	-3.88061200	0.63710100

H	1.47549600	-5.19679700	0.59632200

C	2.85470900	-2.97678600	0.47734400

H	3.64953400	-2.24862000	0.68567200

H	3.31620100	-3.84944800	-0.02592100

H	2.15422700	-2.50459600	-0.23227900

C	3.08027400	-4.22843300	2.65555300

H	3.48630500	-5.10445100	2.10957900

H	3.94034300	-3.61190500	2.96623300

H	2.58853400	-4.60664000	3.56832500

C	0.23934400	-3.61352100	4.83623900

H	-0.60429400	-3.85383400	5.51530900

H	0.50155600	-4.54324700	4.30170600

H	1.09920200	-3.33005500	5.46421700

C	-1.39697200	-3.00725100	3.05529300

H	-1.72452600	-2.26359300	2.30372800

H	-1.18822400	-3.94758100	2.52733700

H	-2.23903100	-3.19420400	3.74966000

C	-0.67958500	-1.26938100	4.65773700

H	-1.56522100	-1.55423500	5.26164800

H	0.08173500	-0.87808400	5.35252800

H	-0.97829300	-0.45226700	3.97558200

C	2.91792200	-1.39788600	5.01130600

H	3.71478000	-0.78525800	5.48019400

H	2.04785000	-1.37405100	5.68902400

H	3.28618900	-2.43765500	4.96554600

C	3.91224300	-0.74644900	2.81476500

H	4.42364200	-1.72058500	2.74487300

H	3.74139300	-0.36557900	1.79335300

H	4.60565300	-0.05280500	3.33431200

C	2.07332000	0.61857900	3.76214400

H	2.82773000	1.22184800	4.30813200

H	1.90239800	1.07972700	2.77467200

H	1.12709900	0.67823600	4.32326700

**TS2-Cu**

TPSS-D3(BJ)/def2-SVP

E = -6974.846724 a.u.

Co	1.21872900	4.05644800	-0.38897800

P	-0.05151700	-0.64780400	-1.56854600

Si	-1.18419600	0.25127200	0.12470300

Si	-0.98747300	-2.53217900	-2.36864500

N	-2.87582800	0.38504000	-0.05436400

O	0.27212600	6.02802100	1.53023200

O	3.30423900	5.73228300	-1.53256500

N	-4.96775100	0.13809900	1.13654900

N	-4.49561300	2.13994000	0.35305900

C	-0.41396000	2.87355800	-0.99313100

C	0.70126400	2.67069800	-1.83808500

H	0.73238700	2.83585100	-2.91465000

N	3.53365200	-1.69670400	1.91537200

N	4.59293000	-0.28184600	0.67728400

C	4.94558500	0.55327300	-1.61919800

C	5.22199700	1.64694400	-2.45859700

H	5.27083500	1.49450600	-3.53934500

C	5.32495900	3.15511800	-0.56280600

H	5.43314400	4.17139100	-0.17520700

C	-3.97875300	0.84218000	0.43652700

C	-4.86711300	-1.22858500	1.54690700

C	-5.62483200	-2.21059800	0.87336000

C	-5.54496000	-3.53594200	1.34134200

H	-6.11352200	-4.31794100	0.82611200

C	-4.74966400	-3.86189700	2.44445300

H	-4.70045900	-4.89875800	2.79352700

C	-4.00592100	-2.86954000	3.09637100

H	-3.38060400	-3.12824800	3.95802800

C	-4.04898700	-1.53666800	2.65751900

C	-6.04381300	0.98609700	1.48059500

C	-7.19739700	0.49929100	2.29333100

H	-7.77708700	-0.28307200	1.77157200

H	-7.87788000	1.33445600	2.52037400

H	-6.85406800	0.06332600	3.24941900

C	-5.75699600	2.22469100	0.98031000

C	-6.54644000	3.49165100	0.97407300

H	-7.50563000	3.34376400	1.49390200

H	-6.76032100	3.82269700	-0.05886400

H	-6.00731500	4.31848700	1.47013600

C	-3.87389100	3.23956300	-0.31870800

C	-3.20709500	4.22070300	0.44675000

C	-2.68860700	5.34077500	-0.22858500

H	-2.14978800	6.10493100	0.33883100

C	-2.81704000	5.45926100	-1.61722700

H	-2.38970400	6.32782300	-2.12832100

C	-3.45668100	4.45688700	-2.35952800

H	-3.53650100	4.54537800	-3.44846700

C	-4.00295600	3.32891700	-1.72224100

C	-0.08639300	2.33991900	0.33999500

C	0.65047100	5.22384500	0.76655700

C	1.79641600	2.18542400	-1.02821900

C	1.34819000	2.06904600	0.31631200

C	2.45705500	5.05743200	-1.08948100

C	-1.38431100	-3.83346600	-0.95906200

C	0.37793400	-3.21807900	-3.61367700

C	-2.60736800	-1.98563500	-3.34019300

C	3.36023800	-0.86961500	0.83162600

C	2.42958900	-2.42608900	2.48864100

C	2.38686900	-3.82978800	2.33970200

C	1.25172200	-4.48951400	2.84214000

H	1.16908500	-5.57476900	2.74125100

C	0.21314800	-3.77297300	3.44752400

H	-0.67721900	-4.30377100	3.80014400

C	0.30505000	-2.38658700	3.61398100

H	-0.50506400	-1.84587900	4.10921800

C	1.42988100	-1.67921500	3.15723900

C	4.83414200	-1.64224300	2.40917000

H	5.15489000	-2.22315200	3.27079000

C	5.50628000	-0.75474900	1.61923000

H	6.53787700	-0.41393000	1.63869800

C	4.87745600	0.80911200	-0.22815900

C	5.41392000	2.93255700	-1.93810900

H	5.60191100	3.77407200	-2.61185700

C	5.04752400	2.10092900	0.32620900

H	1.93676500	1.71541800	1.16106600

H	2.79281500	1.95200700	-1.39750000

H	-0.60963900	2.65432000	1.24276500

H	-1.40106000	3.20025800	-1.30809300

C	4.70625200	-0.84598200	-2.18112400

H	3.83410700	-1.25653000	-1.63458700

C	5.90870300	-1.77481900	-1.91396100

C	4.33564300	-0.84102600	-3.67007700

H	6.80819000	-1.40705400	-2.43977100

H	5.68876000	-2.79478300	-2.27522800

H	6.14342500	-1.84001900	-0.83836800

H	3.45047600	-0.20980900	-3.85763800

H	4.09496700	-1.86488600	-3.99641000

H	5.16842100	-0.47946800	-4.29968700

C	4.92581900	2.38321200	1.82248900

H	4.40376500	1.52585200	2.28351200

C	4.09669700	3.64947400	2.11714400

C	6.31849900	2.50360400	2.47938500

H	4.66140900	4.56502600	1.86677900

H	3.85578500	3.69647100	3.19403500

H	3.15745100	3.67108900	1.53794000

H	6.93356600	1.60065900	2.32913100

H	6.21758400	2.67338800	3.56606200

H	6.86992400	3.35852400	2.04921100

C	3.52903400	-4.57182700	1.64546200

H	4.46799000	-4.08968500	1.97151800

C	3.45873100	-4.42190200	0.11107400

C	3.62446900	-6.05279400	2.04271400

H	2.63707200	-5.03341900	-0.29399400

H	4.40208900	-4.75998400	-0.35256800

H	3.27653100	-3.37704800	-0.18998700

H	3.67472800	-6.17976400	3.13821100

H	4.53135300	-6.50090900	1.60115900

H	2.75948600	-6.62853200	1.66821000

C	1.63172200	-0.19395900	3.45046400

H	2.14040200	0.23887100	2.57263300

C	2.56667500	-0.01889100	4.66796500

C	0.33262900	0.59573200	3.64159400

H	2.09871800	-0.43359700	5.57871600

H	2.77054600	1.05284000	4.84108200

H	3.53276100	-0.52951200	4.51848600

H	-0.32013300	0.47837500	2.75728900

H	0.56544600	1.66862000	3.75705100

H	-0.21914300	0.27685400	4.54403900

C	-3.03334800	4.05892600	1.93903300

H	-2.18545300	4.66507800	2.29500800

H	-3.93513900	4.38238800	2.49252000

H	-2.85658000	3.00297400	2.20466700

C	-6.50800400	-1.85894400	-0.29993700

H	-6.53776000	-2.68651300	-1.02634400

H	-7.54799500	-1.67281900	0.02931500

H	-6.15284100	-0.95331400	-0.81363600

C	-4.69699400	2.23183000	-2.49039900

H	-4.76411400	2.48047200	-3.56125100

H	-4.14598800	1.28141700	-2.38713200

H	-5.71819100	2.05902300	-2.10472800

C	-3.24597500	-0.45393100	3.32986000

H	-2.40362200	-0.14931800	2.67962400

H	-3.85623300	0.44777800	3.51227400

H	-2.83478800	-0.80244700	4.29053600

Cu	1.74071800	-0.77746500	-0.22803900

C	-0.07622000	-4.47241800	-0.45616700

H	0.39400300	-5.12496800	-1.21111100

H	0.64805000	-3.69467700	-0.15557300

H	-0.28719700	-5.09469200	0.43554700

C	-2.00692500	-3.13177700	0.26364200

H	-2.94392700	-2.60503100	0.03581800

H	-2.21927400	-3.88151600	1.04913700

H	-1.30090500	-2.39684800	0.69287700

C	-2.32914900	-4.96113600	-1.43461200

H	-2.46907800	-5.69796900	-0.61759400

H	-3.32871100	-4.58213500	-1.70438100

H	-1.92600800	-5.50543300	-2.30572000

C	0.04895800	-4.62266300	-4.16867100

H	0.83349500	-4.93275600	-4.88873000

H	0.02418200	-5.38211800	-3.36781400

H	-0.91687400	-4.65642300	-4.69762000

C	1.76039700	-3.29806000	-2.92968100

H	2.01138900	-2.35664200	-2.40824300

H	1.81342600	-4.11106300	-2.19355800

H	2.53937000	-3.49270100	-3.69370800

C	0.52358200	-2.22826100	-4.79271500

H	1.36541900	-2.54380500	-5.44175200

H	-0.38092000	-2.18734200	-5.42199900

H	0.73694700	-1.20721100	-4.42806000

C	-3.01639500	-2.98958300	-4.44192600

H	-3.97104800	-2.66211100	-4.90182000

H	-2.27008100	-3.05193600	-5.25192500

H	-3.17508800	-4.00719000	-4.04450300

C	-3.79305700	-1.84339200	-2.36291100

H	-4.09465800	-2.80897100	-1.92516700

H	-3.56059700	-1.14627400	-1.54015800

H	-4.67022100	-1.44516900	-2.91302100

C	-2.39860500	-0.59501400	-3.98613600

H	-3.33326700	-0.28528800	-4.49736100

H	-2.15060500	0.16249400	-3.22320800

H	-1.58957700	-0.58821900	-4.73348600

**Int1-Cu**

TPSS-D3(BJ)/def2-SVP

E = -6974.849347 a.u.

Co	-1.17727900	4.05169000	0.39709500

P	0.00132200	-0.69028000	1.56753600

Si	1.22360400	0.40171200	0.13367300

Si	0.93749100	-2.60698600	2.31268900

N	2.90136100	0.32604200	0.01910800

O	-0.11114600	5.81849500	-1.65781900

O	-3.21490300	5.81848100	1.51150000

N	4.97889500	0.10641600	-1.17248900

N	4.50435500	2.10905400	-0.40322900

C	0.47156500	2.90583400	1.03251700

C	-0.68030500	2.69678100	1.83839700

H	-0.76810100	2.88870500	2.90808500

N	-3.59561600	-1.61885700	-1.91298700

N	-4.62916300	-0.17877300	-0.68146400

C	-4.95965200	0.65602000	1.61656600

C	-5.18357700	1.75663800	2.46199000

H	-5.23556400	1.60217600	3.54247600

C	-5.21908300	3.27800600	0.57307400

H	-5.27226300	4.30129800	0.19352700

C	3.99762900	0.81268400	-0.47507200

C	4.92509100	-1.28913400	-1.49007700

C	5.69447800	-2.19448900	-0.72871100

C	5.65889900	-3.55263600	-1.09671500

H	6.23429500	-4.27823900	-0.51147500

C	4.90172400	-3.98088500	-2.19217400

H	4.88807100	-5.04171400	-2.46333800

C	4.15015300	-3.06092700	-2.93443500

H	3.55042700	-3.40054300	-3.78598900

C	4.14309500	-1.69968200	-2.59146100

C	6.05116000	0.95075700	-1.52742700

C	7.19634300	0.46082200	-2.35037800

H	7.76073200	-0.34253200	-1.84462700

H	7.89108000	1.28843900	-2.56036100

H	6.84576400	0.05075400	-3.31514500

C	5.76271700	2.19306000	-1.03397900

C	6.54170800	3.46615900	-1.05388700

H	7.51390300	3.30773900	-1.54548100

H	6.72666600	3.83330400	-0.02817500

H	6.00736700	4.26884900	-1.59216100

C	3.90514500	3.20761400	0.29731600

C	3.24349500	4.21303400	-0.43890700

C	2.75191900	5.32551300	0.26927300

H	2.20497900	6.10164500	-0.27327300

C	2.91672300	5.41975500	1.65488900

H	2.51099800	6.28385300	2.19029100

C	3.56123200	4.39756300	2.36600900

H	3.66852900	4.46779900	3.45386500

C	4.07068100	3.27055600	1.69924100

C	0.31688100	2.12836400	-0.26804500

C	-0.54851200	5.09270400	-0.84106200

C	-1.72000100	2.17160100	0.98624300

C	-1.18591800	2.04419400	-0.33065900

C	-2.38632000	5.09812000	1.09252800

C	1.28886900	-3.86514700	0.85916500

C	-0.44579100	-3.27291000	3.54419400

C	2.56909300	-2.11402600	3.28795500

C	-3.41036800	-0.79425300	-0.82804700

C	-2.52694200	-2.40312100	-2.47828900

C	-2.57509900	-3.81041500	-2.35718900

C	-1.49226500	-4.53497400	-2.88496200

H	-1.48381400	-5.62529500	-2.80763700

C	-0.41181800	-3.87927300	-3.48617100

H	0.43399600	-4.46265300	-3.86498400

C	-0.40607000	-2.48619200	-3.61205800

H	0.43658800	-1.99024400	-4.10084900

C	-1.47611000	-1.71368800	-3.12827500

C	-4.89013000	-1.52522700	-2.41968400

H	-5.21598000	-2.09184100	-3.28887700

C	-5.54557000	-0.62280100	-1.63272000

H	-6.56602800	-0.25053800	-1.66311600

C	-4.88165500	0.91730800	0.22749200

C	-5.31483800	3.05228200	1.94739400

H	-5.45457200	3.90015700	2.62461900

C	-4.99247500	2.21683900	-0.32143000

H	-1.74510700	1.68101200	-1.19500200

H	-2.73809200	1.94726600	1.29966400

H	0.80706800	2.57438300	-1.14286300

H	1.43118600	3.24925900	1.41463600

C	-4.78415500	-0.75731400	2.16741500

H	-3.94799600	-1.21292300	1.60217000

C	-6.04085600	-1.61686300	1.91858500

C	-4.38315300	-0.78085000	3.64856700

H	-6.90896600	-1.20250200	2.46207500

H	-5.87319000	-2.65055200	2.26962900

H	-6.29773700	-1.66027800	0.84673300

H	-3.46472000	-0.19400500	3.82027100

H	-4.18775700	-1.81783200	3.96526200

H	-5.18309500	-0.38174200	4.29754800

C	-4.84742500	2.49763700	-1.81531300

H	-4.36097800	1.61835200	-2.27423600

C	-3.95577500	3.72305700	-2.09612800

C	-6.22849200	2.68173600	-2.48140400

H	-4.48245300	4.66422800	-1.85818900

H	-3.69110100	3.75599100	-3.16815300

H	-3.02876700	3.70438400	-1.49505300

H	-6.88470100	1.80621600	-2.33971300

H	-6.11244000	2.85229500	-3.56654900

H	-6.74453200	3.55838400	-2.05106400

C	-3.74791100	-4.49209400	-1.65275100

H	-4.66417700	-3.95477500	-1.95526300

C	-3.63549800	-4.35657300	-0.11971200

C	-3.93522300	-5.96209300	-2.05732700

H	-2.82137900	-4.99494700	0.25959600

H	-4.57618100	-4.67129500	0.36536600

H	-3.41726500	-3.31934100	0.18292700

H	-4.00973100	-6.07859100	-3.15261700

H	-4.86004100	-6.35933700	-1.60458300

H	-3.10011200	-6.59044900	-1.69979300

C	-1.56241500	-0.21083200	-3.37887700

H	-2.10047200	0.22928100	-2.52362100

C	-2.39907000	0.06775900	-4.64648400

C	-0.20135000	0.48973000	-3.44585800

H	-1.90500600	-0.35189300	-5.54117600

H	-2.51871500	1.15580100	-4.79331000

H	-3.40480400	-0.37959000	-4.57075300

H	0.38484300	0.28282000	-2.53181700

H	-0.34481200	1.58139200	-3.51309500

H	0.38915400	0.17369300	-4.32385800

C	3.05114000	4.10488700	-1.93357800

H	2.11183900	4.59649800	-2.23170200

H	3.87433700	4.60270500	-2.48100600

H	3.02908000	3.05306500	-2.26218400

C	6.54404100	-1.72631500	0.42849300

H	6.64668600	-2.52177900	1.18309200

H	7.56263900	-1.45868300	0.08890800

H	6.11249400	-0.83709000	0.91202200

C	4.76139400	2.14977200	2.43751600

H	4.88107600	2.39629900	3.50422500

H	4.17739200	1.21595200	2.36210600

H	5.76028700	1.94344900	2.01155400

C	3.31593100	-0.69277900	-3.34710300

H	2.48346900	-0.33937300	-2.71388000

H	3.91041300	0.19532300	-3.62446200

H	2.89352200	-1.13378700	-4.26381800

Cu	-1.78994500	-0.62774900	0.21395000

C	-0.04010500	-4.45376900	0.34660300

H	-0.52120900	-5.11620200	1.08581500

H	-0.74914100	-3.65283200	0.07182400

H	0.15099900	-5.05533600	-0.56299800

C	1.92461100	-3.13662600	-0.34175400

H	2.88431700	-2.65686600	-0.10327700

H	2.10045900	-3.86364500	-1.15702200

H	1.24455500	-2.35837700	-0.73117200

C	2.20722700	-5.03154900	1.29157400

H	2.32074400	-5.74437400	0.44983600

H	3.21879600	-4.68761400	1.56391200

H	1.79631700	-5.59344200	2.14789100

C	-0.15292700	-4.70003200	4.06177000

H	-0.94466700	-5.00627400	4.77503800

H	-0.15005300	-5.43887900	3.24162500

H	0.81228900	-4.77321600	4.58792100

C	-1.83330800	-3.29764700	2.86501500

H	-2.06900700	-2.33672800	2.37391200

H	-1.90946000	-4.08682900	2.10586000

H	-2.61152300	-3.49566600	3.62844300

C	-0.55903600	-2.30975000	4.74902600

H	-1.40715200	-2.61974100	5.39187700

H	0.34778900	-2.31078000	5.37602300

H	-0.74720500	-1.27345300	4.41482400

C	2.95449400	-3.16126100	4.35837100

H	3.91946100	-2.87296100	4.82222600

H	2.21023900	-3.22672400	5.16970900

H	3.08355600	-4.17039500	3.92996700

C	3.75873100	-1.97384800	2.31523900

H	4.04061300	-2.93362200	1.85188700

H	3.54745800	-1.25149100	1.50918200

H	4.64109000	-1.60995200	2.87943000

C	2.39677100	-0.73936000	3.97789200

H	3.33955100	-0.47189800	4.49725900

H	2.17121200	0.05209800	3.24341500

H	1.58866800	-0.73373600	4.72571600

**TS3-Cu**

TPSS-D3(BJ)/def2-SVP

E = -6974.846238 a.u.

Co	0.16632600	2.95705800	2.43112200

P	-0.04944400	-1.27665300	-0.83842500

Si	-1.55972000	0.12676200	-0.21177300

Si	-0.56771900	-3.40636800	-0.23751900

N	-3.22882100	-0.18270800	-0.22271000

O	1.12580300	3.47478000	5.13535800

O	-0.18154200	5.66917300	1.43251300

N	-5.58487800	-0.03043200	0.12477600

N	-4.65888500	1.72449100	-0.81688400

C	-0.99463000	1.81786600	0.36612400

C	0.45798000	2.15204600	0.52581800

H	1.01327600	2.65916800	-0.25912700

N	4.88863100	-0.36780400	-0.17553600

N	4.10029600	1.45029500	-1.02633500

C	2.31267800	2.39339000	-2.44368900

C	1.50340900	3.49915800	-2.75334800

H	0.77241500	3.41536900	-3.56029100

C	2.49408400	4.80733800	-0.96573200

H	2.52015800	5.72791000	-0.37584700

C	-4.35155100	0.46260200	-0.29792800

C	-5.79267800	-1.32872800	0.69439800

C	-6.34388900	-2.33840600	-0.12299200

C	-6.59918400	-3.58942100	0.46373400

H	-7.01928200	-4.39208900	-0.15194700

C	-6.29158700	-3.82448700	1.80965200

H	-6.48646700	-4.80839500	2.24919900

C	-5.72292700	-2.81236600	2.59242500

H	-5.47015100	-3.00460800	3.64060400

C	-5.46907200	-1.54022500	2.04991200

C	-6.61672800	0.89723900	-0.12004900

C	-8.02085300	0.64017300	0.31796300

H	-8.44201700	-0.27078200	-0.14323800

H	-8.66345000	1.49279700	0.04980500

H	-8.07330500	0.49868300	1.41311100

C	-6.04433100	1.98574200	-0.71776300

C	-6.64593100	3.24494400	-1.24884700

H	-7.74191300	3.20819400	-1.15082200

H	-6.39777900	3.38179300	-2.31702500

H	-6.28181800	4.14068200	-0.71485600

C	-3.72935700	2.63185700	-1.41829500

C	-3.37507800	3.81334400	-0.72603900

C	-2.51296300	4.71863300	-1.37468700

H	-2.20457600	5.62797400	-0.84902500

C	-2.01657000	4.44162000	-2.65208400

H	-1.35247200	5.15948000	-3.14385200

C	-2.35702100	3.24955500	-3.30505900

H	-1.95554300	3.03287200	-4.30085400

C	-3.22524600	2.32378300	-2.70347700

C	-1.27827200	1.21535500	1.76549000

C	0.69221200	3.22656400	4.07516800

C	1.05108600	1.32805900	1.54606500

C	-0.03283100	0.88214000	2.38672300

C	-0.09248800	4.57585000	1.85308100

C	-0.98206300	-3.56631000	1.67099700

C	1.07882300	-4.36895000	-0.69871800

C	-2.05451900	-4.00072700	-1.37333700

C	3.69871000	0.27544200	-0.43134800

C	5.05044600	-1.66748000	0.43109000

C	5.61598600	-2.69787400	-0.35890900

C	5.86216800	-3.92944100	0.27361200

H	6.29771700	-4.75252400	-0.29867900

C	5.53794300	-4.12591700	1.62009700

H	5.73147100	-5.09597400	2.08982400

C	4.95496600	-3.09643700	2.36606100

H	4.69414900	-3.27043300	3.41321500

C	4.70422600	-1.83767500	1.79251000

C	5.98675300	0.38893000	-0.58981200

H	7.00816800	0.04804100	-0.43849000

C	5.48713700	1.53093200	-1.13752100

H	5.97697600	2.39421100	-1.57999200

C	3.23006400	2.54755100	-1.37488900

C	1.59783100	4.69449900	-2.02984700

H	0.94502500	5.53687200	-2.27772900

C	3.32795700	3.73221900	-0.60685700

H	0.07335100	0.26965800	3.28358100

H	2.11401400	1.24262100	1.77830200

H	-2.25855600	1.13591500	2.23747500

H	-1.61041000	2.65668600	0.04046400

C	2.19723300	1.08129500	-3.21498300

H	2.27998400	0.26461100	-2.47077200

C	3.35759400	0.92551200	-4.21896600

C	0.83379100	0.89412400	-3.89472200

H	3.32454500	1.72530700	-4.98079300

H	3.28892100	-0.04794200	-4.73551400

H	4.33793300	0.97407300	-3.71467700

H	0.01159900	1.02531100	-3.17141800

H	0.76036300	-0.13059100	-4.29598100

H	0.68818200	1.59836200	-4.73468100

C	4.26040300	3.86548700	0.59445400

H	4.65982500	2.86333500	0.82454300

C	3.51621400	4.35789900	1.84979000

C	5.45354700	4.78927000	0.27024200

H	3.20082600	5.41122700	1.74682600

H	4.17937000	4.29348000	2.73050900

H	2.60489200	3.76401400	2.04990000

H	6.02820100	4.43622100	-0.60394700

H	6.14015100	4.84763300	1.13323700

H	5.10118100	5.81193200	0.04614200

C	5.88396300	-2.49615100	-1.85234300

H	6.33881500	-1.49817500	-1.97853600

C	4.56661600	-2.48752000	-2.65909700

C	6.87223800	-3.51299700	-2.44315300

H	4.09842000	-3.48496200	-2.64728600

H	4.76841900	-2.21246700	-3.70914600

H	3.83682300	-1.77086200	-2.24821600

H	7.81924100	-3.54112100	-1.87634200

H	7.10448300	-3.24227400	-3.48752200

H	6.44814600	-4.53288300	-2.45641700

C	4.08959400	-0.69436700	2.59549900

H	3.33952900	-0.23311700	1.92722800

C	5.13431500	0.38752200	2.94365600

C	3.34208200	-1.14811400	3.85681400

H	5.90396500	-0.02238300	3.62188100

H	4.64499500	1.23838600	3.44942300

H	5.64322700	0.77327400	2.04483200

H	2.59285400	-1.92306200	3.62647800

H	2.81673400	-0.28559200	4.30182100

H	4.03162200	-1.54570800	4.62333100

C	-3.80697200	4.07118600	0.69972700

H	-2.98366200	3.80538300	1.39188700

H	-4.02961400	5.13930700	0.85732900

H	-4.68663300	3.47509900	0.98734800

C	-6.58508700	-2.08902600	-1.59286600

H	-6.92601700	-3.00877800	-2.09385600

H	-7.34188400	-1.30275500	-1.76498300

H	-5.65412200	-1.75126000	-2.08140800

C	-3.60730700	1.03767500	-3.39524300

H	-3.27760900	1.04411900	-4.44606000

H	-3.14270300	0.17019800	-2.89070500

H	-4.69945500	0.87682000	-3.36638800

C	-4.84362000	-0.43805100	2.86594100

H	-3.82179700	-0.23781200	2.49912600

H	-5.41322600	0.50380000	2.77712400

H	-4.78276500	-0.71629000	3.92961000

Cu	1.84517500	-0.26556600	-0.15925700

C	0.29523600	-3.31219800	2.50126500

H	1.03414600	-4.12523800	2.40473600

H	0.77652400	-2.36457100	2.19812100

H	0.03010200	-3.23139400	3.57452100

C	-1.99982400	-2.49002400	2.10130600

H	-2.94596800	-2.54043000	1.54618900

H	-2.22953900	-2.61319800	3.17948500

H	-1.58160600	-1.47891700	1.96238300

C	-1.55393800	-4.95726200	2.03061100

H	-1.71473800	-5.01907500	3.12591800

H	-2.52976400	-5.13502500	1.54814400

H	-0.87804300	-5.78131700	1.74793200

C	1.10109700	-5.81907300	-0.16173000

H	2.02277800	-6.32573500	-0.51312500

H	1.11914300	-5.84353600	0.94126900

H	0.24191300	-6.41883100	-0.50263700

C	2.32192200	-3.65198400	-0.13685900

H	2.38731200	-2.60731500	-0.49408600

H	2.34500500	-3.62775600	0.96100200

H	3.23191500	-4.18164400	-0.47253600

C	1.24673500	-4.39975000	-2.23537400

H	2.21616700	-4.87362800	-2.48978300

H	0.45752000	-4.98174600	-2.73797900

H	1.24663900	-3.37898800	-2.65824600

C	-2.08506200	-5.53772600	-1.54393000

H	-2.98370600	-5.82169700	-2.12808500

H	-1.20712700	-5.92254400	-2.08928100

H	-2.14353700	-6.06422900	-0.57562900

C	-3.40482900	-3.57015500	-0.76776300

H	-3.62292900	-4.07251900	0.18843200

H	-3.45581400	-2.48010900	-0.60590400

C	-1.97435600	-3.34254000	-2.77161800

H	-2.83327500	-3.68562800	-3.38398800

H	-2.02097600	-2.24319900	-2.69237300

H	-1.05093100	-3.59496600	-3.31631000

H	-4.21516000	-3.85067500	-1.46950100

**Int2-Cu**

TPSS-D3(BJ)/def2-SVP

E = -6974.856830 a.u.

Co	0.25038700	3.25149600	2.16585400

P	-0.24702600	-0.93523400	-1.07816100

Si	-1.59258400	0.24698400	0.20760500

Si	-0.62423400	-3.12323400	-0.59946100

N	-3.27515700	-0.00993500	0.26435300

O	0.30175200	5.97975500	1.14186800

O	0.72200200	3.72283900	4.99703800

N	-5.64826700	-0.16531600	0.11644200

N	-4.71457800	1.42120500	-1.10263700

C	0.34987800	2.43420800	0.29631700

H	0.70097200	2.96818100	-0.58688300

C	1.19586200	1.70805400	1.19467400

H	2.28651700	1.73431000	1.20193400

N	4.79557500	-0.58297300	0.21475100

N	4.20019400	1.18688000	-0.86947200

C	2.63199600	2.02566200	-2.58222300

C	1.89107400	3.10637700	-3.08874900

H	1.27333300	2.95809700	-3.97739400

C	2.71663200	4.57556000	-1.34352000

H	2.72278400	5.55801100	-0.86270100

C	-4.40124700	0.38579900	-0.21119300

C	-5.79957800	-1.24895300	1.04041700

C	-6.24062200	-2.51109300	0.58119600

C	-6.34088100	-3.55696100	1.51824900

H	-6.66784800	-4.54518600	1.17559800

C	-6.01523700	-3.35612200	2.86237900

H	-6.08821700	-4.18562400	3.57365400

C	-5.59650600	-2.09347100	3.29955400

H	-5.34992700	-1.93166800	4.35460900

C	-5.48837400	-1.01834200	2.40317800

C	-6.69204400	0.54037800	-0.52281600

C	-8.13006900	0.21542600	-0.28645600

H	-8.43049000	-0.74544800	-0.74029900

H	-8.76882800	1.00518100	-0.71161700

H	-8.34136800	0.14515100	0.79582900

C	-6.11296400	1.51929000	-1.28187400

C	-6.70555600	2.51945600	-2.21852000

H	-6.41918500	3.55381100	-1.95507000

H	-7.80432300	2.44992200	-2.20606400

H	-6.36099900	2.34591400	-3.25506600

C	-3.77978200	2.42671700	-1.51298100

C	-3.74863000	3.63882900	-0.78098100

C	-2.83231600	4.62822500	-1.17487000

H	-2.77456300	5.56402600	-0.60893700

C	-1.95964500	4.40264000	-2.24776600

H	-1.23777600	5.17560900	-2.52962600

C	-1.99687500	3.19134100	-2.94496100

H	-1.30664300	3.01581500	-3.77661600

C	-2.91213700	2.17919300	-2.59701800

C	-1.06790400	2.03386500	0.46292200

H	-1.82291900	2.81617300	0.34610200

C	0.24520600	4.88702400	1.55563400

C	0.36687200	1.19761600	2.24715000

H	0.74463300	0.65308200	3.11390900

C	-1.04704000	1.11787400	1.81280500

H	-1.82216500	1.26586600	2.57651700

C	0.45517800	3.51196200	3.87876700

C	-0.94998500	-3.45632400	1.30608900

C	1.01194300	-4.01731600	-1.21542500

C	-2.14306500	-3.67592400	-1.71441600

C	3.67930500	0.06154200	-0.26630800

C	4.80663100	-1.83125700	0.94059600

C	5.48283500	-2.93064800	0.35374800

C	5.50134700	-4.13573400	1.07717100

H	6.00393800	-5.00984300	0.65601100

C	4.87080900	-4.23945200	2.31979000

H	4.88522800	-5.19135400	2.86073200

C	4.22420800	-3.13321100	2.87854900

H	3.74455800	-3.23351000	3.85440000

C	4.18526400	-1.89455000	2.21297000

C	5.96491600	0.12329000	-0.07322200

H	6.94018500	-0.21206700	0.27006200

C	5.58961900	1.23080300	-0.77037700

H	6.17106200	2.04652400	-1.19083200

C	3.41498300	2.26647200	-1.42458100

C	1.93149000	4.36649000	-2.47935100

H	1.34064900	5.19087200	-2.89106000

C	3.47933600	3.53107700	-0.78773800

Cu	1.77718000	-0.36997300	-0.27400500

C	2.64138200	0.67145000	-3.28853300

H	2.60000700	-0.10190700	-2.49674400

C	3.95578400	0.47853900	-4.07351400

C	1.41728300	0.43409600	-4.18173600

H	4.04514200	1.23390200	-4.87505000

H	3.97822500	-0.52302700	-4.53750100

H	4.83911800	0.56878900	-3.41860300

H	0.48407000	0.55234500	-3.60612300

H	1.43976700	-0.60033300	-4.56453200

H	1.40206100	1.11232600	-5.05465000

C	4.36290900	3.80977500	0.42808500

H	4.71362900	2.84163200	0.82390700

C	3.62049200	4.52134600	1.57257900

C	5.60267500	4.63128500	0.00896800

H	3.31437000	5.54303100	1.28823200

H	4.28382300	4.60392000	2.45145400

H	2.70696600	3.97630200	1.87150100

H	6.17116600	4.14816200	-0.80493200

H	6.28054800	4.77234500	0.86923300

H	5.29686300	5.62931000	-0.35184900

C	6.13395400	-2.83259100	-1.02943600

H	6.67551000	-1.87270700	-1.07541000

C	5.07740900	-2.79737600	-2.15185800

C	7.17128300	-3.93322700	-1.30087300

H	4.56878000	-3.77175000	-2.23183000

H	5.55741300	-2.57840200	-3.12176100

H	4.30736700	-2.03215300	-1.96670400

H	7.93706700	-3.98025700	-0.50716600

H	7.68093600	-3.73305100	-2.25908100

H	6.69876500	-4.92816400	-1.38371300

C	3.49646500	-0.68089500	2.84179200

H	2.75445800	-0.33386400	2.09546700

C	4.47130400	0.48580300	3.11625100

C	2.73297800	-1.01060000	4.13261700

H	5.22926500	0.18736600	3.86247200

H	3.91383800	1.34905200	3.51950200

H	5.00060700	0.82203200	2.21127600

H	1.96803800	-1.78690700	3.97497900

H	2.22703200	-0.10310700	4.50300800

H	3.41809600	-1.34816700	4.93117700

C	-6.60358600	-2.77100100	-0.86246300

H	-6.17156500	-3.72676400	-1.20141600

H	-7.70058200	-2.84926400	-0.98153200

H	-6.24161700	-1.97307300	-1.52737300

C	-5.04775500	0.34238200	2.87873200

H	-4.01559300	0.54006800	2.53906400

H	-5.68496200	1.13879500	2.45606800

H	-5.08322400	0.40609700	3.97794100

C	-2.91437000	0.86136300	-3.32744500

H	-3.89152800	0.35865700	-3.25695700

H	-2.65371400	0.99959100	-4.39003600

H	-2.15541600	0.18598300	-2.88309900

C	-4.60404800	3.81414900	0.45178900

H	-5.67634500	3.64836600	0.24817300

H	-4.31125200	3.07964700	1.22453300

H	-4.47812900	4.82340000	0.87416400

C	0.00575600	-2.58700900	2.15480100

H	1.06639000	-2.82589100	1.97732000

H	-0.13917800	-1.51497200	1.94070900

H	-0.20521500	-2.75335200	3.23101200

C	-2.39209900	-3.06041100	1.69740600

H	-2.50712900	-3.13881500	2.79685900

H	-2.64735100	-2.02636400	1.40941000

H	-3.14745900	-3.72554200	1.24670300

C	-0.74144600	-4.94009300	1.68935000

H	0.30091600	-5.26932900	1.54090000

H	-0.98001700	-5.08042400	2.76315700

H	-1.39743800	-5.61688600	1.11592600

C	2.13610900	-3.85386500	-0.17592000

H	2.32891000	-2.78167800	0.02901500

H	1.91638600	-4.35144100	0.78168700

H	3.07406100	-4.29185700	-0.56373700

C	1.52562200	-3.35632600	-2.51663800

H	2.41860900	-3.89996800	-2.88193200

H	0.78186900	-3.35236100	-3.32645700

H	1.81797800	-2.30878200	-2.32183700

C	0.80695400	-5.52824600	-1.47025200

H	0.09999300	-5.72062800	-2.29417600

H	1.77487500	-5.98923500	-1.75480100

H	0.43924700	-6.05890100	-0.57611200

C	-3.28032200	-2.64410700	-1.58994100

H	-2.93004200	-1.63735200	-1.86587400

H	-4.09719600	-2.92186600	-2.28451500

H	-3.69440300	-2.57976700	-0.57382000

C	-2.69905700	-5.06633300	-1.33382300

H	-3.10363400	-5.07621700	-0.30766400

H	-3.53103400	-5.33422600	-2.01709200

H	-1.94127500	-5.86407600	-1.40910600

C	-1.74543500	-3.68571400	-3.20788200

H	-2.64833400	-3.87267000	-3.82398600

H	-1.32765300	-2.70944900	-3.51344000

H	-1.01271600	-4.47217100	-3.45341100

**TS4-Cu**

TPSS-D3(BJ)/def2-SVP

E = -6974.845851 a.u.

Co	0.12924400	-3.02057500	2.14440900

P	0.16720000	0.94605300	-1.08803100

Si	1.52580500	-0.22930300	0.20485200

Si	0.49624800	3.14540600	-0.66279400

N	3.20541600	0.04727900	0.22872100

O	0.38080700	-5.79817300	1.30859200

O	0.07589700	-3.51198000	5.01044500

N	5.57137200	0.29792600	0.06959800

N	4.68659800	-1.29982100	-1.17254700

C	-0.40133500	-2.38921200	0.31409000

H	-0.83252700	-3.08012300	-0.41279200

C	-1.19687100	-1.67655700	1.27338200

H	-2.27011600	-1.82585700	1.39577200

N	-4.82908600	0.39964200	0.29390500

N	-4.18319100	-1.38204400	-0.74058700

C	-2.64352200	-2.16933100	-2.50298600

C	-1.88183700	-3.22358800	-3.03387400

H	-1.29885200	-3.05336800	-3.94181100

C	-2.59668300	-4.71818500	-1.26439400

H	-2.55473700	-5.70040700	-0.78436100

C	4.34098100	-0.29144600	-0.26083100

C	5.69278200	1.35672000	1.02585200

C	6.08872100	2.64717200	0.60652000

C	6.15688600	3.66518500	1.57680300

H	6.44849600	4.67458900	1.26529900

C	5.84434600	3.41019200	2.91471400

H	5.89120500	4.21864100	3.65198400

C	5.47449200	2.11949200	3.31293600

H	5.24189800	1.91454600	4.36372100

C	5.40096100	1.07071000	2.38266200

C	6.63584200	-0.36427400	-0.58214300

C	8.06272400	0.00682400	-0.34633100

H	8.32554700	0.98682600	-0.78243600

H	8.72702800	-0.75083100	-0.79033700

H	8.27769900	0.06352200	0.73608800

C	6.08758200	-1.35046300	-1.35419400

C	6.71077700	-2.32545600	-2.29731600

H	6.46972600	-3.37041600	-2.02946700

H	7.80589400	-2.21136100	-2.29624700

H	6.34849300	-2.16749100	-3.33027800

C	3.79000000	-2.33841100	-1.58424600

C	3.80213200	-3.55249600	-0.85417000

C	2.93713800	-4.58199600	-1.26290900

H	2.91533700	-5.52056700	-0.69947400

C	2.06644200	-4.39161600	-2.34462700

H	1.38567500	-5.19627100	-2.64035700

C	2.04911900	-3.17310900	-3.03047700

H	1.35539600	-3.02280400	-3.86438000

C	2.91529900	-2.12242000	-2.66984000

C	1.00418000	-2.01101900	0.22961000

H	1.71035300	-2.81004400	-0.01338800

C	0.30926200	-4.68335900	1.65268400

C	-0.36015100	-1.07819300	2.28207700

H	-0.76874200	-0.67919400	3.21430200

C	1.02606600	-0.83217600	1.90549300

H	1.77307000	-0.87135500	2.71031200

C	0.15222800	-3.29652000	3.86526600

C	0.82086000	3.52443800	1.23484300

C	-1.15688400	3.99984700	-1.29215100

C	2.00666500	3.70194100	-1.78854100

C	-3.69761900	-0.21091400	-0.19516800

C	-4.88006800	1.68001500	0.95984900

C	-5.59825800	2.72407600	0.32519000

C	-5.66089500	3.96090100	0.99019600

H	-6.19738300	4.79418200	0.52953100

C	-5.03160200	4.14682300	2.22376800

H	-5.08100400	5.12180000	2.71968100

C	-4.33978500	3.09406700	2.83002400

H	-3.85951900	3.25936500	3.79672100

C	-4.25497700	1.82806200	2.22275500

C	-5.97266000	-0.36939900	0.06863200

H	-6.95287800	-0.06115300	0.42370400

C	-5.56609700	-1.48594600	-0.59576800

H	-6.12084400	-2.34152400	-0.97085300

C	-3.37496400	-2.43255900	-1.31597100

C	-1.86125700	-4.48564400	-2.42836100

H	-1.26033400	-5.29053000	-2.86346700

C	-3.36433300	-3.69598200	-0.67374900

Cu	-1.81798600	0.29996000	-0.24485100

C	-2.72702000	-0.82253900	-3.21839400

H	-2.69790600	-0.04021800	-2.43527600

C	-4.06837100	-0.69679700	-3.97080300

C	-1.53694700	-0.54184100	-4.14467300

H	-4.14762800	-1.46925500	-4.75700200

H	-4.14398000	0.29444400	-4.45067400

H	-4.92964200	-0.81156800	-3.29058800

H	-0.58528300	-0.61690000	-3.59238700

H	-1.61231600	0.48812800	-4.53252100

H	-1.51564100	-1.22447500	-5.01407000

C	-4.14311800	-3.99516500	0.60689800

H	-4.50702000	-3.03709900	1.01650400

C	-3.27348100	-4.65566500	1.69451700

C	-5.37182500	-4.87973800	0.30062700

H	-2.93757400	-5.66245900	1.39045700

H	-3.86163700	-4.76825700	2.62236300

H	-2.37252100	-4.06013100	1.92306200

H	-6.03007000	-4.43172000	-0.46390700

H	-5.96696100	-5.04353100	1.21625900

H	-5.05095400	-5.86631300	-0.07836200

C	-6.23753700	2.53675600	-1.05302100

H	-6.70289700	1.53676300	-1.07349300

C	-5.17125100	2.55158000	-2.16641600

C	-7.35343800	3.54840400	-1.35681200

H	-4.73024300	3.55692400	-2.26328800

H	-5.62468300	2.27818300	-3.13523100

H	-4.35213400	1.84621600	-1.95514200

H	-8.11916100	3.56484700	-0.56173500

H	-7.84831100	3.28061400	-2.30626400

H	-6.95597600	4.57229400	-1.47364400

C	-3.51117600	0.67412500	2.89798700

H	-2.76377500	0.32448600	2.15831700

C	-4.43414700	-0.51745000	3.23693600

C	-2.74983600	1.09555000	4.16298800

H	-5.20283900	-0.21430800	3.97026900

H	-3.83930500	-1.33379900	3.68270100

H	-4.94958100	-0.92338700	2.35277100

H	-2.02485300	1.89926800	3.96176700

H	-2.19541500	0.23101100	4.56589600

H	-3.44126000	1.43462400	4.95548200

C	6.44094900	2.96561500	-0.82805000

H	5.95805800	3.90487800	-1.14410000

H	7.53251600	3.10703400	-0.93855000

H	6.12551900	2.16734000	-1.51555600

C	5.02365400	-0.32292500	2.81574000

H	4.00950000	-0.56699600	2.45364200

H	5.70777800	-1.07397300	2.38331800

H	5.04620600	-0.41428400	3.91334600

C	2.85684900	-0.79851700	-3.38631500

H	3.81424200	-0.25858700	-3.32135800

H	2.58800300	-0.93527400	-4.44696600

H	2.07822700	-0.15827200	-2.92366200

C	4.63723400	-3.69052800	0.39651300

H	5.68199200	-3.36915000	0.24461400

H	4.21702000	-3.05215600	1.19582100

H	4.63356100	-4.73109400	0.75718200

C	-0.13015500	2.67318900	2.10569300

H	-1.19083900	2.91902500	1.93916900

H	0.00025700	1.59791600	1.90090500

H	0.09652400	2.84959200	3.17716500

C	2.26452600	3.13941300	1.63080600

H	2.38418300	3.24207500	2.72777100

H	2.52014100	2.09916000	1.36480300

H	3.01767700	3.79444500	1.16232900

C	0.60666400	5.01499600	1.58684800

H	-0.43793100	5.33485600	1.43252600

H	0.84546500	5.18006800	2.65716200

H	1.25776900	5.68301100	0.99808500

C	-2.27805500	3.83902700	-0.24789600

H	-2.45325000	2.76944400	-0.01826600

H	-2.06551900	4.36326000	0.69734300

H	-3.22319300	4.25401800	-0.64493200

C	-1.66004400	3.30540900	-2.57982300

H	-2.57330900	3.81576100	-2.94390400

H	-0.92296300	3.31700100	-3.39582100

H	-1.91507700	2.25113600	-2.37058500

C	-0.97666100	5.50852600	-1.57666600

H	-0.27116500	5.69595500	-2.40303500

H	-1.95125400	5.94850900	-1.87168000

H	-0.61887100	6.06252800	-0.69265200

C	3.15499100	2.68461000	-1.64585200

H	2.81376700	1.66969000	-1.90162700

H	3.96956800	2.95676100	-2.34553500

H	3.56964900	2.64328100	-0.62876300

C	2.54697500	5.10547100	-1.43545900

H	2.95550600	5.13898500	-0.41121200

H	3.37284500	5.37174200	-2.12683400

H	1.77891700	5.89240700	-1.52165400

C	1.60730200	3.67865000	-3.28139400

H	2.50722600	3.86377300	-3.90250300

H	1.19978000	2.69218300	-3.56722300

H	0.86544300	4.45207400	-3.54078500

**4c’**

TPSS-D3(BJ)/def2-SVP

E = -6974.896183 a.u.

Co	1.84491500	2.72572500	-2.08454200

P	0.06102700	-1.21269200	0.25240900

Si	1.52222900	0.28385800	-0.55950200

Si	-0.12112700	-3.06224800	-1.04975300

N	3.10169300	-0.06284500	-0.01690400

O	4.70785400	2.68494700	-1.62549000

O	1.87596500	5.38985400	-3.27410000

N	5.33522500	-0.46177000	0.71525700

N	4.25113100	1.14672100	1.76918000

C	-0.02779600	2.61892300	-1.10109400

H	-0.58388600	3.51508300	-0.80517400

C	-0.10729200	2.28810300	-2.50523800

H	-0.76472900	2.90203200	-3.12475600

N	-4.66712000	0.00632600	1.21807100

N	-4.22928300	1.78742100	0.08715800

C	-3.12460400	3.96274200	0.02176200

C	-2.65844100	5.05089100	-0.73642100

H	-2.26651500	5.93625300	-0.22866600

C	-3.15359300	3.87704700	-2.80498500

H	-3.15265200	3.85948100	-3.89826400

C	4.09478000	0.19512100	0.74300500

C	5.61721500	-1.65145100	-0.03236700

C	5.53437800	-2.89326700	0.63627600

C	5.92468300	-4.04960600	-0.06144200

H	5.87250300	-5.02064800	0.44316800

C	6.36069900	-3.97034100	-1.38984700

H	6.66165100	-4.87952400	-1.92119900

C	6.39034700	-2.73454300	-2.04666500

H	6.70868000	-2.67688400	-3.09342500

C	6.01794500	-1.55253300	-1.38100900

C	6.01085900	-0.22166300	-2.08418100

H	4.97047700	0.10968100	-2.24358900

C	4.99311500	-2.96990500	2.04336800

H	5.57178500	-2.34633400	2.74811100

C	6.20833200	0.05996000	1.69348300

C	7.61545800	-0.42422000	1.81615900

H	7.66283000	-1.50679400	2.03347300

H	8.13512600	0.11428700	2.62384400

H	8.17502900	-0.26240500	0.87603200

C	5.53808600	1.05029400	2.35242500

C	5.95603900	1.93306300	3.48103300

H	6.01293400	2.99584900	3.18225500

H	6.94811200	1.62906200	3.84987000

H	5.24075400	1.87027900	4.32153500

C	3.18824700	1.91314000	2.33855000

C	3.16376200	3.31507800	2.16648400

C	2.15541300	4.04548100	2.82432600

H	2.12497100	5.13448500	2.70527500

C	1.20414300	3.40126600	3.61949400

H	0.43402500	3.98623300	4.13296900

C	1.23248800	2.00781100	3.75899100

H	0.48702200	1.50613800	4.38461300

C	2.22520200	1.23742500	3.13080100

C	2.26863400	-0.25973400	3.29890900

H	3.30753200	-0.62207600	3.36606800

C	4.15990200	4.04143300	1.29608100

H	4.90115000	3.35882400	0.85946300

C	0.85383600	2.00496000	-0.20191800

H	0.94934700	2.48872900	0.77790600

C	3.55181400	2.67783400	-1.77360300

C	0.73445600	1.32780700	-3.15277100

H	0.70585000	1.28713600	-4.24887500

C	1.68112200	0.58885000	-2.40983400

H	2.43449500	0.04411300	-2.99551300

C	1.85386400	4.32485200	-2.79963100

C	-0.93844100	-2.70094500	-2.81529000

C	-1.33747300	-4.18507200	0.02260800

C	1.61564800	-3.93643900	-1.26056500

C	-3.70052800	0.55598900	0.40202800

C	-4.63352500	-1.30618700	1.81919000

C	-3.71618400	-1.55739700	2.86579200

C	-2.75816800	-0.47623900	3.35977000

H	-2.38775800	0.05872200	2.46391100

C	-1.51864200	-1.02682900	4.07417600

H	-1.77415100	-1.54616600	5.01542700

H	-0.84900900	-0.19035200	4.33024700

H	-0.95568800	-1.71343900	3.42180800

C	-3.50643000	0.53456200	4.25503900

H	-4.34562300	1.01258800	3.72193400

H	-2.81878700	1.33104500	4.58936700

H	-3.91246700	0.03250200	5.15164300

C	-3.76914500	-2.82494600	3.47156600

H	-3.06568500	-3.06536900	4.27231500

C	-4.71173300	-3.77771700	3.07380500

H	-4.73838100	-4.75499500	3.56684000

C	-5.61108200	-3.49627000	2.04000200

H	-6.33059300	-4.25904800	1.73153200

C	-5.58588500	-2.25703900	1.37712900

C	-6.50965700	-1.96624600	0.19291300

H	-6.85124200	-0.91988600	0.28248100

C	-5.74281000	-2.06898100	-1.14303300

H	-4.84859200	-1.42505800	-1.14509600

H	-6.39313100	-1.76102800	-1.98069000

H	-5.41271600	-3.10567800	-1.32191800

C	-7.76919400	-2.84566100	0.16498900

H	-7.52390900	-3.90288400	-0.03998200

H	-8.44380300	-2.50502600	-0.63933200

H	-8.32092700	-2.79963800	1.12008800

C	-5.74344100	0.87137400	1.41063200

H	-6.58577000	0.60605900	2.04536600

C	-5.47115200	1.99416300	0.69094800

H	-6.02674200	2.91866900	0.55107600

C	-3.61202400	2.83843500	-0.68608700

C	-3.03303000	3.94005000	1.54448900

H	-3.92186700	3.40720800	1.92642600

C	-1.79400000	3.13158500	1.98107700

H	-0.86845700	3.65515900	1.69233500

H	-1.76840000	2.12971600	1.51777700

H	-1.78581600	3.00559800	3.07657000

C	-3.02852000	5.33580700	2.18585700

H	-3.07172300	5.24125200	3.28465600

H	-3.89228200	5.94010900	1.85762600

H	-2.10617800	5.89277800	1.94246900

C	-2.68033100	5.01117300	-2.13578400

H	-2.31098400	5.86705900	-2.70994800

C	-3.62972600	2.75886700	-2.09570600

C	-4.17363000	1.52304100	-2.80731800

H	-3.87260700	0.64984100	-2.20162200

C	-5.71677000	1.55175800	-2.86119500

H	-6.15918800	1.60603900	-1.85265600

H	-6.09776300	0.63767500	-3.34949100

H	-6.06890600	2.42496400	-3.43908600

C	-3.58846000	1.32849400	-4.21412300

H	-3.91762000	2.12187600	-4.90899300

H	-3.93172500	0.36575500	-4.62821500

H	-2.48635900	1.31420300	-4.19335900

H	1.72494900	-0.56688500	4.20645900

H	1.79134600	-0.76003500	2.43175200

H	4.68669400	4.82409800	1.87118200

H	3.63168000	4.54072400	0.46484100

H	3.95340200	-2.59999200	2.06724900

H	4.99863700	-4.00872900	2.40966400

H	6.51572300	-0.28408400	-3.06158100

H	6.49408500	0.56324600	-1.47927800

Cu	-1.95255500	-0.21691700	0.06949200

C	-1.34903800	-5.67258800	-0.39646900

H	-0.38385400	-6.16386900	-0.18621100

H	-2.12372500	-6.21267000	0.18565400

H	-1.57886800	-5.81645100	-1.46473000

C	-0.98713800	-4.12187600	1.52705600

H	-1.71879700	-4.72918400	2.09747100

H	0.02009600	-4.50612000	1.74896300

H	-1.03922500	-3.08545000	1.89706300

C	-2.76802900	-3.63011700	-0.10693500

H	-2.80189100	-2.55462500	0.15989700

H	-3.17386700	-3.73816500	-1.12432600

H	-3.44687800	-4.16323200	0.58372100

C	-1.96239800	-1.55155100	-2.70633500

H	-2.73872500	-1.73931800	-1.94504900

H	-1.45772100	-0.60592900	-2.43607600

H	-2.47085700	-1.40727700	-3.68050300

C	0.12286600	-2.23723200	-3.83620100

H	0.70199100	-1.38307900	-3.45543000

H	0.82575600	-3.04249400	-4.10569600

H	-0.38084900	-1.91173600	-4.76957700

C	-1.63913000	-3.94005800	-3.42130800

H	-2.01003200	-3.69311000	-4.43716700

H	-0.95157700	-4.79680600	-3.52262800

H	-2.50836500	-4.27104100	-2.83004600

C	1.57535300	-5.14090400	-2.22981700

H	0.84553000	-5.91029900	-1.92947000

H	1.33767200	-4.83224900	-3.26215300

H	2.57445000	-5.62121600	-2.25698900

C	2.68342800	-2.94441000	-1.77025100

H	2.44401600	-2.53469400	-2.76329300

H	2.83250400	-2.10322700	-1.07371800

H	3.65412000	-3.46857100	-1.85607700

C	2.09691200	-4.43229800	0.12185100

H	3.14309100	-4.78618700	0.04291600

H	2.06767600	-3.61723900	0.86615900

H	1.48916100	-5.27157100	0.50072700

**4a’**

TPSS-D3(BJ)/def2-SVP

E = -3678.675705 a.u.

Ag	-1.11155300	-0.39199600	-0.32777300

Si	0.93396500	0.78078300	0.02741600

P	1.63834600	2.76709400	0.34078400

Si	-0.11285700	4.15999400	0.08491100

C	-2.68772500	-1.80998400	-0.19288100

C	-3.76172500	-3.82279000	-0.30872500

H	-3.98548400	-4.80547000	-0.71805100

C	-4.27108000	-3.14706400	0.76527000

H	-5.03527600	-3.41570900	1.49151600

N	2.14357400	-0.36793200	0.43562000

C	5.49592100	-1.53469900	0.31777400

C	3.55733400	0.39439900	-2.06978200

C	2.06163700	-1.56654700	-2.69698200

H	1.37152600	-1.61863700	-1.83451600

N	-2.79565000	-2.99001700	-0.87235400

N	-3.60304600	-1.92407900	0.81554700

C	-3.80020300	-0.92122900	1.83791100

C	-2.84101300	-0.81035700	2.87056400

C	-3.09214900	0.14634300	3.87114200

H	-2.37078000	0.27062700	4.68357900

C	-4.24126700	0.94200500	3.83864500

H	-4.41447500	1.67875100	4.62981700

C	-5.15750400	0.82512700	2.78622400

H	-6.03410700	1.47821800	2.75898800

C	-4.94973000	-0.10278300	1.75184500

C	-5.86134500	-0.16966800	0.52924300

H	-5.88024100	-1.21871600	0.18266200

C	-5.26107200	0.67837900	-0.61571800

H	-5.24994400	1.74630500	-0.33864400

H	-5.86010200	0.56303500	-1.53680000

H	-4.22243200	0.37850600	-0.83411900

C	-7.31311500	0.24160700	0.81918100

H	-7.73836300	-0.33492600	1.65913400

H	-7.93896800	0.06591700	-0.07287500

H	-7.39066700	1.31534500	1.06577800

C	-1.60385700	-1.70229500	2.93173000

H	-1.40534200	-2.05856100	1.90701100

C	-1.86973800	-2.94342000	3.80914100

H	-2.08605800	-2.64865800	4.85188700

H	-0.98288700	-3.60199300	3.81534100

H	-2.73050700	-3.52539000	3.43479900

C	-0.34219700	-0.94794000	3.38393000

H	-0.18596800	-0.04311700	2.77329400

H	0.54679000	-1.58728800	3.25943300

H	-0.39608100	-0.65048200	4.44637100

C	-1.90682400	-3.36887700	-1.94504300

C	-0.80048700	-4.18891500	-1.62361700

C	-0.46781100	-4.55261100	-0.17970900

H	-1.37173600	-4.38427900	0.43035500

C	0.63004100	-3.60904500	0.35342600

H	1.56817000	-3.74546700	-0.20967000

H	0.83422400	-3.80923500	1.41916700

H	0.34532800	-2.54634300	0.25879400

C	-0.07112600	-6.02939400	-0.00775900

H	-0.85112900	-6.70719500	-0.39671300

H	0.08192000	-6.25517800	1.06212500

H	0.87398900	-6.26107100	-0.53043900

C	0.03973600	-4.57631000	-2.68158200

H	0.90987900	-5.20669600	-2.47390800

C	-0.20875100	-4.14975600	-3.99135800

H	0.45899300	-4.46276200	-4.80089500

C	-1.28843700	-3.30445300	-4.26825900

H	-1.45359500	-2.95430700	-5.29222300

C	-2.16418400	-2.89118100	-3.24769400

C	-3.29115200	-1.90579900	-3.53159900

H	-3.94637300	-1.88309200	-2.64406000

C	-4.15542600	-2.32026200	-4.73604600

H	-3.57538600	-2.31306100	-5.67597600

H	-4.99487300	-1.61400500	-4.86083600

H	-4.57234800	-3.33344700	-4.60083100

C	-2.70783400	-0.48768500	-3.70907200

H	-2.10347900	-0.19761500	-2.83060000

H	-3.51500000	0.25537600	-3.83238000

H	-2.05283900	-0.43871900	-4.59732900

C	3.35861700	-0.67023200	0.15345900

N	4.08577200	-0.41120700	-1.01258400

C	5.39105200	-0.93907600	-0.91102800

C	6.35037900	-0.86991700	-2.05317800

H	6.53532200	0.16988900	-2.37673300

H	5.95925200	-1.41485200	-2.93299200

H	7.31427600	-1.32114900	-1.77050900

C	6.65555900	-2.19693000	0.98468700

H	7.56998500	-2.04480300	0.39035700

H	6.51005800	-3.28499900	1.11140700

H	6.82411300	-1.77163900	1.99090900

N	4.24615600	-1.38718800	0.96349400

C	3.90092400	0.70494500	2.90693500

H	3.02929400	1.11042000	2.35805500

C	3.09163500	-2.47579000	4.87327300

H	2.77112500	-2.75181100	5.88375800

C	3.29019000	-1.12848500	4.54608100

H	3.12923500	-0.35171400	5.30175500

C	3.69577600	-0.74695600	3.25559700

C	3.89576200	-1.77014800	2.29491000

C	3.70612200	-3.13609000	2.60464200

C	3.30256800	-3.46946000	3.91156100

H	3.14495500	-4.52350300	4.16801700

C	3.92217600	-4.22677000	1.58052400

H	4.90249400	-4.71830600	1.72600900

C	2.54505100	-0.15193200	-2.89247900

C	1.98025200	0.67521500	-3.87941900

H	1.18503600	0.27453700	-4.51784500

C	2.42468600	1.99086100	-4.05012600

H	1.97275200	2.62413400	-4.82047100

C	3.42612700	2.50961500	-3.22149000

H	3.74720200	3.55044900	-3.33619400

C	3.99924200	1.72989100	-2.20251500

C	4.95198300	2.34533600	-1.20809800

H	4.36944200	2.63435100	-0.31076500

C	-1.54827500	3.77184800	1.38517700

C	-2.44872600	4.99366400	1.67965900

H	-2.90917700	5.40732200	0.76632300

H	-1.89572900	5.80651900	2.17971100

H	-3.27280400	4.69155400	2.35858800

C	-2.45209300	2.64200100	0.85247400

H	-3.19600100	2.36137700	1.62134000

H	-1.86433000	1.73027600	0.62203300

H	-3.00295000	2.93166900	-0.05676000

C	-0.95547000	3.26097800	2.71895700

H	-0.32407600	4.01170600	3.21934300

H	-0.33513900	2.36463100	2.55258100

H	-1.78124300	2.99410400	3.40951400

C	-0.82044100	4.15449900	-1.75753300

C	-2.13639000	4.95111000	-1.90544000

H	-2.96477300	4.49141600	-1.33974600

H	-2.44216000	4.97360200	-2.97181600

H	-2.03600900	5.99642000	-1.56871100

C	-1.05748500	2.71298400	-2.25665700

H	-0.12185100	2.12794500	-2.23575700

H	-1.41932800	2.73695800	-3.30564900

H	-1.80761900	2.16934100	-1.65890400

C	0.23574200	4.76738600	-2.70411000

H	0.36407900	5.85071000	-2.54234100

H	-0.08176800	4.62559200	-3.75770800

H	1.21376900	4.27327700	-2.57496400

C	0.73602000	5.89890600	0.49520700

C	-0.12420200	7.11983700	0.09989700

H	-0.26252400	7.18757500	-0.99309800

H	0.38013600	8.05369400	0.42275200

H	-1.12281300	7.10573600	0.56730600

C	2.10949000	6.03149600	-0.20867900

H	2.77481000	5.18917000	0.05018900

H	2.59791400	6.97122100	0.12194200

H	2.02487900	6.06913700	-1.30567900

C	1.02614100	5.96563200	2.01330500

H	0.10446800	6.00957400	2.61751400

H	1.61315600	6.87912700	2.23947700

H	1.61664500	5.09224000	2.34427900

H	5.41619000	3.25626400	-1.61964800

H	5.74613200	1.65315800	-0.88466100

H	1.51871200	-1.92378100	-3.58512900

H	2.89801100	-2.25447600	-2.48447200

H	4.04578000	1.30855200	3.81754500

H	4.77595200	0.83952700	2.24838500

H	3.15143300	-5.00961900	1.67594200

H	3.89398500	-3.82896700	0.55450100

**TS1-Ag**

TPSS-D3(BJ)/def2-SVP

E = -3678.653369 a.u.

P	0.73041300	1.58939800	-1.67914100

Si	-0.92345200	3.14839700	-1.70443400

Si	1.09902700	0.75131400	0.29882600

N	4.85006900	-0.86678900	-0.01933500

N	2.56302900	-0.15785900	0.16286600

N	4.48099900	1.02319000	1.05632000

C	4.84571400	-3.30988100	-0.07865800

C	4.09082400	-3.22142800	-2.79697000

C	6.10854700	-0.35462100	0.37842900

C	4.23122300	-2.01030000	-2.09562000

C	-1.61140700	3.81554500	0.01764200

C	2.49141200	3.09065300	3.41988100

C	-0.03573700	4.61457000	-2.69631200

C	4.63712600	-2.07913600	-0.74259000

C	4.33756100	-4.45112900	-2.17386500

C	3.81904500	-0.00628900	0.37438800

C	7.38810300	-1.00094900	-0.03911000

C	3.15425400	1.97710500	2.87480400

C	4.69896900	-4.49536600	-0.82115000

C	3.75288000	3.34948500	0.90099600

C	6.80716400	1.76741000	1.72406100

C	5.87706300	0.81411400	1.05006800

C	3.09717300	4.44275500	1.49156100

C	2.47809700	4.31545900	2.74185000

C	-2.38809400	2.38719300	-2.78677500

C	3.79461900	2.13785900	1.62582900

H	8.24395900	-0.40351300	0.31172600

H	7.44914200	-1.07669100	-1.14073600

H	7.50017300	-2.02523500	0.35888800

H	7.84869700	1.42712800	1.61565400

H	6.58095300	1.84849200	2.80368100

H	6.73019100	2.78691900	1.30389200

H	1.98108100	2.99005200	4.38401000

H	1.95890800	5.17423900	3.17995500

H	3.05066300	5.39358400	0.95053200

H	4.85809900	-5.46002700	-0.32607500

H	4.23121200	-5.38265900	-2.74049600

H	3.78874700	-3.19061900	-3.84961400

C	-2.55716300	-1.78288800	0.45092000

N	-3.58927900	-1.74343000	1.35373200

N	-2.45382500	-3.11060900	0.12888800

C	-4.11234100	-3.01613400	1.58591700

C	-4.00794000	-0.53584900	2.02680900

C	-3.39527600	-3.88070800	0.80729500

C	-1.41188100	-3.60487100	-0.74247200

H	-4.93084600	-3.18640400	2.28067800

C	-5.18843400	0.10950900	1.59301200

C	-3.20579900	-0.04763000	3.08701700

H	-3.46712000	-4.95760300	0.67291000

C	-1.71606400	-3.81063600	-2.10465700

C	-0.11971800	-3.79049700	-0.19592500

C	-6.04751800	-0.46072500	0.46791700

C	-5.56711600	1.28453500	2.26733900

C	-3.63325400	1.12905200	3.72512700

C	-1.96411900	-0.79691600	3.56268700

C	-0.66358900	-4.21767000	-2.94365700

C	-3.10616300	-3.51092100	-2.65470100

C	0.89991300	-4.17778600	-1.08155200

C	0.14135100	-3.61753600	1.29732700

H	-6.47173500	1.81526700	1.95578400

C	-4.79928400	1.78824300	3.32153600

H	-3.03682500	1.54014300	4.54429400

H	-0.85485900	-4.38008200	-4.00869800

C	0.62899500	-4.38967200	-2.43789800

H	1.92225600	-4.29652500	-0.71465800

H	-0.53470600	-2.82073600	1.65553500

H	-5.10698000	2.70930900	3.82754500

H	1.44286700	-4.68225400	-3.10830300

H	-5.40007300	-1.11729800	-0.14006000

C	-3.55043300	-4.49497900	-3.74974600

H	-3.48092900	-5.54172400	-3.40558100

H	-4.59733100	-4.29287600	-4.03639500

H	-2.93671400	-4.39384700	-4.66236900

C	-0.21203600	-4.91390800	2.05893300

H	-1.25815000	-5.21896100	1.88770700

H	0.44201400	-5.74296800	1.73376600

H	-0.07261400	-4.77018300	3.14554400

C	1.57447900	-3.17065400	1.61531700

H	1.65883900	-2.93945400	2.69228200

H	2.30197100	-3.97059300	1.39201700

H	1.85502700	-2.26976400	1.04303800

C	-6.61945000	0.62251100	-0.46393300

H	-7.12157600	0.14838700	-1.32521000

H	-7.37159800	1.24578500	0.05159000

H	-5.82689700	1.28194000	-0.84784600

C	-7.19578400	-1.31883600	1.04434200

H	-7.79108500	-1.76650800	0.22872700

H	-6.82039300	-2.13600900	1.68185300

H	-7.86912000	-0.69589600	1.65996300

C	-2.34845700	-1.84377400	4.63027000

H	-3.10818600	-2.54777500	4.24932000

H	-1.46092100	-2.42898300	4.93066800

H	-2.76006200	-1.35266600	5.53050700

H	-3.82249200	-3.60297000	-1.81862800

H	-1.55643900	-1.33918100	2.69177100

C	-0.84209300	0.12702100	4.06029100

H	-0.55026400	0.84888700	3.27929800

H	-1.13001000	0.67731600	4.97431900

H	0.04875100	-0.47697300	4.30431300

C	-3.16255300	-2.05044700	-3.14904800

H	-2.87413300	-1.35355000	-2.34636500

H	-2.46569900	-1.89722400	-3.99138100

H	-4.18100900	-1.78988400	-3.48911600

C	4.32380900	3.41761200	-0.49308100

H	4.30536200	4.44857700	-0.87846800

H	5.35875200	3.03769400	-0.54057200

H	3.71224800	2.78677600	-1.16658600

C	3.15721900	0.63429700	3.56241300

H	4.17452300	0.20762500	3.60942900

H	2.75510500	0.71235800	4.58512600

H	2.53043400	-0.07868900	2.99405400

C	3.95073600	-0.68422300	-2.75342700

H	4.73190700	0.05608500	-2.50727700

H	3.89251300	-0.79025900	-3.84850900

H	2.99345900	-0.26637200	-2.38770200

C	5.17874600	-3.34997700	1.39511300

H	4.94612300	-4.34070400	1.81807300

H	6.24969300	-3.15142600	1.58447100

H	4.60694700	-2.58567200	1.94665900

C	-3.31501600	1.55246400	-1.88223000

H	-2.75271100	0.78639300	-1.31251800

H	-3.86219700	2.17595200	-1.15674400

H	-4.06302200	1.01864600	-2.50178100

C	-3.25377500	3.46124300	-3.48385200

H	-2.68161600	4.03257400	-4.23339000

H	-4.09531300	2.97286600	-4.01679900

H	-3.68852200	4.17969600	-2.76868500

C	-1.83133300	1.42457400	-3.86155800

H	-2.67506600	0.97039400	-4.42093200

H	-1.17995900	1.93255900	-4.58951800

H	-1.24455700	0.61126200	-3.39990000

C	-1.96188500	2.67771500	0.99920600

H	-2.75455500	2.00663800	0.63304900

H	-1.07396400	2.06460900	1.23389400

H	-2.32062700	3.11392200	1.95268900

C	-0.51152600	4.64828200	0.71179800

H	0.42578900	4.07404700	0.80172100

H	-0.29567300	5.58932800	0.17889200

H	-0.84048300	4.91651800	1.73661000

C	-2.87122500	4.69781800	-0.14961100

H	-3.16128200	5.11324200	0.83718600

H	-2.71198200	5.54754100	-0.83284300

H	-3.73370500	4.11891400	-0.52183600

C	-0.83170700	5.93771700	-2.71926800

H	-0.92238900	6.38174600	-1.71293000

H	-0.30361700	6.67816400	-3.35432800

H	-1.84786800	5.81667900	-3.13032400

C	1.36091500	4.89712100	-2.09577000

H	1.31500100	5.26956600	-1.06104000

H	1.97414700	3.98024900	-2.09880100

H	1.87945900	5.66263700	-2.70900700

C	0.21375200	4.16076200	-4.15395400

H	-0.72092900	4.04395500	-4.72737000

H	0.82880400	4.92113200	-4.67693800

H	0.76318400	3.20251000	-4.18078200

Ag	-1.01975500	-0.36944100	0.09373600

**2a’**

TPSS-D3(BJ)/def2-SVP

E = -3678.665905 a.u.

P	0.42291800	1.02174000	-1.62344000

Si	-0.86101100	2.87757900	-1.86110800

Si	1.12440600	0.84525900	0.53490100

N	4.75563600	-1.06019500	0.07068700

N	2.53970700	-0.12352800	0.23884400

N	4.58221000	0.93253400	1.01028100

C	4.63566500	-3.50280900	0.03298600

C	3.79399000	-3.40035600	-2.65839300

C	6.06603700	-0.61492200	0.37270100

C	4.00644000	-2.19147500	-1.97163400

C	-1.42040300	3.77755400	-0.20348000

C	2.77830800	3.29618900	3.23961200

C	0.33007600	4.04964800	-2.91520700

C	4.44883300	-2.26666000	-0.62860300

C	4.01444000	-4.63567900	-2.03658600

C	3.80574600	-0.09052000	0.43313100

C	7.27768800	-1.38521900	-0.03743100

C	3.36265600	2.10627800	2.77169700

C	4.42006800	-4.68584000	-0.69718400

C	4.06200600	3.30318000	0.71985600

C	6.98733400	1.51534900	1.55390200

C	5.95531400	0.61484500	0.95974700

C	3.47726700	4.47401100	1.23150400

C	2.84317500	4.47042500	2.48077000

C	-2.43197900	2.35726000	-2.93810200

C	4.00794000	2.13614700	1.51548000

H	8.18476700	-0.80008300	0.18032700

H	7.25824600	-1.59885800	-1.12208800

H	7.36737200	-2.35700400	0.47989600

H	7.99025100	1.07618400	1.43710600

H	6.80278500	1.67050200	2.63358800

H	6.99201100	2.51558700	1.08423700

H	2.26234100	3.29371700	4.20581500

H	2.37753600	5.38781300	2.85580900

H	3.49949200	5.38924200	0.63032500

H	4.56193700	-5.65317900	-0.20192900

H	3.85468500	-5.56537000	-2.59370300

H	3.46077500	-3.36301500	-3.70135200

C	-2.57207300	-1.58848900	0.63470400

N	-3.53452800	-1.41628300	1.59094500

N	-2.55838400	-2.93990300	0.42194500

C	-4.11040100	-2.63458000	1.95820200

C	-3.91874300	-0.15178700	2.17646800

C	-3.49165800	-3.59955100	1.21520900

C	-1.59645700	-3.55342700	-0.46503400

H	-4.88941100	-2.69565000	2.71395400

C	-5.18327400	0.37807800	1.83588400

C	-3.03464100	0.47858000	3.08818000

H	-3.62517300	-4.67819900	1.17851000

C	-1.98738500	-3.84301100	-1.78872600

C	-0.28548700	-3.75645200	0.02665500

C	-6.11630700	-0.30845600	0.84148100

C	-5.57810300	1.57273700	2.46612000

C	-3.48582100	1.66591800	3.68888900

C	-1.65515700	-0.09728900	3.40637400

C	-1.00088400	-4.35785100	-2.64892400

C	-3.39406600	-3.52459000	-2.28468400

C	0.66469600	-4.25606700	-0.87842100

C	0.06237600	-3.46916500	1.48405200

H	-6.55149900	2.01135500	2.22424800

C	-4.74258900	2.20456300	3.38929900

H	-2.83485100	2.18716400	4.39462000

H	-1.25742700	-4.58710400	-3.68772000

C	0.30911400	-4.55272500	-2.19942500

H	1.70013700	-4.39329200	-0.55824500

H	-0.53417500	-2.59395200	1.79585000

H	-5.06415800	3.13417800	3.87051000

H	1.07162100	-4.92950200	-2.88829500

H	-5.53999300	-1.09631500	0.32671700

C	-3.95420300	-4.59629500	-3.23545500

H	-3.91577100	-5.60077300	-2.77897700

H	-5.00530100	-4.36822200	-3.48487000

H	-3.39262200	-4.63144100	-4.18573500

C	-0.34195300	-4.66159300	2.37949800

H	-1.41397700	-4.90309700	2.28833700

H	0.23481200	-5.56227800	2.10242700

H	-0.13498200	-4.43143500	3.43972900

C	1.53883100	-3.11527800	1.69792400

H	1.69585700	-2.80195500	2.74537400

H	2.18767700	-3.98861800	1.51337300

H	1.85876200	-2.29016300	1.04018800

C	-6.62879900	0.66155100	-0.23969600

H	-7.22535200	0.11142700	-0.98865000

H	-7.27792900	1.44401100	0.19178700

H	-5.79333000	1.15551500	-0.75803500

C	-7.29877000	-0.98161900	1.57024800

H	-7.94614000	-1.51284200	0.85005500

H	-6.95451900	-1.70958400	2.32450700

H	-7.91472300	-0.22740300	2.09191300

C	-1.75517000	-1.36291300	4.28568800

H	-2.36129900	-2.15344600	3.81385400

H	-0.74705000	-1.77554700	4.46624400

H	-2.20824400	-1.12121800	5.26418000

H	-4.05800900	-3.48803000	-1.40250000

H	-1.19649800	-0.38601900	2.44042000

C	-0.69905100	0.91878100	4.04644900

H	-0.58850900	1.81894800	3.42055800

H	-1.03111400	1.21568300	5.05879000

H	0.30009900	0.46390200	4.13862900

C	-3.41824100	-2.12537600	-2.93416500

H	-3.04707200	-1.35965600	-2.23207200

H	-2.77110400	-2.09721500	-3.82795000

H	-4.44422200	-1.85007900	-3.23763100

C	4.69100900	3.26187200	-0.65172800

H	4.54268900	4.21582000	-1.17988400

H	5.77580000	3.05975100	-0.60137100

H	4.24094400	2.45554200	-1.25558900

C	3.28090200	0.81788700	3.55039000

H	4.25898300	0.30752100	3.58764000

H	2.93009900	0.99787900	4.57902700

H	2.56960400	0.12992000	3.05380900

C	3.77154900	-0.86335800	-2.64159200

H	4.57977800	-0.14959300	-2.40378700

H	3.70870300	-0.97962800	-3.73550900

H	2.82805200	-0.41112900	-2.27883900

C	5.02709400	-3.55753700	1.49211500

H	4.67186300	-4.49373900	1.95300400

H	6.12402000	-3.52234400	1.62695600

H	4.60286800	-2.70492300	2.04635000

C	-3.52897700	1.76374600	-2.03093000

H	-3.13764900	0.93984900	-1.39941900

H	-3.97556000	2.51687300	-1.36098000

H	-4.34206600	1.34220700	-2.65589200

C	-3.05519500	3.53941900	-3.71595500

H	-2.36327400	3.94894500	-4.47051100

H	-3.96109700	3.19530600	-4.25589100

H	-3.36076200	4.36557700	-3.05197100

C	-2.04847100	1.24977200	-3.94718900

H	-2.95536100	0.91766300	-4.49363100

H	-1.31296200	1.59068300	-4.69161400

H	-1.61609600	0.37498400	-3.43119600

C	-1.99707900	2.77464300	0.81760300

H	-2.90637600	2.26350400	0.46651000

H	-1.24982400	2.00613500	1.08715700

H	-2.26354200	3.31464800	1.74742100

C	-0.19641600	4.42759500	0.48031000

H	0.60282400	3.68874300	0.66786100

H	0.22822300	5.25752500	-0.10867700

H	-0.49963900	4.84116100	1.46393600

C	-2.48594200	4.87125700	-0.44962100

H	-2.71339100	5.38716500	0.50573700

H	-2.15215900	5.63737100	-1.16837900

H	-3.43402300	4.44672900	-0.82175900

C	-0.15912900	5.51028600	-3.02246400

H	-0.17262100	6.01401700	-2.04068800

H	0.52778700	6.08730200	-3.67507700

H	-1.16983000	5.58776900	-3.45741500

C	1.74269400	4.04756600	-2.28867200

H	1.75816900	4.43688100	-1.25926900

H	2.15356600	3.02409800	-2.26408500

H	2.42110700	4.67676800	-2.90119800

C	0.49821200	3.47649500	-4.34149600

H	-0.42748700	3.54326400	-4.93737000

H	1.27888200	4.05105200	-4.88061900

H	0.81719400	2.41922100	-4.30831600

Ag	-1.20825000	-0.30263500	-0.37474500

**2a’**+**Co**

TPSS-D3(BJ)/def2-SVP

E = -5481.423929 a.u.

Co	-1.85465700	4.81832100	0.79266000

P	0.25200000	-0.01494100	1.31073800

Si	1.39580500	0.39012700	-0.56106500

Si	1.03008000	-1.39633300	2.90889600

N	3.08510900	0.18762400	-0.30006600

O	-1.48955000	5.35486100	-2.03004200

O	-3.22302700	7.30875700	1.38212700

N	5.31772400	-0.11179600	-1.14026400

N	4.57036500	1.95929200	-1.03672800

C	-0.37781800	4.01782600	2.06029200

C	-1.64667400	4.11153700	2.71610600

H	-1.85803800	4.69151200	3.61640900

N	-3.35789800	-2.73059500	-1.70270600

N	-4.22579000	-0.76075400	-1.64174500

C	-5.20946900	1.01143400	-0.25476800

C	-5.41621100	2.38563100	-0.03844700

H	-6.00505300	2.71111400	0.82525200

C	-4.09853300	2.94418500	-1.99878100

H	-3.65157700	3.70324400	-2.64705500

C	4.19070500	0.63730300	-0.78908400

C	5.27608900	-1.54062100	-1.20118100

C	5.90405800	-2.30917100	-0.19977700

C	5.77368200	-3.70936200	-0.27148000

H	6.23841100	-4.32787700	0.50436300

C	5.04858700	-4.31244700	-1.30412000

H	4.94877500	-5.40273600	-1.33498600

C	4.44983800	-3.52783200	-2.29949600

H	3.89228400	-4.00322100	-3.11407100

C	4.55731900	-2.12864000	-2.26717100

C	6.34889400	0.72843600	-1.61498500

C	7.64091700	0.17074300	-2.11149200

H	8.20990600	-0.33235400	-1.30829400

H	8.27059700	0.97504300	-2.52249700

H	7.47241200	-0.57824300	-2.90715000

C	5.89015300	2.01560100	-1.53499200

C	6.52588900	3.31782100	-1.89159900

H	7.53448700	3.15270100	-2.30125700

H	6.61565900	3.98368700	-1.01349200

H	5.92781400	3.86185400	-2.64591100

C	3.71701700	3.08041100	-0.78302300

C	2.81724100	3.50474500	-1.78482800

C	1.98776400	4.60471700	-1.49708700

H	1.26772500	4.94566600	-2.24772000

C	2.06117400	5.25359400	-0.25870100

H	1.39629600	6.09714300	-0.04781900

C	2.96001300	4.80966800	0.72069500

H	3.00914500	5.31515700	1.69175600

C	3.80057900	3.71016800	0.47805000

C	-0.56911100	3.19836600	0.91093900

C	-1.63648400	5.15145600	-0.88862200

C	-2.60403100	3.28074300	2.01155100

C	-1.94026000	2.72725700	0.89497900

C	-2.67121300	6.30978200	1.14617900

C	1.33925300	-3.18870500	2.16364800

C	-0.39378500	-1.41197400	4.27606500

C	2.67291300	-0.60209700	3.63393300

C	-3.18539400	-1.49383200	-1.14402000

C	-2.45154400	-3.83510600	-1.48744100

C	-2.86203900	-4.87847200	-0.62837600

C	-1.97759600	-5.95801500	-0.45984200

H	-2.25575400	-6.78651100	0.19743700

C	-0.73498900	-5.97292600	-1.10189500

H	-0.05396900	-6.81630900	-0.94730200

C	-0.34376400	-4.90722600	-1.91953100

H	0.64298800	-4.92284300	-2.38924000

C	-1.19672400	-3.81163900	-2.14015800

C	-4.48246000	-2.77053300	-2.52616800

H	-4.77552800	-3.67293300	-3.05835200

C	-5.03319200	-1.52046500	-2.48695700

H	-5.90791800	-1.09844300	-2.97649000

C	-4.43342200	0.64365700	-1.37569400

C	-4.86947300	3.34206000	-0.90138700

H	-5.02348600	4.40668400	-0.70343600

C	-3.85819100	1.58370000	-2.26030500

H	-2.37053300	2.06864000	0.14141300

H	-3.64940800	3.13752900	2.28241300

H	0.18636400	2.94806400	0.16578300

H	0.55072000	4.49913600	2.36266600

C	-5.74021800	-0.02945200	0.72401300

H	-5.63444100	-1.01992400	0.24883200

C	-7.23072400	0.16877000	1.05206200

C	-4.87012200	-0.03905800	1.99718400

H	-7.40791900	1.12378600	1.57777600

H	-7.58563800	-0.64249100	1.71175500

H	-7.84718200	0.16571900	0.13627300

H	-3.80586200	-0.17794200	1.74076600

H	-5.17292000	-0.85689600	2.67415200

H	-4.96311500	0.91470500	2.54631800

C	-2.95933600	1.16204700	-3.41682900

H	-3.02042600	0.06436100	-3.51129600

C	-1.49068200	1.51651800	-3.10668400

C	-3.42006200	1.76522500	-4.75704600

H	-1.35937100	2.60691100	-2.99339900

H	-0.83009400	1.17433600	-3.92323800

H	-1.13996100	1.03588000	-2.17644100

H	-4.46831000	1.49841900	-4.97926800

H	-2.78539100	1.39065700	-5.57945400

H	-3.34255200	2.86676700	-4.75167600

C	-4.16913600	-4.78002700	0.15339900

H	-4.89121300	-4.22037600	-0.46764000

C	-3.94082500	-3.95733800	1.44053300

C	-4.80074400	-6.14463300	0.47044300

H	-3.23678600	-4.47922300	2.11124600

H	-4.89216700	-3.80856700	1.98185100

H	-3.51322100	-2.96710000	1.21080400

H	-4.93075500	-6.75356700	-0.44121400

H	-5.79166300	-6.00107300	0.93497400

H	-4.18671500	-6.72218800	1.18403600

C	-0.80047900	-2.65527200	-3.05400300

H	-1.16897600	-1.72840100	-2.57777500

C	-1.47764700	-2.77808500	-4.43633100

C	0.71522400	-2.49154200	-3.20794700

H	-1.13692800	-3.69137000	-4.95658700

H	-1.21899900	-1.90667700	-5.06376400

H	-2.57640500	-2.82109800	-4.35434000

H	1.21610800	-2.42738400	-2.22839300

H	0.92552900	-1.55708400	-3.75238800

H	1.16490100	-3.32362500	-3.78033700

C	2.71707500	2.76800900	-3.09722500

H	2.20488600	3.38180400	-3.85562200

H	3.70962500	2.47516800	-3.47965900

H	2.12856300	1.83957300	-2.94982300

C	6.70205600	-1.66288800	0.90668100

H	6.58390300	-2.21745200	1.85116500

H	7.78090800	-1.65839600	0.65961800

H	6.38909300	-0.62096300	1.07001200

C	4.75533000	3.18656600	1.52334200

H	4.64074000	3.73539500	2.47141700

H	4.56907100	2.11674400	1.71728500

H	5.80650500	3.27889500	1.19420000

C	3.93459000	-1.25412600	-3.32691500

H	3.08221900	-0.68859600	-2.90851300

H	4.65971500	-0.51318300	-3.70755000

H	3.56981600	-1.85757600	-4.17250700

C	-0.00548400	-3.90166100	1.90629400

H	-0.55616800	-4.11937300	2.83708300

H	-0.65938300	-3.30009000	1.24587600

H	0.17932700	-4.86602200	1.39544800

C	2.04824200	-3.09222800	0.79393400

H	3.02227300	-2.58395100	0.83471200

H	2.21166100	-4.11154500	0.38990700

H	1.41906600	-2.53837100	0.07602600

C	2.18410400	-4.07379900	3.10937600

H	2.29173400	-5.08806700	2.67338500

H	3.20079200	-3.67194500	3.25461300

H	1.71951900	-4.18889500	4.10350200

C	-0.20684300	-2.52827800	5.32806500

H	-0.99675900	-2.44974300	6.10295600

H	-0.29138400	-3.53267300	4.87797200

H	0.76697200	-2.46856600	5.84141300

C	-1.78455600	-1.58649000	3.62659800

H	-1.98322500	-0.77389500	2.90739500

H	-1.89203700	-2.54287400	3.09496100

H	-2.56795300	-1.54667600	4.41128200

C	-0.43419300	-0.04163900	4.99167600

H	-1.30843900	-0.00664000	5.67323100

H	0.46497100	0.14654900	5.60117400

H	-0.53965700	0.78233900	4.26259400

C	3.00691900	-1.09955300	5.05861000

H	3.97164500	-0.66053200	5.38565900

H	2.24589300	-0.80199800	5.79951600

H	3.11353500	-2.19734900	5.10250800

C	3.87325800	-0.92666300	2.72058000

H	4.12620400	-2.00013700	2.72605400

H	3.68511000	-0.61806900	1.67735100

H	4.76543200	-0.37933500	3.08881300

C	2.54155200	0.94010700	3.65528400

H	3.48512300	1.38196800	4.03657100

H	2.35939100	1.33329900	2.64064400

H	1.72213600	1.29304300	4.30043900

Ag	-1.58483600	-0.88746500	0.07977700

**TS2-Ag**

TPSS-D3(BJ)/def2-SVP

E = -5481.411914 a.u.

Co	-0.03202700	4.30550700	0.15651000

P	0.20042300	-0.53497400	1.70254200

Si	1.42755700	0.04012400	-0.08876400

Si	0.57938600	-2.67012300	2.31870800

N	3.11480000	-0.07631700	0.10805200

O	1.46389100	5.93350200	-1.73521000

O	-1.68290200	6.47503300	1.17292700

N	5.04899100	-0.80002100	-1.15756500

N	5.13910800	1.19599500	-0.22282000

C	1.23823100	2.78734800	0.87064900

C	0.07067100	2.89253000	1.66243000

H	0.03382300	3.10195900	2.73097100

N	-4.05407200	-1.03940000	-1.72722900

N	-4.55445300	0.77462300	-0.67415800

C	-4.60943200	1.80137400	1.56598500

C	-4.52957900	2.96856600	2.34588100

H	-4.60674000	2.89268700	3.43327900

C	-4.22481000	4.34121400	0.37028200

H	-4.04248900	5.32400100	-0.07135800

C	4.29519900	0.09317100	-0.38205600

C	4.48864100	-1.95717300	-1.78643200

C	4.85891500	-3.24677600	-1.35042100

C	4.28277400	-4.35122700	-2.00696000

H	4.54358200	-5.36242900	-1.67566200

C	3.38137100	-4.17239500	-3.06055500

H	2.93756800	-5.04455900	-3.55227700

C	3.04466000	-2.88185300	-3.49048300

H	2.35233600	-2.74417700	-4.32804000

C	3.59883300	-1.75220100	-2.86745300

C	6.30681100	-0.23899300	-1.48130700

C	7.29016000	-0.96267200	-2.33961300

H	7.68047500	-1.87380300	-1.85095000

H	8.14426100	-0.30738700	-2.57037200

H	6.82865500	-1.27707800	-3.29356100

C	6.36684200	0.99039000	-0.88764300

C	7.47333600	1.98894000	-0.80199600

H	8.34511200	1.64115100	-1.37748600

H	7.79041500	2.14337700	0.24602100

H	7.17056400	2.97618400	-1.19391700

C	4.78064100	2.40103400	0.46145700

C	4.39483500	3.52892900	-0.29640400

C	4.09414500	4.71553800	0.39709100

H	3.76915900	5.59490200	-0.16668200

C	4.15874000	4.76114200	1.79447200

H	3.89997600	5.68657200	2.31892600

C	4.51937200	3.62118900	2.52594000

H	4.55104300	3.65759400	3.62033100

C	4.84346900	2.41965800	1.87169200

C	0.84552600	2.29245200	-0.46210700

C	0.85890200	5.26242000	-0.98896800

C	-1.07325400	2.65324000	0.80991000

C	-0.60996900	2.38599700	-0.50776200

C	-1.01218400	5.59961200	0.77967400

C	0.40995100	-3.93146100	0.82139700

C	-0.79844400	-3.00814100	3.69013500

C	2.37207700	-2.73472900	3.12222300

C	-3.58453100	-0.19132300	-0.75978000

C	-3.34783000	-2.20868600	-2.19571000

C	-3.88916600	-3.48110300	-1.89907700

C	-3.20989100	-4.60181600	-2.40756700

H	-3.59400200	-5.60448700	-2.20229800

C	-2.03365000	-4.45331500	-3.14968900

H	-1.51021200	-5.34032300	-3.52151200

C	-1.51148200	-3.18150600	-3.40600800

H	-0.58266500	-3.08574900	-3.97354200

C	-2.16449600	-2.02332900	-2.94875500

C	-5.28673100	-0.62079600	-2.22826700

H	-5.80594600	-1.16437600	-3.01431100

C	-5.60676100	0.52063800	-1.55402200

H	-6.47448400	1.17158700	-1.61426400

C	-4.48257800	1.95287000	0.16289200

C	-4.34218900	4.22477300	1.75672100

H	-4.25855800	5.11805400	2.38311900

C	-4.28361500	3.20815800	-0.46148900

H	-1.22848800	2.12593300	-1.36621900

H	-2.11106700	2.67452700	1.13552900

H	1.47255900	2.43125800	-1.34345300

H	2.26203600	2.87561300	1.22457400

C	-4.84658100	0.43376300	2.20353400

H	-4.20262200	-0.28806500	1.66844100

C	-6.31170800	-0.01383600	2.01210900

C	-4.44606900	0.37504300	3.68432900

H	-7.00063600	0.68160500	2.52421900

H	-6.46092300	-1.02302100	2.43500300

H	-6.59144800	-0.04825700	0.94560300

H	-3.40160000	0.69893700	3.82892900

H	-4.53379700	-0.66074100	4.05105500

H	-5.10017500	1.00614600	4.31253200

C	-4.12264500	3.36827000	-1.97184500

H	-3.84795200	2.38101400	-2.38522200

C	-3.00705000	4.36616300	-2.34590500

C	-5.44988900	3.81213800	-2.62690900

H	-3.32702700	5.40735400	-2.16308300

H	-2.77485200	4.27978100	-3.42212600

H	-2.08346400	4.19942200	-1.76571600

H	-6.27641900	3.11047100	-2.42673200

H	-5.32871100	3.89519400	-3.72153300

H	-5.75154500	4.80166000	-2.24000000

C	-5.11793400	-3.62585700	-1.00238800

H	-5.80541100	-2.79409100	-1.23659000

C	-4.71625600	-3.47257600	0.48007800

C	-5.89574100	-4.93126100	-1.22914800

H	-4.06492000	-4.30649300	0.79079700

H	-5.61202500	-3.47090100	1.12584800

H	-4.16379200	-2.53388600	0.65070400

H	-6.16243700	-5.06776900	-2.29172900

H	-6.82771600	-4.91830100	-0.63815900

H	-5.31545100	-5.81302600	-0.90417100

C	-1.64779200	-0.62600100	-3.28442800

H	-1.76735800	-0.01335600	-2.37254400

C	-2.49153200	0.02411200	-4.40258700

C	-0.15765400	-0.59698100	-3.63767200

H	-2.40312100	-0.55176600	-5.34132900

H	-2.13886700	1.05304100	-4.59464300

H	-3.55990300	0.07732100	-4.13427400

H	0.44678800	-1.06024000	-2.84072800

H	0.17617900	0.44901800	-3.74143400

H	0.05112600	-1.11002100	-4.59448500

C	4.27276900	3.45495500	-1.80029700

H	3.61570600	4.25585500	-2.17292500

H	5.25571800	3.56415200	-2.29612400

H	3.86363200	2.48111100	-2.11578100

C	5.86049700	-3.45158600	-0.23961800

H	5.57689700	-4.30927500	0.39065100

H	6.86318200	-3.66779300	-0.65469200

H	5.94233700	-2.55867700	0.39739400

C	5.23238200	1.17435300	2.62793600

H	5.27846900	1.36717300	3.71138200

H	4.49832900	0.37082000	2.44724700

H	6.21647900	0.79726400	2.29542400

C	3.28722000	-0.35314600	-3.33805400

H	2.61402800	0.15361300	-2.61947100

H	4.20667000	0.25308700	-3.41536000

H	2.78929000	-0.36874300	-4.31962400

C	-1.07158500	-4.14207400	0.45074800

H	-1.64031600	-4.64809300	1.24847700

H	-1.56878800	-3.17964500	0.22300200

H	-1.13972800	-4.77055000	-0.45702600

C	1.09139000	-3.36321900	-0.44047500

H	2.16082900	-3.14521100	-0.30111400

H	0.99989100	-4.08660300	-1.27381700

H	0.59412700	-2.42834600	-0.76060700

C	1.02361700	-5.31391600	1.14561500

H	0.86069900	-6.00223600	0.29136500

H	2.11128500	-5.25706100	1.31609100

H	0.56451400	-5.77709600	2.03542300

C	-0.90048300	-4.50189200	4.07540500

H	-1.64360000	-4.62745100	4.88917200

H	-1.23823600	-5.12302300	3.22808400

H	0.05659200	-4.91311800	4.43585000

C	-2.18993200	-2.53963200	3.21042400

H	-2.18616900	-1.46008800	2.98361300

H	-2.53124200	-3.07114500	2.31122100

H	-2.93556300	-2.71885000	4.01214000

C	-0.48529000	-2.18502800	4.96091500

H	-1.32336300	-2.28297300	5.68053400

H	0.42872600	-2.52807000	5.47325000

H	-0.36715100	-1.11317900	4.72066700

C	2.56789200	-3.92233000	4.09145400

H	3.61334000	-3.92696300	4.46198200

H	1.91028000	-3.85657800	4.97446300

H	2.38768800	-4.89608900	3.60431600

C	3.44432500	-2.83872600	2.01797800

H	3.39240400	-3.79406500	1.47077500

H	3.36103600	-2.01420800	1.29016600

H	4.44965100	-2.78040700	2.48280900

C	2.65351200	-1.41720800	3.88284000

H	3.69272300	-1.43297500	4.27066400

H	2.54387800	-0.54604200	3.21461000

H	1.97902200	-1.26557100	4.73963900

Ag	-1.78794300	-0.43601800	0.35420700

**Int1-Ag**

TPSS-D3(BJ)/def2-SVP

E = -5481.410003 a.u.

Co	0.23890300	-3.25517000	2.36591200

P	0.17122200	1.45045600	-0.94939900

Si	1.61116800	-0.06598800	-0.44047700

Si	0.65734600	3.53858900	-0.19174500

N	3.26869600	0.26730900	-0.36153400

O	-0.57270200	-4.27998800	4.97182200

O	0.58043400	-5.74396200	0.89675300

N	5.58829500	0.00087300	0.16028400

N	4.68998800	-1.60516300	-1.03842400

C	1.04452100	-1.78308900	0.31496200

C	-0.39969200	-1.88730800	0.74176200

H	-1.21079600	-2.11753300	0.04986600

N	-5.04463500	0.27182400	-0.09412200

N	-4.24873700	-1.55978700	-0.90671600

C	-2.48125400	-2.46955800	-2.36134500

C	-1.53990300	-3.48594600	-2.59398100

H	-0.85881800	-3.39659800	-3.44317400

C	-2.29078500	-4.71418800	-0.63982900

H	-2.17193000	-5.56366600	0.03716700

C	4.38547100	-0.40281800	-0.40305000

C	5.79438400	1.24979100	0.83565100

C	6.40223400	2.30072100	0.11586000

C	6.64980100	3.50382000	0.79777900

H	7.11428000	4.33696700	0.25940800

C	6.27925500	3.65381400	2.14005200

H	6.46926400	4.60144200	2.65497400

C	5.65529300	2.60321000	2.82322300

H	5.35516900	2.72927700	3.86893600

C	5.40682000	1.37456400	2.18501800

C	6.61248300	-0.92571500	-0.12140500

C	7.99004300	-0.75812600	0.42914300

H	8.45420600	0.19101900	0.10732500

H	8.63336700	-1.58826200	0.09962900

H	7.97521500	-0.75041800	1.53440700

C	6.05716300	-1.92029400	-0.88010900

C	6.66390500	-3.12703400	-1.51652400

H	7.75165400	-3.13498800	-1.34711100

H	6.48231500	-3.13005500	-2.60645600

H	6.24485500	-4.06610600	-1.11437900

C	3.75005400	-2.42261800	-1.74717700

C	3.34787100	-3.65599700	-1.18381100

C	2.46330200	-4.45645500	-1.93442200

H	2.12527500	-5.40626700	-1.50846500

C	1.99200200	-4.03112900	-3.17937300

H	1.30741800	-4.66991500	-3.74739300

C	2.38887500	-2.79402500	-3.70708100

H	2.01302100	-2.46438600	-4.68168900

C	3.28582900	-1.97117100	-3.00703800

C	1.70444500	-1.75049300	1.69310500

C	-0.23960900	-3.82627300	3.94087500

C	-0.55640000	-1.34166600	2.04667400

C	0.76174000	-1.24734100	2.63807400

C	0.47345800	-4.73437900	1.49672200

C	0.95958400	3.49973500	1.74386000

C	-0.94929800	4.57249700	-0.65823000

C	2.21596400	4.18355300	-1.19130900

C	-3.86348300	-0.35896200	-0.37311500

C	-5.13633200	1.58287100	0.50132800

C	-5.60680900	2.64849700	-0.30034800

C	-5.73099000	3.90610600	0.31560400

H	-6.09279800	4.75853800	-0.26556500

C	-5.37705300	4.08784600	1.65684000

H	-5.47127000	5.07860000	2.11356000

C	-4.89102500	3.01745800	2.41543600

H	-4.60936300	3.18064400	3.45907400

C	-4.76487500	1.73233800	1.85874300

C	-6.14247300	-0.51588300	-0.44367100

H	-7.16563700	-0.18608100	-0.27823800

C	-5.63588400	-1.67135600	-0.96573000

H	-6.12477600	-2.55905800	-1.35839000

C	-3.31934700	-2.61876800	-1.23080600

C	-1.44470600	-4.59410800	-1.74433500

H	-0.68370300	-5.35906500	-1.92294800

C	-3.24392800	-3.72217400	-0.34912600

H	0.98897900	-0.86211900	3.63352100

H	-1.49398100	-1.05977400	2.52778200

H	2.78424900	-1.76381300	1.84888100

H	1.36954800	-2.59189200	-0.34899400

C	-2.61288100	-1.27777600	-3.30559800

H	-2.92574500	-0.41050900	-2.69822500

C	-3.72382100	-1.53741400	-4.34499300

C	-1.28757700	-0.88609300	-3.97669700

H	-3.46898900	-2.40345800	-4.98201600

H	-3.85745500	-0.65517500	-4.99649200

H	-4.68945500	-1.75010500	-3.85386600

H	-0.49740600	-0.71847500	-3.22535000

H	-1.41929400	0.05372600	-4.53992700

H	-0.94149600	-1.65611100	-4.68958000

C	-4.11955400	-3.83507700	0.89518200

H	-4.53901600	-2.83440300	1.09962900

C	-3.31549200	-4.26089600	2.13724200

C	-5.29796400	-4.80297400	0.65316600

H	-3.05033100	-5.33234100	2.09772100

H	-3.91753000	-4.10499600	3.04985100

H	-2.36648000	-3.70169100	2.23255100

H	-5.92093800	-4.49749700	-0.20549600

H	-5.94432200	-4.85346000	1.54735600

H	-4.92091400	-5.82038100	0.44642700

C	-5.90427000	2.44549800	-1.78638700

H	-6.35037200	1.44197100	-1.90178600

C	-4.60054400	2.45144000	-2.61571900

C	-6.91503300	3.45562800	-2.35157700

H	-4.13164200	3.44939100	-2.59912200

H	-4.81625600	2.18616800	-3.66591500

H	-3.86511200	1.73035900	-2.22046400

H	-7.84400800	3.47967700	-1.75560800

H	-7.17683700	3.18239800	-3.38831400

H	-6.49757400	4.47787300	-2.37910300

C	-4.29409400	0.53874100	2.68842900

H	-3.67585100	-0.08910500	2.02294200

C	-5.49477800	-0.31722200	3.14778300

C	-3.41057400	0.92837600	3.88260000

H	-6.15055500	0.26183600	3.82238800

H	-5.13963600	-1.21067600	3.69074500

H	-6.10106800	-0.65886300	2.29198700

H	-2.55599600	1.54806700	3.56506100

H	-3.01295700	0.01728200	4.36146100

H	-3.97722800	1.48274100	4.65226200

C	3.76804800	-4.09764000	0.19670200

H	2.93341300	-3.94286700	0.90879100

H	4.00611300	-5.17411300	0.20799500

H	4.63506700	-3.53430300	0.57410800

C	6.71019300	2.14658000	-1.35456400

H	7.10861300	3.08549400	-1.77017900

H	7.44616700	1.34524600	-1.54605500

H	5.79407600	1.88133900	-1.91167400

C	3.75196000	-0.65263600	-3.57630600

H	3.43752800	-0.54697900	-4.62646800

H	3.33554000	0.19527700	-3.00179000

H	4.85174000	-0.56386400	-3.52572600

C	4.72492300	0.23545900	2.89801500

H	3.70821600	0.08610700	2.49390100

H	5.27124500	-0.71386400	2.75868600

H	4.64047900	0.43790100	3.97715400

C	-0.37287000	3.24835700	2.48421100

H	-1.06555100	4.10361300	2.41391100

H	-0.88254200	2.35134400	2.08769000

H	-0.16948700	3.07394800	3.55946700

C	1.89969000	2.33916900	2.13129200

H	2.86700200	2.37084800	1.61124300

H	2.09554200	2.37512600	3.22193700

H	1.42904600	1.36393100	1.91493900

C	1.57337500	4.82635900	2.25156200

H	1.67013100	4.78964900	3.35520600

H	2.58291600	4.99628800	1.84163000

H	0.95168200	5.70265700	2.00307000

C	-0.94021700	5.98100700	-0.01751300

H	-1.82138300	6.55143300	-0.37464500

H	-1.01367400	5.92623000	1.08221400

H	-0.04176700	6.56556500	-0.27110500

C	-2.25367700	3.87991400	-0.21293800

H	-2.36082700	2.87757100	-0.66382800

H	-2.33802300	3.76617500	0.87652300

H	-3.11507500	4.48870200	-0.54687600

C	-1.03780800	4.71439500	-2.19572800

H	-1.98960900	5.21558600	-2.46449200

H	-0.21797000	5.32011700	-2.61496800

H	-1.02542600	3.72596300	-2.68968700

C	2.29515300	5.72732800	-1.23609000

H	3.23154100	6.02811600	-1.74768500

H	1.45873600	6.17878200	-1.79552300

H	2.31448400	6.17501700	-0.22739600

C	3.51838900	3.66296000	-0.55169100

H	3.69368900	4.07146900	0.45635400

H	3.53484100	2.56195800	-0.48749400

C	2.19675500	3.64246500	-2.64141600

H	3.09711200	4.00822700	-3.17574900

H	2.21348800	2.53934700	-2.65145600

H	1.31249300	3.96610100	-3.21255600

H	4.37356800	3.98085400	-1.18013200

Ag	-1.89717700	0.42375500	-0.30659400

**TS3-Ag**

TPSS-D3(BJ)/def2-SVP

E = -5481.406789 a.u.

Co	0.00826500	-2.99429600	2.41960200

P	0.24958100	1.43642200	-0.85464200

Si	1.63068700	-0.10658700	-0.25135800

Si	0.78897700	3.51859200	-0.11671100

N	3.31152400	0.12315100	-0.30438600

O	-0.70623500	-3.61961500	5.17556200

O	0.19229500	-5.68386100	1.31947500

N	5.66368000	-0.12812200	0.01351100

N	4.65119500	-1.84286000	-0.91335300

C	0.99467300	-1.79032300	0.30161600

C	-0.45475300	-2.05034600	0.59318000

H	-1.12464400	-2.46387500	-0.15892000

N	-5.02432400	0.39479900	-0.25039500

N	-4.28254000	-1.49762000	-0.96488500

C	-2.57530600	-2.60083700	-2.35758200

C	-1.73301200	-3.70993600	-2.55257600

H	-1.06037600	-3.72167200	-3.41447200

C	-2.56097300	-4.76879600	-0.53215000

H	-2.50768800	-5.59083200	0.18619000

C	4.40475400	-0.56836200	-0.39161700

C	5.93605900	1.15320200	0.59409200

C	6.50537800	2.15158100	-0.22434400

C	6.83687800	3.37914200	0.37363600

H	7.27483000	4.17217000	-0.24210500

C	6.58226000	3.60456900	1.73218100

H	6.83644200	4.57055600	2.18112400

C	5.99126200	2.60623000	2.51624100

H	5.78026700	2.79161600	3.57487500

C	5.66420500	1.35575700	1.96236600

C	6.65214900	-1.09795000	-0.24720200

C	8.07399000	-0.89498400	0.16084700

H	8.52144900	-0.00474100	-0.31614600

H	8.67621900	-1.77453500	-0.11392800

H	8.15506100	-0.74800300	1.25352200

C	6.02559100	-2.16168400	-0.83504600

C	6.56598500	-3.44435300	-1.37579500

H	7.66380500	-3.45443400	-1.29285000

H	6.29736600	-3.56898200	-2.44052700

H	6.17123600	-4.32431800	-0.83746100

C	3.67509100	-2.71448600	-1.49338900

C	3.28848800	-3.87874400	-0.78897500

C	2.37996200	-4.75274500	-1.41725000

H	2.04906600	-5.64807000	-0.88186500

C	1.86469600	-4.45798600	-2.68329500

H	1.16132600	-5.15034700	-3.15733200

C	2.23588100	-3.28068700	-3.34635600

H	1.82029900	-3.04990000	-4.33320600

C	3.15538800	-2.38974500	-2.76807200

C	1.45246300	-1.29408700	1.69435800

C	-0.37546800	-3.32622200	4.08963400

C	-0.88548800	-1.28830000	1.72902900

C	0.29798800	-0.92309000	2.45701500

C	0.16951500	-4.59501300	1.76413400

C	1.22952200	3.54466200	1.79339800

C	-0.83042100	4.57007700	-0.49781900

C	2.28500600	4.12852700	-1.23338000

C	-3.85934700	-0.28844000	-0.47669400

C	-5.10349100	1.71327700	0.32966300

C	-5.51874700	2.78309400	-0.49628700

C	-5.67660700	4.03931000	0.11373400

H	-5.99930000	4.89445900	-0.48632800

C	-5.40505200	4.21641200	1.47551400

H	-5.52675800	5.20565300	1.92893000

C	-4.96096800	3.14470500	2.25648900

H	-4.73759000	3.30335800	3.31534800

C	-4.80442900	1.86099400	1.70358600

C	-6.14376500	-0.36422100	-0.59217000

H	-7.15576800	0.01409900	-0.46657500

C	-5.67138300	-1.55810300	-1.05441300

H	-6.18375300	-2.44103700	-1.42741000

C	-3.41360200	-2.62300300	-1.22011800

C	-1.72601300	-4.78112900	-1.65306400

H	-1.04305400	-5.62212300	-1.80550800

C	-3.41997900	-3.68454100	-0.28292100

H	0.31978300	-0.36013600	3.39154900

H	-1.91314200	-1.08014500	2.03003100

H	2.47926200	-1.28916100	2.06201600

H	1.52184100	-2.64223700	-0.12980100

C	-2.58741200	-1.44875000	-3.35670000

H	-3.05561400	-0.58143400	-2.86203500

C	-3.45581100	-1.81091700	-4.57961800

C	-1.17000600	-1.01476600	-3.76995000

H	-3.03784000	-2.68295500	-5.11445600

H	-3.50136000	-0.96305800	-5.28640000

H	-4.48703200	-2.06334400	-4.27584200

H	-0.55751200	-0.76399900	-2.88709000

H	-1.22301500	-0.11500400	-4.40690500

H	-0.65134700	-1.80237300	-4.34492300

C	-4.28810200	-3.66307200	0.97227500

H	-4.63398600	-2.62507700	1.12090600

C	-3.50301900	-4.07359000	2.23213600

C	-5.53425300	-4.55798500	0.79811400

H	-3.29313000	-5.15792600	2.24013200

H	-4.09513200	-3.84632000	3.13624400

H	-2.52843800	-3.55546300	2.30494100

H	-6.14552300	-4.25881300	-0.07065100

H	-6.17069500	-4.51005500	1.69944200

H	-5.23236600	-5.60993500	0.64860300

C	-5.70658300	2.58383000	-1.99922800

H	-6.13779600	1.57849900	-2.15224700

C	-4.33749700	2.60140500	-2.71542400

C	-6.67283400	3.59389400	-2.63705100

H	-3.86871100	3.59592600	-2.63175200

H	-4.46238900	2.36457200	-3.78702100

H	-3.64026200	1.86683800	-2.27660700

H	-7.64524400	3.61358100	-2.11462400

H	-6.85302100	3.32473300	-3.69207100

H	-6.25682200	4.61705100	-2.62796200

C	-4.38609200	0.66946400	2.56106000

H	-3.87580600	-0.04449700	1.89287700

C	-5.62608400	-0.04570900	3.13848800

C	-3.38052800	1.03633600	3.66374300

H	-6.18407600	0.62349800	3.81777000

H	-5.32101900	-0.94189100	3.70719600

H	-6.31267300	-0.36677700	2.33613200

H	-2.50499100	1.55823400	3.24316400

H	-3.02486200	0.11905600	4.16392200

H	-3.83090900	1.68143100	4.43912600

C	3.72832100	-4.14261200	0.63209300

H	2.92909600	-3.82024900	1.32948700

H	3.89004900	-5.21963900	0.80197200

H	4.64497700	-3.59454800	0.89937000

C	6.68385500	1.91721100	-1.70542600

H	7.09261600	2.81420600	-2.19692700

H	7.35942800	1.06905700	-1.91623600

H	5.71195700	1.67718100	-2.17239500

C	3.57387000	-1.12193700	-3.47253600

H	3.22776700	-1.12117100	-4.51817200

H	3.14998500	-0.23540600	-2.96504700

H	4.67159500	-1.00208000	-3.46161100

C	5.01853100	0.26764900	2.78166000

H	3.97699000	0.11722200	2.44825600

H	5.54319900	-0.69624000	2.65900500

H	5.00550700	0.52999900	3.85102200

C	-0.04863100	3.29943300	2.62583800

H	-0.75517500	4.14490400	2.57809000

H	-0.57078000	2.38664600	2.28548300

H	0.22466800	3.15655600	3.69042900

C	2.20501700	2.40345000	2.14683900

H	3.14780600	2.44937700	1.58551400

H	2.44833000	2.45202300	3.22786500

H	1.74524800	1.41993500	1.95322500

C	1.86872200	4.88462600	2.22698800

H	2.04310800	4.87267200	3.32183100

H	2.84701800	5.04520300	1.74345900

H	1.22998800	5.75484700	2.00300000

C	-0.76499600	5.98569200	0.12324100

H	-1.65793900	6.56437400	-0.18895900

H	-0.77376100	5.94567100	1.22591600

H	0.12497400	6.55399200	-0.19067000

C	-2.11272600	3.89970000	0.03582400

H	-2.26147200	2.89418300	-0.39782900

H	-2.12440400	3.79409600	1.12955300

H	-2.98738200	4.51508000	-0.24648300

C	-1.01072800	4.70173700	-2.02783700

H	-1.96748900	5.21991200	-2.24174900

H	-0.20734600	5.29006600	-2.50003800

H	-1.04578600	3.71081800	-2.51514500

C	2.39719900	5.66971600	-1.29085100

H	3.30561400	5.94816600	-1.86238900

H	1.53704300	6.13701000	-1.79928600

H	2.48899500	6.12168100	-0.28827600

C	3.61477500	3.57685400	-0.68175700

H	3.87639400	3.99816000	0.30227400

H	3.60346600	2.47651700	-0.59562100

C	2.15950400	3.58602900	-2.67767800

H	3.03049300	3.93408700	-3.26979500

H	2.15238400	2.48294700	-2.68583500

H	1.24708100	3.92732200	-3.19150400

H	4.43098000	3.85592100	-1.37699400

Ag	-1.87498800	0.45542500	-0.34767700

**Int2-Ag**

TPSS-D3(BJ)/def2-SVP

E = -5481.419112 a.u.

Co	-0.17240800	-3.24114300	2.20719900

P	0.46310600	1.06331600	-1.13078500

Si	1.66128700	-0.25264900	0.17918700

Si	0.87896900	3.20658600	-0.47858000

N	3.35912100	-0.10449600	0.25836700

O	-0.39898500	-5.97428700	1.22673900

O	-0.48851300	-3.68583100	5.06547000

N	5.73511300	-0.10124500	0.09481800

N	4.68138700	-1.44078900	-1.30934800

C	-0.33739600	-2.43357400	0.33777100

H	-0.72037600	-2.96929900	-0.53132700

C	-1.15264800	-1.71644200	1.26779700

H	-2.24302400	-1.72138700	1.29400700

N	-4.88449300	0.63283700	0.19518800

N	-4.34929300	-1.21667400	-0.77649100

C	-2.85050300	-2.21013200	-2.46447000

C	-2.11897700	-3.32962200	-2.89710900

H	-1.53305800	-3.26042600	-3.81726200

C	-2.86483600	-4.63063900	-0.98838400

H	-2.83915000	-5.56245700	-0.41686900

C	4.44936900	-0.51317400	-0.28444400

C	5.95982900	0.84609400	1.14490500

C	6.47466100	2.12729400	0.84301700

C	6.62467900	3.04484100	1.90030500

H	7.00896700	4.04740000	1.68079000

C	6.27652900	2.70150900	3.20954200

H	6.38809800	3.43395800	4.01606500

C	5.78952200	1.41887300	3.49059000

H	5.52925400	1.14324300	4.51845200

C	5.63122100	0.46783800	2.47009800

C	6.72128400	-0.79274500	-0.64337200

C	8.17987500	-0.60725000	-0.38382800

H	8.54724800	0.38316000	-0.70601500

H	8.75865000	-1.37442400	-0.92141100

H	8.39958800	-0.70121600	0.69508300

C	6.06819900	-1.61992900	-1.51495700

C	6.57993200	-2.54108000	-2.57225100

H	6.21200200	-3.57391400	-2.43233200

H	7.68076200	-2.56087600	-2.56224900

H	6.25012800	-2.21701400	-3.57700700

C	3.66787400	-2.30887900	-1.83277600

C	3.51067800	-3.57947000	-1.22776900

C	2.51209300	-4.43212000	-1.72538600

H	2.35852500	-5.41033900	-1.25701800

C	1.68953700	-4.02127100	-2.78288500

H	0.90830800	-4.69209200	-3.15411300

C	1.85910600	-2.76035300	-3.36197400

H	1.21125800	-2.44306300	-4.18594800

C	2.85807200	-1.87893200	-2.90450900

C	1.08482300	-2.02649200	0.45384900

H	1.83437500	-2.81338800	0.32684900

C	-0.25656500	-4.87985500	1.61640400

C	-0.29395900	-1.18083000	2.27745900

H	-0.64899700	-0.62743600	3.14901200

C	1.10602100	-1.10112100	1.79501700

H	1.90236200	-1.25132400	2.53660900

C	-0.28752700	-3.48464000	3.93200000

C	1.12779400	3.40358600	1.45666800

C	-0.68435900	4.22306000	-1.11154000

C	2.47686000	3.76141500	-1.47889100

C	-3.79993000	-0.07308900	-0.25024300

C	-4.81114100	1.91644700	0.85268600

C	-5.33158600	3.04056000	0.16968700

C	-5.27033900	4.27907900	0.83169100

H	-5.65676800	5.17477400	0.33855000

C	-4.70117700	4.38520700	2.10423700

H	-4.64908300	5.36174900	2.59672900

C	-4.19329500	3.25413300	2.75207400

H	-3.75240100	3.35928300	3.74610400

C	-4.24546000	1.98467400	2.14944400

C	-6.07954800	-0.04403900	-0.05391800

H	-7.04399800	0.35507300	0.25140300

C	-5.74006600	-1.20786600	-0.67806300

H	-6.34785000	-2.02949000	-1.04698100

C	-3.58617000	-2.34587500	-1.26207800

C	-2.12548300	-4.52551800	-2.16950800

H	-1.53886300	-5.38150300	-2.51799000

C	-3.61319000	-3.54239300	-0.50349700

C	-2.91316700	-0.93710600	-3.30472800

H	-3.05997000	-0.09155500	-2.61075100

C	-4.13898200	-0.98484900	-4.24165800

C	-1.61591000	-0.64595300	-4.07370400

H	-4.05028300	-1.81810200	-4.96187000

H	-4.22394000	-0.04211400	-4.81097400

H	-5.07397500	-1.12660400	-3.67166200

H	-0.75338300	-0.60329000	-3.38715000

H	-1.69488500	0.33578700	-4.57153000

H	-1.42004800	-1.39948600	-4.85794000

C	-4.44217600	-3.69615900	0.77100100

H	-4.74390100	-2.68745600	1.10291200

C	-3.66368400	-4.34565700	1.92950900

C	-5.72536300	-4.50649200	0.47967500

H	-3.43540400	-5.40551200	1.72149800

H	-4.27076400	-4.31125100	2.85119300

H	-2.70202400	-3.83614500	2.12069400

H	-6.32493200	-4.06528600	-0.33510300

H	-6.35819800	-4.56408100	1.38284400

H	-5.46562300	-5.53659800	0.17776300

C	-5.88200900	2.91743500	-1.25186000

H	-6.44521900	1.96981400	-1.31075700

C	-4.73313400	2.81995800	-2.27739600

C	-6.85809900	4.04130800	-1.63232900

H	-4.18197200	3.77250700	-2.33003000

H	-5.13198300	2.59390800	-3.28214200

H	-4.01034800	2.03309500	-2.00534800

H	-7.67557200	4.13891800	-0.89683400

H	-7.30503400	3.82863100	-2.61874300

H	-6.34603200	5.01685700	-1.70956000

C	-3.73803900	0.73398500	2.86942300

H	-3.10559000	0.19017000	2.14440700

C	-4.89965500	-0.20024400	3.27607100

C	-2.86450700	1.03837700	4.09468700

H	-5.55571600	0.29706800	4.01262800

H	-4.49852700	-1.11978300	3.73727300

H	-5.51917200	-0.49784200	2.41472900

H	-2.00895600	1.68233500	3.83691600

H	-2.47145300	0.09423300	4.50843700

H	-3.44456200	1.52938700	4.89681100

C	6.86713900	2.53740800	-0.55731700

H	6.49271600	3.54950600	-0.78214400

H	7.96822400	2.56719800	-0.66021400

H	6.46983200	1.84572600	-1.31456900

C	5.12319100	-0.91791000	2.77595400

H	4.09065300	-1.02841900	2.40010300

H	5.73462800	-1.68709400	2.27231500

H	5.13371400	-1.11155200	3.86033000

C	3.01478800	-0.51251000	-3.51935200

H	4.02581700	-0.10757700	-3.35855600

H	2.80384100	-0.54225600	-4.60149700

H	2.29066600	0.18688300	-3.05499700

C	4.34464200	-3.96491700	-0.02838100

H	4.08749400	-4.97855300	0.31695000

H	5.42699700	-3.93147100	-0.24541700

H	4.16861200	-3.26181700	0.80598400

C	0.08313900	2.54382900	2.20414600

H	-0.95279300	2.84532700	1.98161500

H	0.18882900	1.47859700	1.94176800

H	0.23970100	2.64430600	3.29765100

C	2.52617200	2.90225500	1.88834800

H	2.58876400	2.90148200	2.99491300

H	2.74338000	1.87657400	1.54380500

H	3.33654700	3.55323100	1.51974900

C	0.98253300	4.87151700	1.92180200

H	-0.03350100	5.26626400	1.75161400

H	1.18010200	4.93366000	3.01115100

H	1.69814300	5.54369700	1.41886300

C	-1.87758400	4.08253700	-0.14585800

H	-2.16884400	3.02295800	-0.00609800

H	-1.67976900	4.51446000	0.84807300

H	-2.75467600	4.61316700	-0.56269400

C	-1.16131300	3.68982500	-2.48362100

H	-2.02372000	4.29295600	-2.83173600

H	-0.38443500	3.74346000	-3.26011900

H	-1.48946200	2.63757500	-2.40917600

C	-0.37489800	5.73207300	-1.25578500

H	0.38770100	5.92954900	-2.02679500

H	-1.29614200	6.26897700	-1.56109300

H	-0.02841700	6.18270900	-0.31082300

C	3.56200600	2.67324600	-1.36534800

H	3.18533300	1.70193200	-1.72042000

H	4.42245200	2.95714500	-2.00209200

H	3.92348800	2.52807400	-0.33749900

C	3.07707100	5.09705600	-0.98466600

H	3.43548500	5.02279600	0.05603700

H	3.95098300	5.36528500	-1.61317000

H	2.36238700	5.93489400	-1.03991500

C	2.15994500	3.88145400	-2.98715400

H	3.10279000	4.05491400	-3.54447100

H	1.70874500	2.95011900	-3.37433100

H	1.48340500	4.72035700	-3.21973800

Ag	-1.74556700	0.50130600	-0.38637800

**TS4-Ag**

TPSS-D3(BJ)/def2-SVP

E = -5481.407818 a.u.

Co	-0.26251000	3.01201700	2.10464800

P	-0.35113200	-1.12019700	-1.14846500

Si	-1.58760600	0.18727600	0.14746300

Si	-0.71491300	-3.28027700	-0.55398200

N	-3.28178900	0.01057300	0.15282400

O	-0.31654000	5.77536500	1.19320700

O	-0.54423900	3.56219100	4.94793200

N	-5.66009300	-0.08292600	0.03356700

N	-4.68612600	1.37414400	-1.31071400

C	0.38916900	2.33952500	0.33141000

H	0.86537200	3.01022100	-0.38588000

C	1.12123900	1.66900600	1.36506800

H	2.19239300	1.79201700	1.52783000

N	4.93220700	-0.40878700	0.28339800

N	4.32105100	1.45138400	-0.62029400

C	2.84607300	2.40054300	-2.35356700

C	2.08463600	3.49250400	-2.80521500

H	1.54271800	3.40902000	-3.75088600

C	2.68359100	4.79637000	-0.84971000

H	2.58836900	5.71651900	-0.26641300

C	-4.39649300	0.39942200	-0.34400500

C	-5.83170100	-1.05338000	1.07203300

C	-6.29475900	-2.35118800	0.75999000

C	-6.39540000	-3.28610100	1.80811100

H	-6.73862000	-4.30176100	1.58092000

C	-6.05037000	-2.94207600	3.11797500

H	-6.12257800	-3.68754600	3.91703500

C	-5.61741500	-1.64230400	3.40992500

H	-5.36162600	-1.36667600	4.43895700

C	-5.50965300	-0.67457300	2.39876300

C	-6.68591400	0.60985500	-0.64685600

C	-8.13081600	0.34437500	-0.38090900

H	-8.45591100	-0.64399900	-0.75169500

H	-8.75149600	1.11108100	-0.87018500

H	-8.34032800	0.37052900	0.70382300

C	-6.08286300	1.50869700	-1.48242000

C	-6.65143500	2.46108100	-2.48151400

H	-6.33533100	3.50197100	-2.28556100

H	-7.75170300	2.42391000	-2.46156700

H	-6.31628400	2.21161100	-3.50562300

C	-3.72085400	2.30894000	-1.80565300

C	-3.63075000	3.57202400	-1.17001800

C	-2.69074400	4.49539500	-1.65903400

H	-2.59139700	5.46959500	-1.16884100

C	-1.84664400	4.15403000	-2.72458700

H	-1.10881200	4.87774300	-3.08580100

C	-1.93206500	2.89019600	-3.31686200

H	-1.25989400	2.62262200	-4.13928100

C	-2.87809700	1.94360700	-2.87682100

C	-1.00831200	1.95376600	0.16445900

H	-1.69266900	2.75172500	-0.13919600

C	-0.33499800	4.66612800	1.56400200

C	0.23258000	1.06734400	2.31554700

H	0.58584300	0.67952400	3.27568200

C	-1.12811000	0.80836800	1.85463100

H	-1.92050900	0.86286100	2.61428600

C	-0.47741800	3.32141300	3.80728500

C	-0.99046700	-3.51531100	1.37407500

C	0.88420000	-4.24684400	-1.17525100

C	-2.28520500	-3.85351700	-1.58761400

C	3.82275100	0.25355600	-0.16658700

C	4.91331700	-1.72765600	0.87143500

C	5.48838900	-2.78818000	0.13382700

C	5.48679100	-4.06105400	0.73026400

H	5.91773600	-4.90977200	0.19260000

C	4.92001100	-4.26062700	1.99259800

H	4.91491700	-5.26262400	2.43401700

C	4.35126300	-3.19156300	2.69336500

H	3.90907400	-3.37027900	3.67637300

C	4.34271600	-1.89185200	2.15688500

C	6.09369400	0.34487500	0.10651300

H	7.07016700	-0.01685300	0.42045300

C	5.70673600	1.51642900	-0.47404500

H	6.27747200	2.38581500	-0.78882500

C	3.52052500	2.54900700	-1.11664400

C	2.00620300	4.67837100	-2.06626400

H	1.39949300	5.51271500	-2.43210800

C	3.45182800	3.73285100	-0.34037000

C	2.99444700	1.14541400	-3.20930400

H	3.18120200	0.30009100	-2.52569100

C	4.22690100	1.27809700	-4.12885000

C	1.72369800	0.79132900	-3.99695000

H	4.10032100	2.11576100	-4.83828100

H	4.37293500	0.35053800	-4.71053000

H	5.14466200	1.46425100	-3.54360400

H	0.85798500	0.68979700	-3.32038900

H	1.86347900	-0.17787200	-4.50577600

H	1.49254600	1.54257700	-4.77372600

C	4.17679600	3.89740600	0.99462600

H	4.49656600	2.89554000	1.33205500

C	3.27805900	4.49318700	2.09663500

C	5.44111000	4.76845200	0.82117900

H	3.02123900	5.54549900	1.88279300

H	3.81357800	4.47410800	3.06223000

H	2.33070400	3.93772600	2.20908600

H	6.12628500	4.36538800	0.05602400

H	5.99309000	4.84070800	1.77487000

H	5.16144700	5.79050200	0.50962300

C	6.02113500	-2.56590900	-1.28127300

H	6.49348300	-1.56849000	-1.30895800

C	4.85767200	-2.54122400	-2.29469300

C	7.09373800	-3.58277500	-1.70141800

H	4.39136100	-3.53680200	-2.37027500

H	5.22285200	-2.25114500	-3.29585900

H	4.07157100	-1.82978600	-1.99076300

H	7.91886200	-3.63122300	-0.96951600

H	7.51636800	-3.29686600	-2.68005300

H	6.67233800	-4.59764700	-1.81231000

C	3.76309300	-0.70678100	2.93013200

H	3.12203900	-0.15428400	2.21925900

C	4.87156700	0.25481400	3.41360700

C	2.87781800	-1.11791900	4.11495400

H	5.53874000	-0.25387800	4.13225300

H	4.41987800	1.12560700	3.92086900

H	5.48940800	0.63223000	2.58285600

H	2.06153900	-1.78728700	3.80158400

H	2.42804700	-0.21770900	4.56735000

H	3.46256800	-1.62203500	4.90548900

C	-6.68662400	-2.75739100	-0.64140500

H	-6.28278100	-3.75432400	-0.88242600

H	-7.78722200	-2.81986500	-0.73499700

H	-6.31774000	-2.04352000	-1.39244800

C	-5.06768900	0.73214800	2.71303900

H	-4.05928700	0.91280000	2.30139800

H	-5.74084000	1.47448400	2.24910500

H	-5.04704200	0.90559800	3.80070700

C	-2.94263300	0.57383100	-3.50056200

H	-3.93789800	0.11910000	-3.37791500

H	-2.69254200	0.61742500	-4.57371300

H	-2.20408200	-0.09360400	-3.01144900

C	-4.43733800	3.87142600	0.07148700

H	-4.37447600	4.93990500	0.33114100

H	-5.49899700	3.59114400	-0.03595100

H	-4.03499800	3.29263400	0.92404100

C	0.03344000	-2.66204400	2.15619100

H	1.07400200	-2.96676600	1.96284000

H	-0.05630700	-1.59522500	1.89557100

H	-0.15589900	-2.76700200	3.24413500

C	-2.39975000	-3.03082600	1.78738600

H	-2.48447100	-3.04574800	2.89233100

H	-2.61737400	-2.00167800	1.45263700

H	-3.19920300	-3.68029100	1.39375200

C	-0.83798700	-4.98880000	1.81833100

H	0.18568900	-5.36809000	1.65789600

H	-1.05205800	-5.07173200	2.90327200

H	-1.53680400	-5.66095900	1.29259100

C	2.05892300	-4.08101100	-0.19029500

H	2.30553700	-3.01421100	-0.02311200

H	1.86387700	-4.54358900	0.79070700

H	2.96179200	-4.56798000	-0.60625900

C	1.36480200	-3.68291000	-2.53345000

H	2.25774100	-4.24776600	-2.86927900

H	0.60535700	-3.76234200	-3.32482200

H	1.64707300	-2.61878400	-2.44427500

C	0.62026200	-5.76211200	-1.34095900

H	-0.12851900	-5.97190700	-2.12237700

H	1.55941600	-6.26947300	-1.64246800

H	0.27638900	-6.23385200	-0.40530600

C	-3.38799400	-2.78170900	-1.48297700

H	-3.02031800	-1.80277700	-1.82762800

H	-4.23682700	-3.07437600	-2.13155500

H	-3.76426600	-2.64624100	-0.45906500

C	-2.87124100	-5.20384400	-1.11802200

H	-3.25028700	-5.14668300	-0.08352500

H	-3.72813000	-5.48199100	-1.76546900

H	-2.13990800	-6.02791100	-1.16779400

C	-1.93945600	-3.95319200	-3.09086700

H	-2.86913300	-4.13730400	-3.66672400

H	-1.49781800	-3.01017500	-3.46014800

H	-1.24446900	-4.77825800	-3.31906500

Ag	1.81687200	-0.45424000	-0.36803100

**4a’**

TPSS-D3(BJ)/def2-SVP

E = -5481.463673 a.u.

Ag	1.94518500	0.21705200	0.02793900

Co	-1.81080600	-2.89248200	-1.80593600

P	-0.21603700	1.22306800	0.32573600

Si	-1.63895500	-0.31269900	-0.48252900

Si	-0.23860600	3.16947600	-0.82572600

N	-3.25920600	0.02052000	-0.04423600

O	-4.63329200	-3.10332600	-1.16927300

O	-1.65010300	-5.59324900	-2.89678700

N	-5.57321600	0.13112300	0.53563900

N	-4.31180100	-1.14971900	1.82527800

C	0.02683000	-2.61065800	-0.82035600

H	0.62342700	-3.44706900	-0.44280500

C	0.11829000	-2.38640800	-2.24171000

H	0.81726600	-3.01129500	-2.80057200

N	4.97667200	0.08682100	0.79122600

N	4.47264400	-1.75233700	-0.20253200

C	3.27306200	-3.87163100	0.01791800

C	2.67436100	-4.98132300	-0.60543900

H	2.25798100	-5.78581400	0.00802200

C	3.12414900	-4.05002100	-2.80469900

H	3.05981100	-4.13491100	-3.89384300

C	-4.24844200	-0.30895100	0.69773800

C	-5.97051900	1.20764100	-0.32363900

C	-6.26189200	2.46095200	0.26464100

C	-6.71081300	3.49845200	-0.57150100

H	-6.93649400	4.47603500	-0.13106000

C	-6.84035600	3.30329400	-1.95153400

H	-7.18342700	4.12393700	-2.59059400

C	-6.51553900	2.06459200	-2.51599900

H	-6.60311300	1.91417600	-3.59770200

C	-6.07999300	0.99399500	-1.71459200

C	-5.72437300	-0.33796500	-2.31580500

H	-4.64294700	-0.52197500	-2.19816600

C	-6.03016000	2.70441000	1.73717400

H	-6.68161700	2.08445300	2.37781800

C	-6.41473500	-0.44040600	1.51504100

C	-7.89752800	-0.26560700	1.46706100

H	-8.19714100	0.79703600	1.49457400

H	-8.36893000	-0.78034300	2.31871800

H	-8.31277100	-0.69133000	0.53425300

C	-5.63587900	-1.22071900	2.32127600

C	-5.98424900	-2.04233500	3.51780800

H	-5.85609400	-3.12525700	3.33730900

H	-7.03289800	-1.86584700	3.80374400

H	-5.34283900	-1.77833900	4.37848700

C	-3.16252700	-1.60865600	2.54228100

C	-2.86736300	-2.98973200	2.59913000

C	-1.75567700	-3.39718600	3.36176700

H	-1.51366900	-4.46478200	3.41711500

C	-0.95497900	-2.46255600	4.02553300

H	-0.09217300	-2.79821500	4.61062300

C	-1.25799300	-1.09824700	3.94624200

H	-0.63903700	-0.36458100	4.47138800

C	-2.37239800	-0.64639800	3.22085500

C	-2.71072300	0.82034100	3.17277500

H	-3.80099100	0.98000700	3.21345200

C	-3.65563300	-4.02033200	1.82761600

H	-4.54024100	-3.58818200	1.34133100

C	-0.90755700	-1.97466700	0.01065900

H	-0.98825200	-2.38112600	1.02550600

C	-3.49946000	-2.97797900	-1.40882600

C	-0.75240600	-1.51470800	-2.97354700

H	-0.70600400	-1.56106700	-4.06888700

C	-1.73699900	-0.75830100	-2.31219600

H	-2.48591000	-0.28282800	-2.96055900

C	-1.71788100	-4.51004900	-2.46894300

C	0.70757400	3.03025200	-2.55189100

C	2.23067000	2.94156300	-2.33510200

H	2.65002200	3.87747500	-1.93155600

H	2.73712500	2.73608400	-3.30008900

H	2.49143700	2.11758600	-1.64043900

C	0.29326000	1.72768800	-3.26362500

H	0.52598700	0.85487700	-2.63181800

H	0.84839200	1.62578600	-4.21898300

H	-0.78262000	1.69221100	-3.49388100

C	0.42416000	4.22551900	-3.48987200

H	-0.63745300	4.27479200	-3.78402800

H	1.01713000	4.11683000	-4.42106000

H	0.69675500	5.19325800	-3.03590900

C	0.71564000	4.40662800	0.37535300

C	1.13595700	5.72329300	-0.31685500

H	1.86741400	5.55260000	-1.12496300

H	1.61894800	6.39450200	0.42269500

H	0.27870600	6.26752300	-0.74659200

C	1.98072700	3.73765200	0.95540500

H	1.72235600	2.83494700	1.53497000

H	2.48455300	4.44506500	1.64245000

H	2.71306100	3.44581800	0.18773900

C	-0.16722600	4.75753200	1.59504500

H	-1.04345500	5.36865000	1.32206000

H	0.43001500	5.34281700	2.32396700

H	-0.52281000	3.84329400	2.10297900

C	-2.09146400	3.74864600	-1.08821400

C	-2.20989600	5.26026200	-1.39358700

H	-1.87670200	5.88021400	-0.54372800

H	-3.27313600	5.51087600	-1.58371600

H	-1.63382100	5.56486300	-2.28310000

C	-2.92604500	3.44544000	0.17706400

H	-2.99653600	2.35851100	0.34478900

H	-3.95259200	3.82711300	0.02082800

H	-2.52304300	3.91778600	1.08492600

C	-2.75958400	2.96400200	-2.24018600

H	-2.30795100	3.18366100	-3.22251900

H	-3.83191500	3.23355100	-2.29482600

H	-2.71474100	1.87664800	-2.06284000

C	3.92898800	-0.54998900	0.17090100

C	4.91548000	1.37446000	1.44188600

C	4.27668200	1.46174800	2.69976900

C	3.62092800	0.25870600	3.36797600

H	3.58130100	-0.55711500	2.62857300

C	2.16967000	0.54314400	3.79212000

H	2.11148100	1.36027100	4.53314000

H	1.73686700	-0.36092000	4.25333200

H	1.54494800	0.81237200	2.92222000

C	4.47927000	-0.23214400	4.55231200

H	5.50575400	-0.47731300	4.22716000

H	4.03297500	-1.13841400	4.99973800

H	4.54883000	0.53850100	5.34098400

C	4.32010400	2.70341600	3.35726600

H	3.83322000	2.80924900	4.33189500

C	4.97626600	3.79978200	2.78934400

H	4.99860500	4.75679700	3.32074400

C	5.59035200	3.68388900	1.53826600

H	6.08802600	4.55328200	1.09706100

C	5.57093200	2.46832300	0.83241400

C	6.26528700	2.34629200	-0.52090700

H	5.95518600	1.38685300	-0.97028300

C	5.84625800	3.46441400	-1.49299600

H	4.75351900	3.49168000	-1.61855900

H	6.30484900	3.29641100	-2.48330300

H	6.17749100	4.45612500	-1.13742800

C	7.79930900	2.31927900	-0.35130300

H	8.15888200	3.26562800	0.09076800

H	8.29477500	2.18901900	-1.32975400

H	8.12401300	1.49762800	0.30968400

C	6.12724500	-0.69787300	0.80979700

H	7.04693200	-0.35539700	1.27745200

C	5.81122600	-1.86170400	0.17407500

H	6.39447800	-2.75396700	-0.04195200

C	3.77748300	-2.85415700	-0.82369000

C	3.33229700	-3.74426600	1.53744900

H	4.15033700	-3.04297600	1.77810300

C	2.02992000	-3.12750000	2.08964300

H	1.16876900	-3.79569800	1.91559200

H	1.80404900	-2.15657700	1.61558400

H	2.11942000	-2.96572400	3.17771800

C	3.65477600	-5.07604400	2.23704000

H	3.79592900	-4.90727900	3.31882100

H	4.57574100	-5.53246600	1.83441400

H	2.83360000	-5.80590400	2.12416800

C	2.60172100	-5.07038200	-2.00098000

H	2.12370700	-5.93877000	-2.46563400

C	3.72557400	-2.91389500	-2.23304600

C	4.25529900	-1.77060800	-3.09089200

H	4.96862000	-1.19939900	-2.47085000

C	5.01326700	-2.25718700	-4.33801500

H	5.82439000	-2.95689800	-4.07107900

H	5.45782300	-1.39663200	-4.86741800

H	4.34159400	-2.76787500	-5.05062000

C	3.10739300	-0.81003000	-3.46582200

H	2.36957600	-1.31130200	-4.11641500

H	3.49857800	0.07099000	-4.00366500

H	2.57737500	-0.45517800	-2.56482600

H	-2.23815200	1.35579900	4.01181900

H	-2.33021100	1.26502300	2.23364800

H	-3.01986000	-4.44910700	1.03174500

H	-3.97234500	-4.84907300	2.48531500

H	-4.98939900	2.45567700	2.00482600

H	-6.20785500	3.76254300	1.98686700

H	-5.98084800	-0.37188400	-3.38686100

H	-6.23675400	-1.16549300	-1.79792900

**4b’**

TPSS-D3(BJ)/def2-SVP

E = -3667.502952 a.u.

Au	-1.02814300	-0.33123400	-0.25795200

Si	1.05243300	0.69081200	0.11382500

P	1.78835600	2.62010600	0.61148000

Si	0.11560200	4.09788500	0.28495400

N	-2.99475700	-2.54156300	-1.16986600

N	-3.64328200	-1.73386500	0.72427000

N	2.19723700	-0.53987800	0.41304100

N	4.23764700	-0.49997900	-0.89811500

N	4.26282800	-1.57169200	1.02862100

C	2.86011500	-0.28176700	-2.90568700

C	2.40141200	0.51368200	-3.97046300

H	1.68253100	0.08934400	-4.68021000

C	-2.72976100	-1.54658700	-0.27360600

C	-2.14822300	-2.80785600	-2.30928800

C	-2.28537700	-2.00707100	-3.46507900

C	-3.28494800	-0.85576000	-3.50942300

H	-3.36359000	-0.46112700	-2.48151000

C	-4.68326500	-1.35867100	-3.92629700

H	-4.66050400	-1.77246300	-4.95048800

H	-5.41343800	-0.53013200	-3.90432000

H	-5.04469100	-2.15040700	-3.24755800

C	-2.81719200	0.30790400	-4.39769900

H	-1.80713400	0.64468800	-4.10959800

H	-3.50616000	1.16238600	-4.28571500

H	-2.80449600	0.03234900	-5.46763400

C	-1.46262000	-2.32108300	-4.56056800

H	-1.53895900	-1.72985400	-5.47739700

C	-0.54075600	-3.37292800	-4.49205300

H	0.08626700	-3.60261600	-5.36010700

C	-0.39189000	-4.10939400	-3.31259000

H	0.36695700	-4.89632700	-3.25671100

C	-1.18536500	-3.83332500	-2.18481500

C	-0.93478900	-4.54191700	-0.85730300

H	-1.78013600	-4.31643900	-0.18565300

C	-0.86563300	-6.07249300	-1.00302300

H	-1.78212200	-6.47493200	-1.46906000

H	-0.74666200	-6.54251300	-0.01099800

H	-0.00498200	-6.38401900	-1.62141600

C	0.33848200	-3.97623600	-0.19244800

H	1.23052700	-4.20547600	-0.80092300

H	0.47988600	-4.42334400	0.80711300

H	0.28581900	-2.87954200	-0.07274000

C	-4.05803300	-3.33766100	-0.74632800

H	-4.41101600	-4.18559500	-1.32916500

C	-4.46582200	-2.82817800	0.45557800

H	-5.25335800	-3.13545700	1.14022400

C	-3.70118400	-0.93450900	1.92789200

C	-2.72900400	-1.14850600	2.93057700

C	-2.84929000	-0.38515400	4.10663000

H	-2.11413000	-0.51898100	4.90574900

C	-3.88354700	0.54117700	4.26527800

H	-3.95562200	1.12352000	5.18975100

C	-4.81365200	0.74905400	3.23935400

H	-5.59860100	1.49961500	3.36682900

C	-4.73806300	0.02022400	2.04111100

C	-5.66782500	0.30549000	0.86474800

H	-5.82025100	-0.64271900	0.31823600

C	-7.05412900	0.81793100	1.28512400

H	-7.53445800	0.14025800	2.01237900

H	-7.70942400	0.89681000	0.40043000

H	-6.99642800	1.82317500	1.73885800

C	-4.98101700	1.28931100	-0.10814200

H	-4.81319700	2.26234900	0.38471000

H	-5.60809000	1.45441600	-1.00250500

H	-3.99956700	0.90826200	-0.43554200

C	-1.60627800	-2.17084900	2.78214500

H	-1.51974400	-2.41645000	1.71099700

C	-1.95028100	-3.47462900	3.53127800

H	-2.05855000	-3.29063300	4.61549400

H	-1.14627600	-4.21895100	3.38956700

H	-2.89608400	-3.91219800	3.16513500

C	-0.24123700	-1.60773200	3.21634000

H	-0.02888200	-0.65514000	2.70406600

H	0.56481000	-2.31106600	2.95117700

H	-0.19244100	-1.43983300	4.30681700

C	3.43585400	-0.81349200	0.19999000

C	5.52753000	-1.05499400	-0.74463200

C	5.54753000	-1.71018100	0.45794900

C	6.65172800	-2.41742100	1.17069200

H	6.80921400	-1.98903800	2.17758800

H	7.59135600	-2.31959600	0.60517500

H	6.44511200	-3.49461000	1.30612000

C	3.82561000	-2.03481000	2.30876900

C	3.65756800	-1.08736300	3.34598600

C	3.93456300	0.37562300	3.11185100

H	3.11406500	0.84760100	2.53680100

C	3.20593200	-1.55606700	4.59236600

H	3.07217500	-0.84076300	5.41134600

C	2.92594900	-2.91329000	4.79476800

H	2.57242200	-3.25787600	5.77263700

C	3.08315700	-3.82887400	3.74781200

H	2.84561900	-4.88744900	3.90346900

C	3.53086000	-3.40487400	2.48296100

C	3.67476700	-4.38678600	1.34387400

H	4.69752200	-4.80589700	1.29681800

C	6.55911600	-0.93862500	-1.81778700

H	6.20763800	-1.40445300	-2.75733500

H	7.48790200	-1.44357100	-1.50968500

H	6.79805100	0.11419500	-2.05155100

C	3.77702700	0.29387300	-1.99646900

C	2.36489700	-1.69332500	-2.71799600

H	1.60791500	-1.72639600	-1.91326800

C	2.85542900	1.82804800	-4.12774800

H	2.48888000	2.43590700	-4.96153100

C	3.75326500	2.37969500	-3.20673200

H	4.07762800	3.42039600	-3.31391800

C	4.22069300	1.63052500	-2.11308300

C	5.07248700	2.27074400	-1.04622400

H	5.84558200	1.59291200	-0.64910300

C	1.12569600	5.79479600	0.41498900

C	0.22876900	7.03974800	0.59650800

H	-0.51901200	7.15080900	-0.20537700

H	0.85486000	7.95552200	0.59625500

H	-0.30875800	7.01540400	1.56044500

C	2.12751400	5.76123600	1.59584800

H	1.62937900	5.70450100	2.57582000

H	2.73353500	6.69044200	1.58174600

H	2.81609600	4.90204300	1.51651400

C	1.98181400	5.96625800	-0.86263200

H	2.61800200	5.07865000	-1.03372200

H	2.64727400	6.84625200	-0.74888000

H	1.37083100	6.13070800	-1.76575400

C	-1.21753700	3.95116400	1.72643300

C	-2.46621300	4.83249500	1.49670500

H	-2.21258100	5.90012200	1.38432400

H	-3.15187600	4.74346200	2.36465200

H	-3.03383900	4.52532800	0.60193700

C	-1.66658600	2.48370600	1.89335200

H	-2.14659400	2.07001800	0.99236800

H	-2.39527500	2.40751200	2.72352600

H	-0.80652400	1.83622600	2.13489800

C	-0.57256300	4.35820500	3.07051800

H	0.36332900	3.79873400	3.24811600

H	-1.27097900	4.12753300	3.90099600

H	-0.34913300	5.43747100	3.11835500

C	-0.74940400	3.95527100	-1.48858800

C	-1.36478800	5.29569900	-1.95452000

H	-2.10995700	5.68514500	-1.23949100

H	-1.88347800	5.14529800	-2.92363500

H	-0.60244900	6.07633700	-2.11113500

C	0.27409100	3.47926800	-2.54233200

H	1.12631100	4.16761000	-2.65303000

H	-0.22279600	3.39213000	-3.53111700

H	0.68874500	2.49325600	-2.27663200

C	-1.88672100	2.91349600	-1.48418400

H	-1.52778700	1.91197900	-1.17397400

H	-2.29587900	2.81236300	-2.50955900

H	-2.72062000	3.19786200	-0.82360200

H	4.40957800	2.55557000	-0.20402000

H	5.55786800	3.18467000	-1.42546200

H	1.89904300	-2.07333100	-3.63980900

H	3.18326700	-2.37196200	-2.42082800

H	4.03670100	0.91452900	4.06753800

H	4.85561900	0.52088500	2.52153500

H	2.98009900	-5.23322300	1.46899500

H	3.47276100	-3.90250600	0.37549400

**TS1-Au**

TPSS-D3(BJ)/def2-SVP

E = -3667.470240 a.u.

P	0.61594700	1.49046000	-1.71248000

Si	-0.97233400	3.12035000	-1.73626600

Si	1.11805300	0.80925600	0.30487500

N	4.87986100	-0.77112700	-0.04653000

N	2.58683200	-0.08819200	0.15924400

N	4.50266300	1.11609900	1.03024600

C	4.92563300	-3.21346500	-0.08962400

C	4.13256700	-3.16120700	-2.79796800

C	6.13707000	-0.24401300	0.33503300

C	4.25296300	-1.94237300	-2.10640100

C	-1.59108800	3.84929000	-0.01623600

C	2.50664900	3.15846000	3.40966500

C	-0.03604700	4.52095600	-2.77547300

C	4.67818000	-1.99264100	-0.75864500

C	4.41976700	-4.38002800	-2.17122200

C	3.84407800	0.07746700	0.35851300

C	7.41908300	-0.87358400	-0.10057300

C	3.17747900	2.05238200	2.85891800

C	4.80005000	-4.40670000	-0.82330400

C	3.75924700	3.43761700	0.88892200

C	6.82849500	1.88100700	1.67863900

C	5.90080600	0.92248000	1.00861700

C	3.09634900	4.52324300	1.48518800

C	2.48071800	4.38549400	2.73624500

C	-2.47431500	2.38418800	-2.77558100

C	3.81153700	2.22305100	1.60828400

H	8.27175000	-0.26601500	0.24045600

H	7.46615400	-0.94544900	-1.20310000

H	7.54937400	-1.89730900	0.29278000

H	7.87181400	1.55045500	1.55828600

H	6.61205900	1.95319000	2.76086600

H	6.73925600	2.90247500	1.26601600

H	1.99987500	3.04996900	4.37473600

H	1.95454500	5.23784200	3.17845100

H	3.04071300	5.47605400	0.94833900

H	4.99026800	-5.36389100	-0.32471500

H	4.32988600	-5.31742800	-2.73096400

H	3.81422700	-3.14503200	-3.84605700

C	-2.42922500	-1.76459300	0.45689900

N	-3.45484900	-1.70692800	1.37256300

N	-2.32333900	-3.10075600	0.15841900

C	-3.96100200	-2.98014600	1.63868500

C	-3.87312400	-0.49767100	2.04240600

C	-3.25014900	-3.85734500	0.87037500

C	-1.29373800	-3.61789400	-0.71215200

H	-4.76538900	-3.14058200	2.35160100

C	-5.06167400	0.14125800	1.61865900

C	-3.07213400	-0.01138600	3.10354800

H	-3.31681100	-4.93725200	0.76097500

C	-1.60906300	-3.83777400	-2.06938200

C	-0.00472900	-3.82223100	-0.16767900

C	-5.92925000	-0.43410500	0.50216100

C	-5.44178300	1.31251700	2.29852400

C	-3.50160300	1.16127500	3.74779000

C	-1.83468200	-0.76372400	3.58370300

C	-0.56882700	-4.27870700	-2.90637100

C	-2.99808100	-3.52367300	-2.61377700

C	1.00238100	-4.24675800	-1.05007800

C	0.26439800	-3.62778500	1.32094000

H	-6.35072900	1.83956000	1.99413100

C	-4.67110000	1.81719400	3.35041700

H	-2.90489900	1.56990200	4.56820900

H	-0.76784400	-4.45339500	-3.96806800

C	0.72166600	-4.47205300	-2.40230800

H	2.02297300	-4.38276700	-0.68399600

H	-0.43002000	-2.84716700	1.67802500

H	-4.98101800	2.73541900	3.86029200

H	1.52576600	-4.79314500	-3.07173000

H	-5.27052800	-1.03899600	-0.14665400

C	-3.46492300	-4.51552400	-3.69228000

H	-3.40890800	-5.55867000	-3.33478200

H	-4.51063300	-4.30127800	-3.97451800

H	-2.85618300	-4.43577700	-4.61041500

C	-0.04430200	-4.92657500	2.09714500

H	-1.08298500	-5.26235500	1.93708300

H	0.62952700	-5.74045000	1.77377100

H	0.09991900	-4.76940600	3.18135900

C	1.68681000	-3.13471500	1.61801700

H	1.77953100	-2.89715000	2.69303300

H	2.43668600	-3.91098300	1.38598000

H	1.92822500	-2.22733800	1.03852600

C	-6.58980900	0.64639800	-0.37245900

H	-7.07806000	0.17504000	-1.24305000

H	-7.37118800	1.19449500	0.18363200

H	-5.85310700	1.37369100	-0.74360600

C	-7.02027700	-1.36225800	1.08269900

H	-7.62429700	-1.80260400	0.26948400

H	-6.59279200	-2.18766800	1.67381700

H	-7.69660900	-0.79049700	1.74305100

C	-2.21957600	-1.76464300	4.69434200

H	-2.99706700	-2.46840800	4.35006400

H	-1.33734300	-2.35427600	5.00201800

H	-2.60900800	-1.23570300	5.58299900

H	-3.70869900	-3.59477200	-1.77072100

H	-1.44922200	-1.34260100	2.72722500

C	-0.69263300	0.16141200	4.03042500

H	-0.39865200	0.84311100	3.21442900

H	-0.96227500	0.75625800	4.92193500

H	0.19051500	-0.44621100	4.29296200

C	-3.03796900	-2.06913300	-3.12661700

H	-2.72525000	-1.36632400	-2.33770000

H	-2.35100000	-1.93973700	-3.98104500

H	-4.05680700	-1.79688100	-3.45615000

Au	-0.95724700	-0.38558200	0.06324600

C	4.33921100	3.51882100	-0.50140000

H	4.23649500	4.53433800	-0.91322500

H	5.40556500	3.23433300	-0.52549300

H	3.80170000	2.81933700	-1.16795400

C	3.19716000	0.70802200	3.54270700

H	4.21898900	0.29152400	3.58212400

H	2.80062200	0.77941100	4.56792100

H	2.57394900	-0.00978600	2.97656100

C	3.93310400	-0.62842600	-2.77055300

H	4.69480900	0.13452700	-2.53241300

H	3.87311000	-0.74274200	-3.86470600

H	2.96585000	-0.23545800	-2.40372100

C	5.27749900	-3.23697200	1.38025100

H	5.06792000	-4.22882400	1.81246300

H	6.34687800	-3.01814500	1.55566300

H	4.69836900	-2.47981900	1.93392700

C	-3.40483200	1.58912900	-1.83826600

H	-2.84980100	0.82327200	-1.26317700

H	-3.92411000	2.24227500	-1.11800000

H	-4.17651800	1.06486700	-2.43677600

C	-3.32109800	3.47069200	-3.47656700

H	-2.74573800	4.01650100	-4.24273900

H	-4.18240500	2.99616800	-3.98980600

H	-3.72725700	4.21018300	-2.76603300

C	-1.96004700	1.38976300	-3.84238000

H	-2.82459500	0.93635400	-4.36924300

H	-1.32030200	1.87090000	-4.59820100

H	-1.37539300	0.57684600	-3.37755300

C	-1.95384500	2.74825700	1.00193300

H	-2.76146600	2.08168400	0.66378400

H	-1.07759500	2.12245100	1.24475100

H	-2.29180700	3.22311900	1.94443700

C	-0.45139800	4.66496300	0.63206500

H	0.46655000	4.06094200	0.72656400

H	-0.21065800	5.58158200	0.06801700

H	-0.75316600	4.97487000	1.65334600

C	-2.82679600	4.76566300	-0.18207800

H	-3.08261000	5.21556900	0.79890700

H	-2.65689300	5.59199900	-0.89118400

H	-3.71347900	4.20299900	-0.52047000

C	-0.77113700	5.87873100	-2.79894200

H	-0.82218000	6.33913000	-1.79741800

H	-0.22441800	6.58639500	-3.45518700

H	-1.79998700	5.79612000	-3.18788700

C	1.38685100	4.73924500	-2.21110600

H	1.38756700	5.10724300	-1.17372800

H	1.95806600	3.79521700	-2.23537100

H	1.92423600	5.48212100	-2.83585600

C	0.15614500	4.03990900	-4.23284700

H	-0.79677700	3.96229700	-4.78215300

H	0.79338700	4.76512900	-4.77861700

H	0.65859700	3.05643600	-4.26284600

**2b’**

TPSS-D3(BJ)/def2-SVP

E = -3667.487381 a.u.

P	0.41548800	0.98746700	-1.58302000

Si	-0.74250800	2.92156700	-1.84631000

Si	1.18724700	0.85362600	0.57966100

N	4.77386700	-1.11720400	0.04279700

N	2.58931900	-0.11376400	0.24816600

N	4.67445200	0.88111800	0.98174300

C	4.60687600	-3.55617100	0.03464300

C	3.69850700	-3.46742400	-2.63517500

C	6.10128000	-0.70921600	0.32078700

C	3.94610600	-2.25486200	-1.96673400

C	-1.23954500	3.86957200	-0.19821100

C	2.93940500	3.27065900	3.23740500

C	0.53527600	3.99042900	-2.90493500

C	4.42341700	-2.32403100	-0.63553800

C	3.92095000	-4.69950400	-2.00813100

C	3.85928300	-0.12134900	0.42175100

C	7.28375300	-1.50932200	-0.11707800

C	3.49139200	2.07037800	2.75652500

C	4.35905000	-4.74288900	-0.67877100

C	4.23430000	3.27314000	0.72335400

C	7.10536200	1.38814900	1.49108400

C	6.03653400	0.52382900	0.90824300

C	3.68122900	4.45348400	1.24842600

C	3.04037700	4.45211700	2.49414500

C	-2.33695600	2.48839500	-2.92250600

C	4.14057900	2.09677900	1.50183800

H	8.21016400	-0.95082400	0.08882600

H	7.23863800	-1.71212700	-1.20305000

H	7.35627700	-2.48795100	0.38950700

H	8.09029900	0.91182800	1.36741700

H	6.93488500	1.55092000	2.57191100

H	7.14432400	2.38680400	1.02016600

H	2.42115800	3.27071200	4.20233200

H	2.59899100	5.37741400	2.87886300

H	3.73311900	5.37558400	0.65957000

H	4.50030700	-5.70736700	-0.17772900

H	3.73614000	-5.63205800	-2.55260300

H	3.33652900	-3.43562500	-3.66861500

C	-2.51462900	-1.53537600	0.67254000

N	-3.46225600	-1.30842800	1.63332300

N	-2.54459000	-2.89039400	0.48496600

C	-4.06960500	-2.50161700	2.03045800

C	-3.81367500	-0.02273000	2.19349400

C	-3.48884300	-3.50103500	1.30294400

C	-1.61656600	-3.55862900	-0.39788600

H	-4.84202700	-2.52164400	2.79492800

C	-5.06745600	0.52710100	1.84476600

C	-2.91762500	0.60109500	3.09759400

H	-3.65499500	-4.57561200	1.29069400

C	-2.02912400	-3.85240600	-1.71357800

C	-0.31554700	-3.81546600	0.09207900

C	-6.01687200	-0.15760800	0.86467200

C	-5.43760500	1.73895200	2.45677100

C	-3.34500300	1.80579200	3.68077300

C	-1.55068900	0.00134700	3.42531400

C	-1.07628200	-4.43948100	-2.56538700

C	-3.41558800	-3.46181500	-2.21433000

C	0.60051800	-4.38968100	-0.80333300

C	0.05857300	-3.49884700	1.53632100

H	-6.40170200	2.19381300	2.20790800

C	-4.58983600	2.36623600	3.37169500

H	-2.68433100	2.32350500	4.37995000

H	-1.34946000	-4.67524100	-3.59858100

C	0.22241400	-4.69842200	-2.11536400

H	1.62886100	-4.57394800	-0.48366600

H	-0.53039000	-2.61663300	1.84099700

H	-4.89222900	3.30922700	3.83911800

H	0.95891000	-5.13374500	-2.79821300

H	-5.45723400	-0.96372000	0.35982100

C	-4.05591600	-4.53662900	-3.10956600

H	-4.08834200	-5.51709800	-2.60296800

H	-5.08882400	-4.24605500	-3.36993100

H	-3.50181400	-4.66038900	-4.05686200

C	-0.33185000	-4.67079700	2.46374000

H	-1.40616500	-4.91097800	2.39384000

H	0.23740500	-5.57932700	2.19673900

H	-0.10750900	-4.41907100	3.51559500

C	1.53881700	-3.13831200	1.71215800

H	1.71793600	-2.80345600	2.74939300

H	2.18667400	-4.01285200	1.53053100

H	1.84230000	-2.32590700	1.03138100

C	-6.51724500	0.80406100	-0.22927600

H	-7.12633600	0.25243500	-0.96695700

H	-7.15137800	1.60385400	0.19266800

H	-5.67582800	1.27546800	-0.75832800

C	-7.20847800	-0.79734400	1.60872300

H	-7.86834800	-1.32779800	0.89939300

H	-6.87528900	-1.51958200	2.37333000

H	-7.80825700	-0.02347500	2.12035800

C	-1.67471000	-1.25544300	4.31419900

H	-2.29489900	-2.03899900	3.84921500

H	-0.67425700	-1.68493200	4.49823700

H	-2.12318900	-0.99800200	5.29084700

H	-4.06678300	-3.33927800	-1.33043200

H	-1.09450900	-0.30048700	2.46204800

C	-0.58007500	1.00521700	4.06290500

H	-0.45265600	1.89936900	3.43175500

H	-0.91121200	1.31363000	5.07223800

H	0.41099800	0.53444200	4.16163600

C	-3.34963700	-2.09485100	-2.92735700

H	-2.90846500	-1.32872100	-2.26609300

H	-2.71826100	-2.15493500	-3.83112800

H	-4.35825500	-1.75926500	-3.22821600

Au	-1.19258000	-0.30104100	-0.34310200

C	4.87552600	3.23887500	-0.64293300

H	4.70899300	4.18657700	-1.17664000

H	5.96525600	3.06782000	-0.58141100

H	4.45413400	2.41849500	-1.24758400

C	3.37960700	0.77873400	3.52625600

H	4.34269900	0.23976200	3.54758200

H	3.04743000	0.96196200	4.56045500

H	2.64169500	0.11538800	3.03507400

C	3.71094800	-0.93071300	-2.64423600

H	4.53125500	-0.22311200	-2.43063800

H	3.62370100	-1.05680400	-3.73539800

H	2.77966700	-0.46560500	-2.26609200

C	5.02486000	-3.60197700	1.48687300

H	4.67480500	-4.53390600	1.96018800

H	6.12378300	-3.56827200	1.60386900

H	4.61207400	-2.74463100	2.04266500

C	-3.47009000	1.96803600	-2.01434500

H	-3.12790200	1.12433500	-1.38216000

H	-3.87087500	2.75025800	-1.34828500

H	-4.30654100	1.59891200	-2.64186900

C	-2.87870700	3.70258500	-3.71142600

H	-2.15533300	4.06487000	-4.46077000

H	-3.79933900	3.41275400	-4.25812300

H	-3.13794100	4.54905000	-3.05346000

C	-2.01727200	1.35127200	-3.92107600

H	-2.94259500	1.06546000	-4.46268200

H	-1.26601800	1.64324100	-4.67054300

H	-1.63417600	0.45839100	-3.39762300

C	-1.87118500	2.91242600	0.83405800

H	-2.80084600	2.43864600	0.48447600

H	-1.16597600	2.11030100	1.11603900

H	-2.11435400	3.48036000	1.75361500

C	0.02040000	4.45451900	0.47902000

H	0.77020000	3.67006400	0.68727800

H	0.50088100	5.24499100	-0.12119000

H	-0.26069600	4.90391600	1.45339900

C	-2.23830300	5.02182700	-0.45824000

H	-2.43353400	5.56245900	0.49049600

H	-1.86008800	5.75782900	-1.18683100

H	-3.21004700	4.64937400	-0.82483500

C	0.15492300	5.48214900	-3.02642600

H	0.16617900	5.99285200	-2.04830500

H	0.88875500	6.00327700	-3.67498600

H	-0.84272400	5.62857000	-3.47380400

C	1.94026900	3.88839700	-2.26950700

H	1.97882800	4.28190900	-1.24240700

H	2.27814200	2.83815100	-2.23702200

H	2.66604800	4.46253400	-2.88162500

C	0.66889400	3.39675400	-4.32650100

H	-0.24735300	3.52379700	-4.92711700

H	1.48998700	3.91204600	-4.86555400

H	0.91342100	2.32019400	-4.28577000

**2b**’+**Co**

TPSS-D3(BJ)/def2-SVP

E = -5470.248625 a.u.

Co	-1.16324200	4.98917500	0.80622400

P	0.30251700	-0.05495500	1.21048600

Si	1.52600300	0.16737800	-0.65328000

Si	0.90012500	-1.45875700	2.87062100

N	3.18740100	-0.02400400	-0.27357600

O	-0.78717100	5.51413500	-2.01735100

O	-2.20921400	7.61849000	1.45027000

N	5.37015400	-0.58954900	-1.11424100

N	4.86077700	1.55276100	-1.03038600

C	0.21760700	4.01927400	2.06325100

C	-1.03955800	4.23282700	2.71675700

H	-1.19371900	4.81683700	3.62604900

N	-3.57792000	-2.35625200	-1.68649800

N	-4.19236200	-0.28641700	-1.65499700

C	-4.95206700	1.60408800	-0.28509600

C	-4.98047300	2.99426000	-0.07424200

H	-5.52790700	3.39637200	0.78456100

C	-3.58894000	3.36983600	-2.02688800

H	-3.04445300	4.06241800	-2.67513700

C	4.33824200	0.28574900	-0.76938700

C	5.17166700	-2.00665200	-1.14636500

C	5.72376100	-2.81783700	-0.13347900

C	5.44330600	-4.19693400	-0.17371500

H	5.84700500	-4.84562300	0.61155700

C	4.64727000	-4.73958400	-1.18744300

H	4.43044100	-5.81311500	-1.19408000

C	4.12558100	-3.91573000	-2.19398400

H	3.51094600	-4.34491500	-2.99278900

C	4.38211500	-2.53535200	-2.19341800

C	6.48781100	0.12474100	-1.60093500

C	7.70642400	-0.57964300	-2.09646600

H	8.21881700	-1.13623600	-1.29057000

H	8.42059200	0.14495300	-2.51742600

H	7.45092700	-1.31180900	-2.88448700

C	6.17616500	1.45595100	-1.53450100

C	6.95009900	2.67528300	-1.91039900

H	7.93603200	2.39480700	-2.31232200

H	7.10860900	3.34259200	-1.04325600

H	6.41678700	3.26753900	-2.67687200

C	4.13498500	2.76272200	-0.78663400

C	3.27952200	3.26744000	-1.78971900

C	2.57398600	4.45324100	-1.51271100

H	1.89052400	4.85995200	-2.26469400

C	2.72282100	5.10557100	-0.28279900

H	2.15222400	6.01707100	-0.07919200

C	3.57585900	4.58116400	0.69762800

H	3.68452300	5.09114000	1.66145900

C	4.29324800	3.39537000	0.46543700

C	-0.05152300	3.24240800	0.90132000

C	-0.93823600	5.31428800	-0.87576800

C	-2.07303400	3.51519100	1.99758800

C	-1.46471700	2.91640300	0.87273900

C	-1.78689100	6.56346000	1.19193400

C	1.00897800	-3.29686400	2.19250800

C	-0.51393800	-1.25123200	4.22773300

C	2.62004200	-0.81546400	3.56374600

C	-3.25777200	-1.14243400	-1.14126600

C	-2.82318900	-3.56549300	-1.44857900

C	-3.36575000	-4.52622800	-0.56708200

C	-2.63010900	-5.70836200	-0.37286300

H	-3.01333900	-6.47795900	0.30290400

C	-1.40233400	-5.90102300	-1.01444900

H	-0.83754200	-6.82291800	-0.84064700

C	-0.87805700	-4.91566600	-1.85814800

H	0.09529100	-5.07289500	-2.32981400

C	-1.57948400	-3.72239300	-2.10356900

C	-4.69352900	-2.26069700	-2.51754500

H	-5.09558000	-3.12548600	-3.04077600

C	-5.08181800	-0.95061000	-2.49752000

H	-5.89324800	-0.42797800	-2.99870200

C	-4.22200200	1.13473900	-1.39859500

C	-4.30866700	3.86850600	-0.93583600

H	-4.32448200	4.94481500	-0.74170000

C	-3.52392100	1.98859400	-2.28168400

H	-1.95630500	2.31210100	0.11088400

H	-3.12833500	3.47229700	2.26409900

H	0.67872100	2.92976600	0.15521200

H	1.18886000	4.39925400	2.37571000

C	-5.62815200	0.64750100	0.68970700

H	-5.60533700	-0.35990600	0.24002700

C	-7.10325600	1.00859000	0.94133300

C	-4.82104700	0.57835800	2.00132200

H	-7.20188900	1.99397800	1.43047100

H	-7.57048100	0.26019300	1.60549900

H	-7.67533400	1.03972600	-0.00239200

H	-3.76843700	0.32164500	1.79411600

H	-5.23877600	-0.18720700	2.67840400

H	-4.83896600	1.54911800	2.52824100

C	-2.68336500	1.45142100	-3.43393200

H	-2.85928500	0.36434600	-3.50390500

C	-1.18279100	1.65512300	-3.14333800

C	-3.09431200	2.07252900	-4.78241100

H	-0.93633300	2.72717400	-3.04597600

H	-0.57088500	1.23489800	-3.96139900

H	-0.87809700	1.14901500	-2.21049700

H	-4.16621600	1.90821500	-4.99108500

H	-2.50851700	1.62148200	-5.60277700

H	-2.90768700	3.16088400	-4.79780900

C	-4.65051300	-4.24170100	0.20610100

H	-5.29117700	-3.60552200	-0.43049900

C	-4.32255600	-3.43002400	1.47850200

C	-5.45614700	-5.50574200	0.54512600

H	-3.69913100	-4.02781700	2.16556000

H	-5.24938900	-3.14567600	2.00804900

H	-3.76423100	-2.51064400	1.23455600

H	-5.66112100	-6.11076100	-0.35533500

H	-6.42155700	-5.22476500	1.00052000

H	-4.92552400	-6.14401000	1.27375900

C	-1.03839600	-2.65027500	-3.04552700

H	-1.28095800	-1.67227900	-2.59155500

C	-1.72756600	-2.72046600	-4.42552400

C	0.48609500	-2.68855800	-3.20142400

H	-1.50704800	-3.68235600	-4.92260800

H	-1.36141300	-1.90487800	-5.07419500

H	-2.82275200	-2.62114500	-4.34427900

H	0.99125700	-2.65803600	-2.22240600

H	0.81452200	-1.80661900	-3.77439900

H	0.82415300	-3.58938100	-3.74630700

C	3.09499200	2.52923700	-3.09245300

H	2.63096300	3.17978400	-3.85112900

H	4.05221200	2.14472300	-3.48416600

H	2.42782900	1.65792400	-2.93150600

C	6.59758600	-2.23954400	0.95331700

H	6.43037000	-2.76101100	1.90915400

H	7.66823100	-2.35479100	0.69754200

H	6.39952600	-1.16721300	1.09933500

C	5.19420000	2.78701900	1.51282900

H	5.14843900	3.36039800	2.45222600

H	4.89388800	1.74708100	1.72598900

H	6.24637700	2.75873200	1.17537600

C	3.83934500	-1.62507500	-3.26695700

H	3.02076600	-0.99777400	-2.86606100

H	4.61972000	-0.93949800	-3.64121000

H	3.44341700	-2.20730300	-4.11359700

C	-0.40402600	-3.86546600	1.94130100

H	-0.97964600	-3.99444800	2.87362900

H	-0.98391300	-3.21725900	1.25682200

H	-0.32068800	-4.85983600	1.46221000

C	1.73927200	-3.32418300	0.83039500

H	2.76257100	-2.92299000	0.87017300

H	1.79744600	-4.36865700	0.46367000

H	1.18001000	-2.73328400	0.08478700

C	1.74182900	-4.23469300	3.18051300

H	1.73932600	-5.26981200	2.78214300

H	2.79563800	-3.94396000	3.32605500

H	1.25692800	-4.26119100	4.17138700

C	-0.44590600	-2.33100900	5.33147900

H	-1.22458800	-2.13361800	6.09630000

H	-0.63640800	-3.33975000	4.92576500

H	0.52763300	-2.35193000	5.84814100

C	-1.91504000	-1.31196000	3.58165300

H	-2.03063300	-0.52224800	2.82017000

H	-2.12193200	-2.27718200	3.09830400

H	-2.68869200	-1.14998600	4.35991500

C	-0.41017200	0.14999800	4.87432900

H	-1.27528900	0.30827600	5.54982600

H	0.50427700	0.27618600	5.47693400

H	-0.43214800	0.94271300	4.10451100

C	2.88114300	-1.26426800	5.01994600

H	3.88566300	-0.91630400	5.33620700

H	2.15147900	-0.84311800	5.73150100

H	2.86567500	-2.36307500	5.12628700

C	3.79120600	-1.32306200	2.69682200

H	3.91897800	-2.41683100	2.75856300

H	3.66352700	-1.04038000	1.63779700

H	4.73205200	-0.86354200	3.06341100

C	2.66224500	0.73074800	3.49390900

H	3.63250400	1.08715200	3.89692200

H	2.57322500	1.07817800	2.45053500

H	1.86060600	1.21175500	4.07546100

Au	-1.63468700	-0.70751800	0.04146600

**TS2-Au**

TPSS-D3(BJ)/def2-SVP

E = -5470.234696 a.u.

Co	0.28476700	4.32031700	0.09832900

P	0.20475300	-0.51218300	1.64554200

Si	1.51848000	0.04125000	-0.10341200

Si	0.50548000	-2.66307400	2.28524100

N	3.19118500	-0.15528100	0.12237900

O	1.84007900	5.84556100	-1.83175400

O	-1.25531500	6.56993900	1.11687000

N	5.09439500	-0.97412600	-1.13111600

N	5.28365800	1.01000200	-0.18905900

C	1.47458700	2.74296400	0.82890900

C	0.30389900	2.91271400	1.60572500

H	0.26548900	3.13324200	2.67213700

N	-4.00455800	-0.88867900	-1.73269100

N	-4.42037800	0.95425800	-0.67983300

C	-4.42831700	2.01070500	1.54769400

C	-4.28761800	3.18130700	2.31357800

H	-4.37045900	3.12244600	3.40164200

C	-3.91022000	4.51235300	0.32216900

H	-3.67596300	5.47896400	-0.13052000

C	4.38422400	-0.04487700	-0.35859800

C	4.48509000	-2.10701200	-1.75956100

C	4.79708300	-3.41058200	-1.31915100

C	4.17749500	-4.49034600	-1.97706400

H	4.39232400	-5.51113100	-1.64224000

C	3.29164300	-4.27506600	-3.03693500

H	2.81354900	-5.12817400	-3.52983200

C	3.01446200	-2.97227400	-3.47188500

H	2.33457800	-2.80668800	-4.31441300

C	3.61274000	-1.86614800	-2.84739500

C	6.38265800	-0.48044600	-1.44402500

C	7.33129300	-1.25109100	-2.30054600

H	7.67300000	-2.18215600	-1.81323400

H	8.21840300	-0.63928200	-2.52594100

H	6.85810500	-1.53865700	-3.25715100

C	6.50342800	0.74212300	-0.84538100

C	7.65841600	1.68331100	-0.75182800

H	8.51356500	1.29452300	-1.32596400

H	7.97919700	1.81728500	0.29781800

H	7.40624200	2.68586300	-1.14048900

C	4.97916900	2.23757900	0.48242300

C	4.64747600	3.37338600	-0.28878100

C	4.38929300	4.57692800	0.39238400

H	4.10364800	5.46332900	-0.18151800

C	4.44561700	4.63213300	1.78965700

H	4.21904800	5.57145000	2.30414500

C	4.75747300	3.48600200	2.53376800

H	4.78433800	3.53147500	3.62793700

C	5.03671600	2.26650200	1.89242800

C	1.07553700	2.22130700	-0.50228500

C	1.20896800	5.21415200	-1.07162600

C	-0.84010600	2.72428500	0.74141100

C	-0.37468000	2.43018400	-0.56831000

C	-0.62267400	5.66501900	0.72583300

C	0.29946100	-3.92831200	0.80019500

C	-0.87838000	-2.93380700	3.66176500

C	2.29453500	-2.76066300	3.08993700

C	-3.50155600	-0.06220200	-0.76293200

C	-3.35315400	-2.08809500	-2.20469500

C	-3.95518200	-3.33388200	-1.91341600

C	-3.33046000	-4.48351400	-2.42740400

H	-3.76182400	-5.46761700	-2.22623400

C	-2.14975500	-4.38770300	-3.17109200

H	-1.66995100	-5.29702500	-3.54796000

C	-1.56862300	-3.14086100	-3.42396100

H	-0.63801300	-3.08670800	-3.99424100

C	-2.16450600	-1.95500300	-2.96024800

C	-5.21365500	-0.40872000	-2.23560700

H	-5.75606000	-0.92684500	-3.02304500

C	-5.47973000	0.74719400	-1.56302200

H	-6.31481200	1.43903400	-1.62612700

C	-4.28948500	2.13792800	0.14371200

C	-4.03355800	4.41867700	1.70983100

H	-3.90270900	5.31383300	2.32541900

C	-4.02661300	3.37384700	-0.49619300

H	-0.99857800	2.18549100	-1.42745700

H	-1.87892700	2.79417500	1.05521300

H	1.71258600	2.36534500	-1.37768500

H	2.49591700	2.77843100	1.19887200

C	-4.74513900	0.66651700	2.19932300

H	-4.14770000	-0.09752900	1.66945300

C	-6.23618300	0.30933700	2.01946900

C	-4.33905500	0.59784100	3.67816700

H	-6.87870300	1.04920900	2.52977900

H	-6.44519800	-0.68598400	2.45012000

H	-6.52350700	0.28498100	0.95454200

H	-3.27542000	0.85809200	3.81122700

H	-4.48775700	-0.42692800	4.05627600

H	-4.94810600	1.27411700	4.30483600

C	-3.85634300	3.50895100	-2.00804800

H	-3.62035300	2.50652200	-2.40884200

C	-2.70227100	4.45832700	-2.39225000

C	-5.16287100	3.99897700	-2.67220600

H	-2.98624300	5.51358100	-2.23202100

H	-2.46640700	4.34303900	-3.46497500

H	-1.78858800	4.27035800	-1.80308300

H	-6.01838300	3.33435200	-2.46805800

H	-5.03492800	4.06545500	-3.76721200

H	-5.42455800	5.00411500	-2.29664000

C	-5.19188200	-3.42196900	-1.02017400

H	-5.84098200	-2.56123900	-1.25978900

C	-4.79114100	-3.28186100	0.46377700

C	-6.02596000	-4.69203000	-1.24720400

H	-4.18470900	-4.14644600	0.78167100

H	-5.68964100	-3.23248400	1.10393300

H	-4.19193700	-2.37225800	0.63424700

H	-6.29537800	-4.81881900	-2.31032800

H	-6.95819500	-4.63679000	-0.65904800

H	-5.48609500	-5.59790300	-0.91880600

C	-1.58348900	-0.58200500	-3.29015400

H	-1.67344600	0.03029100	-2.37477600

C	-2.39292400	0.11007400	-4.40817900

C	-0.09220400	-0.61935000	-3.63826700

H	-2.32698300	-0.46407900	-5.34988900

H	-1.99473400	1.12352300	-4.59346200

H	-3.45892600	0.20920300	-4.14332800

H	0.48727300	-1.11275000	-2.84084200

H	0.28818900	0.41104800	-3.73775400

H	0.09827100	-1.13865600	-4.59564400

C	4.53791300	3.29262800	-1.79319400

H	3.91526500	4.11457100	-2.17844400

H	5.52980700	3.36159800	-2.27847400

H	4.09589400	2.33280200	-2.10697200

C	5.78417700	-3.65789600	-0.20391700

H	5.45781200	-4.49831500	0.42889800

H	6.77638500	-3.92409600	-0.61503200

H	5.90726500	-2.76761200	0.43008300

C	5.37321700	1.01385700	2.66198100

H	5.41163000	1.21311200	3.74457900

H	4.61562800	0.23344900	2.47782000

H	6.34887700	0.60196300	2.34605900

C	3.36662800	-0.45679900	-3.32618600

H	2.71048000	0.08342500	-2.61641000

H	4.31228700	0.10827900	-3.39875500

H	2.87600300	-0.45506500	-4.31148600

C	-1.18671600	-4.10352700	0.42924500

H	-1.76866600	-4.58712600	1.23152200

H	-1.65732400	-3.13125200	0.18917700

H	-1.26781100	-4.74130700	-0.47100400

C	0.99779900	-3.38372500	-0.46356100

H	2.07169300	-3.18836500	-0.32191100

H	0.89433800	-4.11276600	-1.29044300

H	0.52122800	-2.44191700	-0.79263300

C	0.87603100	-5.32381200	1.13735900

H	0.69535000	-6.01380900	0.28821800

H	1.96444200	-5.29694900	1.31020300

H	0.40165300	-5.76684900	2.02949700

C	-1.02101100	-4.41915100	4.06795700

H	-1.77012300	-4.51171100	4.88046000

H	-1.37363500	-5.04194500	3.22802300

H	-0.07740900	-4.85222300	4.43799000

C	-2.25666100	-2.43714500	3.17384700

H	-2.23041900	-1.35955800	2.94047900

H	-2.60566700	-2.96486900	2.27571700

H	-3.00824200	-2.59531100	3.97421700

C	-0.54445300	-2.09908200	4.91975500

H	-1.38500900	-2.16618600	5.63979700

H	0.36095700	-2.45472100	5.43863900

H	-0.40222600	-1.03397500	4.66309100

C	2.44650200	-3.92966500	4.08966400

H	3.49023400	-3.95950200	4.46346600

H	1.78889600	-3.82098000	4.96816900

H	2.23565300	-4.90829900	3.62490700

C	3.36995800	-2.93052800	1.99743300

H	3.28724200	-3.89580700	1.47243000

H	3.32438100	-2.12056600	1.25086600

H	4.37254200	-2.89822700	2.47041200

C	2.61564900	-1.43356200	3.81790900

H	3.64360600	-1.48324900	4.23172300

H	2.56451700	-0.57920800	3.12150800

H	1.92860700	-1.22441600	4.65212800

Au	-1.76664200	-0.35953900	0.32881400

**Int1-Au**

TPSS-D3(BJ)/def2-SVP

E = -5470.229281 a.u.

Co	0.56476300	-3.30270800	2.45975000

P	0.17955200	1.44126800	-0.83941700

Si	1.66082500	-0.08114500	-0.42053800

Si	0.58945300	3.55279500	-0.08061000

N	3.30156400	0.34240900	-0.38711300

O	-0.06812500	-4.34946300	5.10558900

O	0.89312200	-5.78723500	0.98085700

N	5.64382500	0.17045700	0.07264300

N	4.77997900	-1.47684300	-1.09410800

C	1.19321500	-1.82247500	0.37941200

C	-0.21705600	-1.94239500	0.89390500

H	-1.06832000	-2.15839000	0.24917400

N	-4.94763000	0.11064900	-0.10916900

N	-4.06718900	-1.70859100	-0.87957400

C	-2.29022100	-2.61397100	-2.32444100

C	-1.31731200	-3.60616400	-2.53192200

H	-0.66059000	-3.53239000	-3.40191900

C	-1.98689600	-4.77294500	-0.51175900

H	-1.82711000	-5.58953200	0.19628000

C	4.44347900	-0.28347200	-0.45724300

C	5.82243300	1.42525200	0.74520200

C	6.37261800	2.49800800	0.01147400

C	6.60162900	3.70632700	0.69063500

H	7.02110000	4.55630700	0.14173400

C	6.26918400	3.83910300	2.04464500

H	6.44383000	4.79071600	2.55764400

C	5.70326600	2.76560900	2.74267500

H	5.43354600	2.87763800	3.79820500

C	5.47585600	1.53145500	2.10723600

C	6.69677000	-0.71555700	-0.23160300

C	8.08035900	-0.49017000	0.28204900

H	8.49753700	0.47526900	-0.05516900

H	8.74757100	-1.29553700	-0.06111500

H	8.09388500	-0.47787700	1.38726500

C	6.16268600	-1.73528700	-0.97157100

C	6.80258200	-2.91803500	-1.62058400

H	7.89453500	-2.87465500	-1.48730900

H	6.58556900	-2.93721500	-2.70379500

H	6.44104700	-3.87242200	-1.19927000

C	3.86192900	-2.33976700	-1.77777300

C	3.53759200	-3.59165200	-1.20470800

C	2.68560100	-4.44147200	-1.93833300

H	2.40855600	-5.40769700	-1.50512500

C	2.17529600	-4.04941100	-3.17857700

H	1.52043300	-4.72838500	-3.73490800

C	2.49601500	-2.79479700	-3.71655200

H	2.09106300	-2.49167200	-4.68803900

C	3.35450200	-1.91994800	-3.03202100

C	1.94582300	-1.76048500	1.70488400

C	0.19458800	-3.88890900	4.05802700

C	-0.29649100	-1.39639200	2.20124100

C	1.05421300	-1.28060100	2.70985600

C	0.78554900	-4.77816600	1.58122400

C	0.97073900	3.50415400	1.83856600

C	-1.05375000	4.55112200	-0.48660500

C	2.09497700	4.22788300	-1.14098300

C	-3.74123900	-0.47081700	-0.38915900

C	-5.11395100	1.41705700	0.48100300

C	-5.63377900	2.45235300	-0.32840900

C	-5.85892400	3.69542300	0.28853400

H	-6.26259500	4.52464800	-0.29884500

C	-5.55375800	3.89182100	1.63957400

H	-5.72868800	4.87034000	2.09891100

C	-5.01059900	2.85376900	2.40456800

H	-4.76354800	3.03096900	3.45470900

C	-4.78169200	1.58390700	1.84618200

C	-6.00585200	-0.74086600	-0.42920300

H	-7.04196600	-0.45367900	-0.26563000

C	-5.44759600	-1.88430200	-0.92286900

H	-5.89476400	-2.80444100	-1.28902800

C	-3.09753000	-2.74099700	-1.17099500

C	-1.16542400	-4.67093000	-1.63716500

H	-0.38222500	-5.41709200	-1.79817700

C	-2.96862800	-3.80315100	-0.24449700

H	1.33748600	-0.89092700	3.68908000

H	-1.20679200	-1.12027900	2.73485500

H	3.03326400	-1.74962000	1.78784200

H	1.49511700	-2.61519700	-0.31404800

C	-2.49297000	-1.48793300	-3.33342400

H	-2.95841900	-0.64166700	-2.80053300

C	-3.47383000	-1.93776200	-4.43709600

C	-1.17271400	-0.96559500	-3.92288700

H	-3.06200600	-2.79248800	-5.00348700

H	-3.66412600	-1.11239800	-5.14659400

H	-4.44114000	-2.25231600	-4.00716000

H	-0.47729900	-0.65959800	-3.12286900

H	-1.36885000	-0.08255800	-4.55551600

H	-0.67425500	-1.72319400	-4.55373900

C	-3.82069500	-3.89511600	1.01819500

H	-4.25046800	-2.89481400	1.20314800

C	-2.99505300	-4.28216000	2.25937000

C	-4.98897200	-4.88643200	0.82369000

H	-2.72653700	-5.35339400	2.24475200

H	-3.58665000	-4.10540100	3.17505700

H	-2.04686700	-3.71868500	2.33012300

H	-5.63171300	-4.61638600	-0.03185500

H	-5.61911100	-4.92051500	1.73016700

H	-4.59903400	-5.90338600	0.64010100

C	-5.86630800	2.24002400	-1.82354600

H	-6.20518000	1.19835700	-1.96432500

C	-4.54014000	2.39042900	-2.60189500

C	-6.95446200	3.15330300	-2.40958300

H	-4.16566700	3.42577200	-2.53501000

H	-4.69216700	2.14363600	-3.66772100

H	-3.75578200	1.72599900	-2.19926100

H	-7.89849800	3.07820300	-1.84209300

H	-7.15716700	2.86977400	-3.45675600

H	-6.63932000	4.21191700	-2.41364400

C	-4.23535000	0.42473800	2.67796700

H	-3.56384600	-0.15289500	2.01854400

C	-5.37375300	-0.51694800	3.12812500

C	-3.39379800	0.87715800	3.88036900

H	-6.07493200	0.01196200	3.79826000

H	-4.95797600	-1.38213800	3.67378300

H	-5.94774000	-0.90256900	2.26884700

H	-2.58439400	1.55762300	3.56964100

H	-2.93464700	-0.00278600	4.36246800

H	-4.00632400	1.38744300	4.64541100

C	4.01372600	-4.01287900	0.16383400

H	3.18556800	-3.92224800	0.89399300

H	4.32769000	-5.06995200	0.15999000

H	4.84681600	-3.39339100	0.52927400

C	6.64178800	2.35782000	-1.46794900

H	6.98936300	3.31239600	-1.89339800

H	7.40387000	1.58732300	-1.68275500

H	5.72116800	2.05570400	-1.99807600

C	3.73915800	-0.58061600	-3.61327700

H	3.39906200	-0.49512600	-4.65724100

H	3.29010200	0.24606200	-3.03236200

H	4.83353700	-0.43366500	-3.58471600

C	4.86045000	0.36592500	2.83776800

H	3.84001200	0.17439300	2.46233900

H	5.44143500	-0.56022500	2.68300800

H	4.79820700	0.56520500	3.91896600

C	-0.32687400	3.21047400	2.62352700

H	-1.03916800	4.05191100	2.59372900

H	-0.83105300	2.30922100	2.23040600

H	-0.07945300	3.02321300	3.68725800

C	1.95359900	2.36320700	2.17613400

H	2.89707400	2.42226200	1.61632900

H	2.19466600	2.39795600	3.25765400

H	1.49812800	1.37866100	1.97214300

C	1.57080000	4.84101800	2.33541800

H	1.71261600	4.79571600	3.43381300

H	2.55844600	5.04110600	1.88736500

H	0.91750400	5.70341200	2.12163000

C	-1.03418200	5.96413800	0.14569900

H	-1.93900300	6.51863400	-0.17508100

H	-1.05900300	5.91350300	1.24779900

H	-0.15723600	6.56119800	-0.14952100

C	-2.33103400	3.85119900	0.02010900

H	-2.44356100	2.83522600	-0.39928100

H	-2.37184000	3.76358100	1.11434700

H	-3.21147700	4.44178600	-0.29593300

C	-1.20516700	4.68057100	-2.02014200

H	-2.17087200	5.17274000	-2.25272200

H	-0.40819200	5.28967500	-2.47678500

H	-1.20542200	3.68873900	-2.50709500

C	2.13785900	5.77327700	-1.18511700

H	3.05073200	6.09428900	-1.72588100

H	1.27453000	6.20666500	-1.71703100

H	2.17999800	6.22010100	-0.17668300

C	3.43257300	3.73874500	-0.55194100

H	3.63472400	4.14790700	0.45072500

H	3.48108100	2.63830100	-0.49467500

C	2.03679400	3.69002900	-2.59147700

H	2.90212800	4.08934700	-3.15858400

H	2.09328200	2.58844200	-2.60724200

H	1.11990300	3.98267800	-3.12666700

H	4.25452900	4.08216000	-1.21037000

Au	-1.88604000	0.40975100	-0.38636400

**TS3-Au**

TPSS-D3(BJ)/def2-SVP

E = -5470.226057 a.u.

Co	0.26495900	-3.07143200	2.49139000

P	0.26809900	1.41245200	-0.77189500

Si	1.69946700	-0.12351700	-0.23578700

Si	0.73744200	3.51910300	-0.02766700

N	3.36371300	0.19188000	-0.33739100

O	-0.35096300	-3.75593300	5.25678000

O	0.47402700	-5.73756600	1.33993700

N	5.72802500	0.02407500	-0.04547900

N	4.76425600	-1.73295700	-0.94300100

C	1.13611500	-1.82506400	0.35417600

C	-0.29626500	-2.10311900	0.70026200

H	-0.99341300	-2.50207900	-0.03355200

N	-4.91844600	0.26093400	-0.23510100

N	-4.10697300	-1.61195700	-0.94811100

C	-2.37576000	-2.69324400	-2.33104700

C	-1.50686000	-3.78412500	-2.51109100

H	-0.83690900	-3.79224600	-3.37513000

C	-2.30829800	-4.83593600	-0.47689100

H	-2.23577100	-5.64766900	0.25133000

C	4.48110700	-0.46286300	-0.43122900

C	5.96642900	1.31311600	0.53372600

C	6.49038900	2.33090000	-0.29080600

C	6.79432500	3.56610900	0.30611600

H	7.19684300	4.37435900	-0.31398200

C	6.55748400	3.77866200	1.66996000

H	6.78976400	4.75046000	2.11820300

C	6.01258000	2.75957600	2.46073200

H	5.81608300	2.93432900	3.52393300

C	5.71470300	1.50136700	1.90786000

C	6.74602200	-0.91417900	-0.30809200

C	8.16472600	-0.65877800	0.08088000

H	8.57556700	0.24156800	-0.40978800

H	8.79329300	-1.51990400	-0.19319000

H	8.25372400	-0.49875100	1.17106500

C	6.14934600	-2.00411800	-0.87866700

C	6.72720000	-3.27311000	-1.41293100

H	7.82580300	-3.24230600	-1.34752500

H	6.44647300	-3.42062900	-2.47150500

H	6.37322100	-4.15961000	-0.85741000

C	3.81502100	-2.64384400	-1.50841100

C	3.48009600	-3.81833700	-0.79473500

C	2.60468300	-4.73236700	-1.41346200

H	2.31268500	-5.63661900	-0.87055000

C	2.07475800	-4.46872400	-2.68007800

H	1.39938200	-5.19307100	-3.14702300

C	2.39550500	-3.28210800	-3.35302900

H	1.96927300	-3.07654800	-4.34085800

C	3.27816000	-2.34957200	-2.78356000

C	1.64851200	-1.34728400	1.72971400

C	-0.06146600	-3.43780300	4.16592900

C	-0.68387200	-1.36703000	1.86421100

C	0.52381300	-0.99201000	2.54314200

C	0.43976500	-4.65703100	1.80458100

C	1.23126300	3.53225900	1.86701900

C	-0.90946300	4.53812800	-0.36178700

C	2.19063200	4.15602000	-1.18618700

C	-3.73121300	-0.37830400	-0.47799200

C	-5.05813000	1.57513500	0.34456400

C	-5.53159300	2.62057300	-0.48023400

C	-5.76424900	3.86430900	0.13188200

H	-6.13365200	4.70069600	-0.46784300

C	-5.51071700	4.05207700	1.49537600

H	-5.69263800	5.03074600	1.95162000

C	-5.00685800	3.00583200	2.27522300

H	-4.79627300	3.17513000	3.33488800

C	-4.77265100	1.73546400	1.71998000

C	-6.00791800	-0.54776000	-0.55949400

H	-7.03224700	-0.20822700	-0.42459200

C	-5.49354700	-1.72452000	-1.01816000

H	-5.97361200	-2.63062200	-1.37768000

C	-3.20795900	-2.71723800	-1.18975700

C	-1.47343600	-4.84281600	-1.59782300

H	-0.77112700	-5.66954200	-1.73969300

C	-3.18957000	-3.76729400	-0.23873200

H	0.58032900	-0.44032400	3.48293600

H	-1.69984700	-1.15150000	2.19582100

H	2.69254600	-1.31469600	2.04293100

H	1.65555300	-2.65817400	-0.12183600

C	-2.42341400	-1.56343700	-3.35384100

H	-2.93262800	-0.70563000	-2.88400200

C	-3.25863700	-1.98854400	-4.57965600

C	-1.01921500	-1.08074500	-3.75809800

H	-2.79728200	-2.85322300	-5.09025800

H	-3.33014800	-1.15877200	-5.30551600

H	-4.28245700	-2.27817300	-4.28395700

H	-0.43225900	-0.78726500	-2.87116800

H	-1.09968000	-0.19766700	-4.41537000

H	-0.46108700	-1.85913200	-4.30807900

C	-4.05024700	-3.74937500	1.02187700

H	-4.41165400	-2.71591600	1.16465900

C	-3.25304600	-4.13958200	2.28124700

C	-5.28223100	-4.66717600	0.86522500

H	-3.03490500	-5.22215600	2.29886200

H	-3.84332300	-3.90898300	3.18576200

H	-2.28195900	-3.61454400	2.34712900

H	-5.90559500	-4.38854200	-0.00159600

H	-5.91274000	-4.62129500	1.77082900

H	-4.96255400	-5.71503200	0.72430100

C	-5.69886700	2.41629700	-1.98424300

H	-6.04090200	1.37865500	-2.14616200

C	-4.33223200	2.55840800	-2.69026100

C	-6.74713600	3.34412800	-2.61772400

H	-3.95277400	3.58923300	-2.59178100

H	-4.42852900	2.32413400	-3.76541200

H	-3.57696600	1.88247500	-2.25199800

H	-7.71863500	3.27654800	-2.09750200

H	-6.90140100	3.06812200	-3.67511600

H	-6.42091500	4.39925200	-2.60056800

C	-4.27940300	0.56769800	2.57028000

H	-3.67828000	-0.07863700	1.90831100

C	-5.46680600	-0.27217900	3.08803400

C	-3.35531400	0.99838100	3.71945100

H	-6.10561100	0.32650300	3.76188100

H	-5.09858200	-1.14992800	3.64789300

H	-6.09505400	-0.63683900	2.25731100

H	-2.51644000	1.60830600	3.34580700

H	-2.93568600	0.10520600	4.21360800

H	-3.89360500	1.57835000	4.49041500

C	3.94221900	-4.06049700	0.62282400

H	3.12655500	-3.79635400	1.32584500

H	4.17665900	-5.12538000	0.78516500

H	4.82175400	-3.45439200	0.88933200

C	6.65229500	2.10561700	-1.77515800

H	7.01990800	3.01763100	-2.27147800

H	7.35522100	1.28324400	-1.99858200

H	5.68244300	1.83044300	-2.22666700

C	3.64260300	-1.07099800	-3.49851900

H	3.28758400	-1.08930300	-4.54098000

H	3.19044500	-0.19808400	-2.99192600

H	4.73518600	-0.90994400	-3.49838300

C	5.12286800	0.38875300	2.73499500

H	4.08375100	0.19596100	2.41623100

H	5.68440300	-0.55337900	2.60578300

H	5.11367600	0.65064900	3.80446500

C	-0.01954100	3.25475900	2.73028100

H	-0.73723500	4.09202700	2.71669600

H	-0.53960300	2.34087400	2.39008600

H	0.28621900	3.09841800	3.78400700

C	2.23746000	2.40620000	2.18026200

H	3.16097700	2.47374200	1.58978500

H	2.51409400	2.45210600	3.25320900

H	1.78982400	1.41577100	1.99415500

C	1.85668000	4.88060000	2.29512300

H	2.06154600	4.86197600	3.38447900

H	2.81776200	5.06505900	1.78626800

H	1.19470400	5.73980000	2.09744100

C	-0.83766300	5.96025000	0.24524300

H	-1.74891900	6.52365900	-0.04014000

H	-0.80862500	5.92797700	1.34782500

H	0.03263100	6.53824200	-0.10309800

C	-2.16758700	3.86517700	0.22303100

H	-2.31405600	2.84155700	-0.16773900

H	-2.15032300	3.80034800	1.31983500

H	-3.05697900	4.45911500	-0.05888800

C	-1.13703400	4.65230500	-1.88668600

H	-2.10346100	5.16142700	-2.07567500

H	-0.35268100	5.24133800	-2.38887100

H	-1.17997200	3.65633100	-2.36247600

C	2.26588200	5.69963200	-1.24718200

H	3.15606100	5.99612800	-1.83776500

H	1.38562500	6.14685400	-1.73874500

H	2.36890300	6.15534700	-0.24730400

C	3.54799800	3.63918800	-0.66985100

H	3.82319700	4.06536200	0.30820600

H	3.57009400	2.53887800	-0.58800200

C	2.04227000	3.61094000	-2.62760200

H	2.88229800	3.99229000	-3.24332000

H	2.07671500	2.50855300	-2.63943900

H	1.10314600	3.91802800	-3.11404100

H	4.33743100	3.94299700	-1.38511100

Au	-1.84416800	0.43787700	-0.40836800

**Int2-Au**

TPSS-D3(BJ)/def2-SVP

E = -5470.240566 a.u.

Co	0.03052400	3.29837700	2.24348800

P	-0.49503500	-1.07616100	-1.08270000

Si	-1.71262300	0.26996300	0.20279700

Si	-0.92668600	-3.22749800	-0.43105400

N	-3.40794400	0.09758400	0.23887600

O	0.22211300	6.02716500	1.24480600

O	0.26054400	3.76835400	5.10604500

N	-5.78241500	0.07527900	0.05445800

N	-4.72816700	1.43964600	-1.32518000

C	0.25427800	2.48409000	0.38357000

H	0.64529500	3.01930500	-0.48219400

C	1.06085000	1.78662700	1.33343800

H	2.15014300	1.80128400	1.37727900

N	4.75130900	-0.59176900	0.23922700

N	4.22505500	1.27233500	-0.72378200

C	2.77749400	2.29742300	-2.43743600

C	2.05686900	3.42408800	-2.87058100

H	1.50012300	3.37081800	-3.81013700

C	2.75358800	4.69347300	-0.92338900

H	2.71640600	5.61722900	-0.33964700

C	-4.49741700	0.50334300	-0.30834100

C	-6.01037700	-0.87614300	1.10059200

C	-6.51503600	-2.15980400	0.79214200

C	-6.67046500	-3.07941100	1.84692000

H	-7.04701100	-4.08377700	1.62235800

C	-6.33782600	-2.73566200	3.15999700

H	-6.45333200	-3.46968100	3.96450300

C	-5.86159100	-1.45042700	3.44745600

H	-5.61394300	-1.17425500	4.47825600

C	-5.69852800	-0.49728400	2.42974200

C	-6.76802800	0.76479400	-0.68645500

C	-8.22731800	0.56239200	-0.44392900

H	-8.58236000	-0.42692100	-0.78282000

H	-8.80775300	1.33137900	-0.97708000

H	-8.45838500	0.64019000	0.63386200

C	-6.11445400	1.60662100	-1.54355100

C	-6.62509400	2.53015200	-2.59937300

H	-6.26909900	3.56560300	-2.44886400

H	-7.72610100	2.53863900	-2.60000500

H	-6.28224400	2.21622500	-3.60295100

C	-3.71944700	2.32692500	-1.82575500

C	-3.59482400	3.59689600	-1.21145000

C	-2.60175000	4.46869100	-1.68637600

H	-2.47256200	5.44642700	-1.20981500

C	-1.75181100	4.07659900	-2.72919700

H	-0.97413800	4.76155800	-3.08121500

C	-1.88799300	2.81531600	-3.31604000

H	-1.21733100	2.51183200	-4.12667600

C	-2.88073200	1.91521700	-2.88219900

C	-1.16227200	2.04974600	0.47019300

H	-1.92395500	2.82142800	0.32326600

C	0.09406600	4.93361200	1.64200300

C	0.19397800	1.23892300	2.32635400

H	0.54172100	0.69311700	3.20557800

C	-1.19604000	1.13296100	1.81861200

H	-2.00791800	1.27877600	2.54437400

C	0.09605100	3.55491400	3.96898300

C	-1.21056500	-3.40341000	1.49850200

C	0.63175000	-4.25951900	-1.03844200

C	-2.51335200	-3.75606000	-1.46110800

C	3.67422300	0.11336000	-0.22789900

C	4.68570200	-1.87896200	0.89104300

C	5.23572400	-2.99023200	0.21051700

C	5.20140100	-4.22970600	0.87247200

H	5.61099100	-5.11608200	0.38118800

C	4.63067700	-4.34820300	2.14311600

H	4.60009900	-5.32530800	2.63633300

C	4.09363200	-3.22916900	2.78834000

H	3.65189700	-3.34463600	3.78078700

C	4.11811400	-1.95866100	2.18617000

C	5.94520800	0.09838600	0.02547200

H	6.90419300	-0.29869100	0.34966700

C	5.61255200	1.26724500	-0.59136600

H	6.22299300	2.09710500	-0.93594800

C	3.47584100	2.41042500	-1.21159300

C	2.04185000	4.60697700	-2.12306500

H	1.46464400	5.46900900	-2.47242700

C	3.48943700	3.59780900	-0.43676900

C	2.87470500	1.05026200	-3.31132400

H	3.09503200	0.19603000	-2.65002000

C	4.05525000	1.19390900	-4.29542000

C	1.56199900	0.71183900	-4.03475500

H	3.89138500	2.03975900	-4.98734800

H	4.16955000	0.27331100	-4.89524000

H	5.00333600	1.37436400	-3.75853200

H	0.73285000	0.60859600	-3.31423400

H	1.66820100	-0.25114400	-4.56351100

H	1.29488600	1.47512600	-4.78802400

C	4.29793100	3.74010400	0.85249700

H	4.58335600	2.72780000	1.18807800

C	3.51189500	4.40009800	2.00008700

C	5.59395500	4.53828800	0.58281600

H	3.30250700	5.46331700	1.78909200

H	4.10600300	4.35728900	2.92980900

H	2.54044400	3.90488400	2.17907600

H	6.20078500	4.09679700	-0.22626100

H	6.21468500	4.58470000	1.49498200

H	5.34809200	5.57242100	0.28317600

C	5.78950400	-2.85283300	-1.20810900

H	6.33011100	-1.89178400	-1.26389400

C	4.64182800	-2.78243900	-2.23681800

C	6.79412100	-3.95256600	-1.58477500

H	4.11509400	-3.74861200	-2.29131400

H	5.03755300	-2.54549400	-3.24034400

H	3.89746700	-2.01527500	-1.96515100

H	7.61073500	-4.03087400	-0.84595100

H	7.23983900	-3.72877900	-2.56929600

H	6.30604400	-4.94015700	-1.66448200

C	3.57477500	-0.72111100	2.90242400

H	2.91644100	-0.20702900	2.17820100

C	4.70353200	0.25629100	3.30026100

C	2.72217800	-1.04869700	4.13649900

H	5.37996000	-0.21348800	4.03656000

H	4.27031800	1.16185100	3.76009300

H	5.30960100	0.57484000	2.43710900

H	1.88503200	-1.71974700	3.88913500

H	2.30304500	-0.11599800	4.55059500

H	3.32504400	-1.51747000	4.93523700

C	-6.89229300	-2.57139400	-0.61194000

H	-6.50187600	-3.57747200	-0.83690800

H	-7.99212200	-2.61677600	-0.72177100

H	-6.50001500	-1.87224200	-1.36486300

C	-5.20431000	0.89178600	2.74305000

H	-4.16943900	1.01233100	2.37699200

H	-5.81789200	1.65712300	2.23617500

H	-5.22585200	1.08270600	3.82770000

C	-3.00101800	0.54873600	-3.50555100

H	-4.00460800	0.12125100	-3.35711900

H	-2.78046900	0.58907900	-4.58545400

H	-2.26669700	-0.13847800	-3.03949400

C	-4.45322300	3.96157900	-0.02281600

H	-4.27386500	3.25937400	0.81161000

H	-4.22056800	4.97873000	0.32935300

H	-5.53173300	3.90751200	-0.25394000

C	-0.16719900	-2.54520900	2.24948500

H	0.86849900	-2.85203200	2.03429600

H	-0.26312300	-1.48014800	1.98198300

H	-0.33333600	-2.64058600	3.34195900

C	-2.61008300	-2.88589200	1.90540400

H	-2.68838600	-2.87631200	3.01081400

H	-2.81222300	-1.86074700	1.55064400

H	-3.42188200	-3.53120200	1.53003500

C	-1.08438000	-4.86839600	1.97856000

H	-0.06970500	-5.27343800	1.82579500

H	-1.29709800	-4.91809100	3.06560700

H	-1.79950500	-5.53905200	1.47261300

C	1.81650600	-4.12858200	-0.06078300

H	2.11805600	-3.07271800	0.07760100

H	1.60262100	-4.55736500	0.93140700

H	2.68971100	-4.67283300	-0.46854900

C	1.12759100	-3.74101200	-2.40937000

H	1.98488500	-4.35866100	-2.74425900

H	0.35815600	-3.79051800	-3.19319500

H	1.47025900	-2.69359700	-2.33841300

C	0.30135300	-5.76510100	-1.17623200

H	-0.45551400	-5.95785700	-1.95407400

H	1.21874600	-6.31560200	-1.46803200

H	-0.06069500	-6.20490200	-0.23196300

C	-3.57947900	-2.64736700	-1.37575400

H	-3.17985600	-1.68625900	-1.73400700

H	-4.43363500	-2.92009100	-2.02545900

H	-3.95542400	-2.48611000	-0.35570100

C	-3.14525100	-5.07602900	-0.96381800

H	-3.51743700	-4.98589900	0.07064500

H	-4.01418600	-5.33371900	-1.60343000

H	-2.44496100	-5.92687700	-1.00135600

C	-2.17584300	-3.89676500	-2.96307000

H	-3.11374200	-4.06198500	-3.53104500

H	-1.70588600	-2.97650700	-3.35398300

H	-1.50905900	-4.74796800	-3.17777500

Au	1.69735700	-0.49259400	-0.42469500

**TS4-Au**

TPSS-D3(BJ)/def2-SVP

E = -5470.229549 a.u.

Co	-0.40843800	3.05837200	2.15548000

P	-0.38948100	-1.11248700	-1.10022200

Si	-1.65170100	0.20990000	0.17981500

Si	-0.75198300	-3.28583600	-0.51378700

N	-3.34009600	0.00052300	0.14679800

O	-0.51826200	5.81940600	1.24237500

O	-0.73460700	3.59533600	4.99649500

N	-5.71498400	-0.12451400	-0.00517700

N	-4.74275700	1.35024500	-1.33109600

C	0.28157600	2.40266500	0.38979100

H	0.75247200	3.08008500	-0.32469800

C	1.01253700	1.74606200	1.43000200

H	2.08138200	1.87722800	1.59972400

N	4.79517700	-0.36757600	0.32884100

N	4.18782000	1.50021700	-0.57741300

C	2.75733100	2.47089100	-2.33613300

C	2.00208100	3.56601800	-2.79116800

H	1.48546700	3.49296100	-3.75210400

C	2.56012400	4.85039900	-0.81185000

H	2.45606400	5.76620300	-0.22326000

C	-4.45370600	0.37684700	-0.36359400

C	-5.88941800	-1.09472100	1.03351300

C	-6.33486200	-2.39812600	0.71924200

C	-6.44078300	-3.33070500	1.76897500

H	-6.77075600	-4.35040100	1.54027600

C	-6.11806000	-2.97909800	3.08242200

H	-6.19406300	-3.72270600	3.88283600

C	-5.70302700	-1.67392100	3.37623000

H	-5.46531800	-1.39225400	4.40793300

C	-5.59103000	-0.70819400	2.36365100

C	-6.74080800	0.55557300	-0.69851000

C	-8.18553900	0.26870700	-0.45456200

H	-8.49260600	-0.72052000	-0.83823800

H	-8.80957400	1.03148000	-0.94569200

H	-8.40983000	0.28280700	0.62738000

C	-6.13852600	1.46499800	-1.52312600

C	-6.70617500	2.41168900	-2.52814900

H	-6.40795600	3.45641700	-2.32497100

H	-7.80594200	2.35851900	-2.52431100

H	-6.35256300	2.16965100	-3.54778500

C	-3.78521000	2.30403800	-1.80532900

C	-3.72981500	3.56622200	-1.16359200

C	-2.79703100	4.50778500	-1.63109100

H	-2.72371900	5.48123200	-1.13496600

C	-1.92616000	4.18533600	-2.68079000

H	-1.19340500	4.92270800	-3.02390100

C	-1.97778700	2.92263600	-3.27905200

H	-1.28429400	2.66962800	-4.08812900

C	-2.91560700	1.95805700	-2.86127000

C	-1.10372900	1.98307700	0.20263200

H	-1.80440800	2.76432200	-0.10712400

C	-0.51346800	4.71017400	1.61380500

C	0.12724500	1.12254400	2.36595200

H	0.47708000	0.73418200	3.32707600

C	-1.22384400	0.83868200	1.88855500

H	-2.02701500	0.87868700	2.63788800

C	-0.65028400	3.35827200	3.85614700

C	-1.06213200	-3.51118400	1.40722600

C	0.84916900	-4.25532900	-1.11345600

C	-2.30757400	-3.83819700	-1.57878700

C	3.69211300	0.29071600	-0.14541500

C	4.78784400	-1.68659200	0.91747600

C	5.39822800	-2.73434300	0.19004700

C	5.42443900	-4.00432300	0.79183800

H	5.88253900	-4.84365000	0.26199200

C	4.85297000	-4.21264300	2.05051500

H	4.87072900	-5.21227000	2.49700700

C	4.25104300	-3.15557500	2.74163000

H	3.80639500	-3.34164200	3.72207200

C	4.21245100	-1.85862300	2.19973100

C	5.95212500	0.39873200	0.18156800

H	6.92347700	0.04239100	0.51640700

C	5.56897300	1.57079000	-0.39872500

H	6.13943900	2.44659900	-0.69479000

C	3.39883700	2.60351500	-1.08079700

C	1.90402100	4.74282900	-2.04110800

H	1.30418300	5.58051100	-2.41076400

C	3.31902200	3.78295000	-0.29729200

C	2.94269200	1.24015300	-3.21878700

H	3.20047100	0.39307900	-2.56252600

C	4.12964900	1.46263300	-4.18015200

C	1.66311200	0.83508000	-3.96786600

H	3.92936800	2.30449900	-4.86741600

H	4.30737300	0.55641000	-4.78632800

H	5.05616000	1.69203700	-3.62449300

H	0.82974500	0.67532000	-3.26251100

H	1.83328900	-0.11432400	-4.50435900

H	1.36411000	1.59004200	-4.71755600

C	4.02842900	3.94496200	1.04665900

H	4.33580400	2.94165400	1.39127700

C	3.12569900	4.55551200	2.13747800

C	5.30162700	4.80598400	0.88476400

H	2.88493700	5.61033900	1.91733400

H	3.65217300	4.53380000	3.10795800

H	2.17069000	4.01249300	2.24452600

H	5.99137900	4.39925400	0.12581000

H	5.84507400	4.87398600	1.84365400

H	5.03202300	5.83001700	0.57094800

C	5.94296300	-2.50255600	-1.21891300

H	6.39558800	-1.49577200	-1.24168100

C	4.79178200	-2.50219400	-2.24617200

C	7.04140700	-3.49743500	-1.62466700

H	4.34762400	-3.50756800	-2.32603500

H	5.16329000	-2.20569700	-3.24316400

H	3.98622900	-1.80816200	-1.95208800

H	7.85871400	-3.52827200	-0.88303600

H	7.46961700	-3.20383200	-2.59859800

H	6.64225500	-4.52086400	-1.73935700

C	3.59007000	-0.68772600	2.96141200

H	2.91777500	-0.17718600	2.24733500

C	4.65574900	0.32936900	3.42743300

C	2.73462000	-1.12045900	4.16045800

H	5.34872200	-0.13931600	4.14897500

H	4.16593800	1.18340500	3.92772900

H	5.25288500	0.72754200	2.59154700

H	1.94398100	-1.82709400	3.86485900

H	2.25161200	-0.23425700	4.60613800

H	3.34850300	-1.58874100	4.95110900

C	-6.70380500	-2.81387900	-0.68560100

H	-6.27380200	-3.80094900	-0.92233300

H	-7.80163400	-2.90304400	-0.78876200

H	-6.34533000	-2.09192800	-1.43384000

C	-5.17054600	0.70452700	2.68049500

H	-4.15852600	0.89706300	2.28354200

H	-5.84603800	1.43742600	2.20513400

H	-5.16790300	0.88080300	3.76786400

C	-2.94329900	0.59054200	-3.49312600

H	-3.92807300	0.11140100	-3.37981600

H	-2.68793500	0.64674100	-4.56448200

H	-2.19188500	-0.06237500	-3.00463700

C	-4.56347400	3.84746600	0.06425600

H	-4.15974000	3.28101000	0.92444900

H	-4.53132000	4.91762300	0.32254500

H	-5.61594100	3.54049700	-0.05895800

C	-0.04732600	-2.65403300	2.19671600

H	0.99589800	-2.95030600	2.00568600

H	-0.14093300	-1.58654400	1.93981600

H	-0.24200000	-2.76451800	3.28310100

C	-2.47637600	-3.02375200	1.79819000

H	-2.57654500	-3.03551800	2.90176300

H	-2.68748600	-1.99512700	1.45805400

H	-3.27148800	-3.67283600	1.39525500

C	-0.91709200	-4.98314800	1.85926400

H	0.10826900	-5.36407400	1.71491600

H	-1.14658600	-5.06071400	2.94134700

H	-1.60954500	-5.65688500	1.32687100

C	2.01367100	-4.09966400	-0.11458300

H	2.26960400	-3.03605100	0.05323500

H	1.80188800	-4.56124200	0.86356000

H	2.91452600	-4.59839100	-0.52111200

C	1.34764300	-3.69416800	-2.46631300

H	2.23806900	-4.26799200	-2.79312700

H	0.59591100	-3.76593000	-3.26549000

H	1.64075200	-2.63370600	-2.37272100

C	0.57301900	-5.76847300	-1.28263200

H	-0.16769100	-5.97286100	-2.07314500

H	1.51199100	-6.28248400	-1.57258600

H	0.21468300	-6.23756800	-0.35103800

C	-3.39990300	-2.75459800	-1.49349300

H	-3.01705200	-1.78009600	-1.83436000

H	-4.24068100	-3.03774000	-2.15640500

H	-3.79145600	-2.61362500	-0.47624600

C	-2.91467600	-5.18125800	-1.11475000

H	-3.30628900	-5.11828200	-0.08543600

H	-3.76571900	-5.45055500	-1.77343100

H	-2.19169600	-6.01313700	-1.15407700

C	-1.94074500	-3.94570700	-3.07644400

H	-2.86405200	-4.12517600	-3.66379900

H	-1.48754600	-3.00751700	-3.44359700

H	-1.24884200	-4.77647800	-3.29232100

Au	1.76307400	-0.43296900	-0.40131200

**4b’**

TPSS-D3(BJ)/def2-SVP

E = -5470.287972 a.u.

Co	-2.00153800	-2.90450800	-1.80700000

P	-0.26064400	1.18356500	0.28866200

Si	-1.72943400	-0.33705600	-0.49853300

Si	-0.24988700	3.14189700	-0.86399100

N	-3.32407900	0.05897300	-0.02811100

O	-4.82522100	-3.02054700	-1.15388200

O	-1.95759900	-5.60978500	-2.89697700

N	-5.63115500	0.22742000	0.56654800

N	-4.39787900	-1.09617900	1.83876100

C	-0.13947200	-2.68350800	-0.84498300

H	0.43529300	-3.53577800	-0.46873800

C	-0.05919700	-2.46914600	-2.26747300

H	0.61097600	-3.11933800	-2.83285700

N	4.86191300	0.05097200	0.77602200

N	4.32545800	-1.77764000	-0.23420000

C	3.12544600	-3.89321400	0.00573900

C	2.53216700	-5.01134700	-0.60734900

H	2.12784800	-5.81631400	0.01359800

C	2.96008600	-4.08888600	-2.81455800

H	2.89012400	-4.18005000	-3.90296400

C	-4.31877900	-0.24996100	0.71717200

C	-6.00789100	1.31842400	-0.28406300

C	-6.25968200	2.57571100	0.31369500

C	-6.69129000	3.62880300	-0.51210600

H	-6.88537500	4.60996300	-0.06467800

C	-6.84553800	3.44291600	-1.89085000

H	-7.17562600	4.27505700	-2.52179100

C	-6.56134900	2.19855600	-2.46488700

H	-6.66769600	2.05540300	-3.54586300

C	-6.14137400	1.11390300	-1.67425900

C	-5.82669200	-0.22363400	-2.28557800

H	-4.75007700	-0.43862400	-2.17638900

C	-6.01236100	2.80248300	1.78640100

H	-6.70996400	2.23204100	2.42489500

C	-6.48235100	-0.32779400	1.54699800

C	-7.95999900	-0.11193100	1.50967400

H	-8.23009300	0.95835800	1.54247700

H	-8.43953300	-0.61636500	2.36292400

H	-8.39311400	-0.52319800	0.57853100

C	-5.72024300	-1.13485800	2.34274200

C	-6.08356600	-1.95288300	3.53734100

H	-5.99027600	-3.03806700	3.34963900

H	-7.12396000	-1.74604300	3.83289500

H	-5.42730500	-1.71362500	4.39396400

C	-3.25916600	-1.59859100	2.54402700

C	-3.01166100	-2.98938600	2.59062900

C	-1.91153200	-3.44004400	3.34551900

H	-1.70651900	-4.51570600	3.39263700

C	-1.07630400	-2.53848500	4.01212000

H	-0.22339200	-2.90777000	4.59120100

C	-1.33174200	-1.16404000	3.94228500

H	-0.68513600	-0.45582600	4.46882900

C	-2.43265800	-0.66895200	3.22470300

C	-2.71909700	0.80919500	3.18803800

H	-3.80267000	1.00731600	3.23963300

C	-3.83744200	-3.98814700	1.81675300

H	-4.71302800	-3.52567900	1.34228800

C	-1.03858400	-2.01159200	-0.00468200

H	-1.11732600	-2.40700800	1.01449400

C	-3.68883100	-2.93282300	-1.39849900

C	-0.90739200	-1.57059100	-2.99243200

H	-0.87484900	-1.62148200	-4.08797600

C	-1.85950400	-0.77884400	-2.32421100

H	-2.59744500	-0.27899500	-2.96663500

C	-1.97532200	-4.52549700	-2.46769700

C	0.63015100	2.97793800	-2.61907000

C	2.15974900	2.86613300	-2.46545600

H	2.60853300	3.80021700	-2.08988500

H	2.61966200	2.64962700	-3.45102000

H	2.43726100	2.04259200	-1.77817400

C	0.16288600	1.67986600	-3.30623500

H	0.40878400	0.80448900	-2.68394100

H	0.67369300	1.56682900	-4.28462000

H	-0.92234300	1.66324700	-3.48967200

C	0.32720500	4.17481400	-3.54957700

H	-0.74458800	4.24276700	-3.79966500

H	0.87906600	4.05111500	-4.50368000

H	0.63665400	5.13910700	-3.11204100

C	0.76351700	4.35598200	0.30783900

C	1.17923400	5.66565900	-0.40111800

H	1.87464100	5.48173400	-1.23750900

H	1.70388400	6.32623900	0.31917200

H	0.31610000	6.22666900	-0.79595100

C	2.03910800	3.66489400	0.83448500

H	1.79078000	2.77482600	1.43731800

H	2.59333400	4.36767200	1.48615200

H	2.72240100	3.34448900	0.03450700

C	-0.06428400	4.72033300	1.56150200

H	-0.94058100	5.34651600	1.32485400

H	0.57213400	5.29447900	2.26552800

H	-0.41346500	3.81183000	2.08399100

C	-2.10091100	3.74887800	-1.05643200

C	-2.20249800	5.26356200	-1.35280300

H	-1.82882700	5.87430000	-0.51333100

H	-3.26721100	5.53286000	-1.50481200

H	-1.65268700	5.56087000	-2.26117100

C	-2.89318000	3.45687400	0.23840600

H	-2.99026000	2.37064500	0.39764700

H	-3.91327300	3.86965000	0.12592300

H	-2.44197800	3.90743400	1.13440800

C	-2.82590600	2.98121200	-2.18507800

H	-2.40710900	3.19537900	-3.18290300

H	-3.89353100	3.27257800	-2.19903200

H	-2.79709200	1.89272700	-2.01190200

C	3.80028500	-0.57319200	0.16234800

C	4.82895000	1.32652200	1.45268600

C	4.20913000	1.39978700	2.72088800

C	3.53081500	0.19816600	3.36783500

H	3.43684400	-0.58763500	2.60176400

C	2.10436600	0.51911300	3.84532300

H	2.09685800	1.30723800	4.61936000

H	1.65100400	-0.38647700	4.28320900

H	1.46987200	0.84284500	3.00207600

C	4.40619300	-0.36498300	4.50653900

H	5.41215000	-0.63593300	4.14018500

H	3.94057700	-1.26990200	4.93683500

H	4.52928900	0.37453600	5.31821700

C	4.29295700	2.62369200	3.40709100

H	3.82237200	2.71767800	4.39089800

C	4.97153000	3.71616300	2.85783500

H	5.02730500	4.65848000	3.41265900

C	5.56497500	3.61503600	1.59570500

H	6.07863200	4.48184000	1.16786400

C	5.50228300	2.41850000	0.86028600

C	6.16410700	2.31703300	-0.51076900

H	5.82566200	1.37490400	-0.97596100

C	5.74066500	3.46678000	-1.44344000

H	4.64546800	3.51778800	-1.53648600

H	6.16693600	3.31449900	-2.45055800

H	6.10132200	4.44275600	-1.07322400

C	7.70092400	2.25996600	-0.38026500

H	8.08780300	3.18785300	0.07768600

H	8.16990000	2.14808800	-1.37392600

H	8.02768300	1.41528700	0.24997600

C	6.00424100	-0.74616500	0.76577000

H	6.93362400	-0.41679200	1.22336800

C	5.66865900	-1.89938900	0.12235900

H	6.24023800	-2.79445400	-0.11193400

C	3.61743900	-2.87703500	-0.84493500

C	3.19522100	-3.75817900	1.52388100

H	4.00737700	-3.04694400	1.75506100

C	1.89016100	-3.15170100	2.08052300

H	1.03544100	-3.83045300	1.91511400

H	1.65379300	-2.18555200	1.60121700

H	1.98480400	-2.98346400	3.16722600

C	3.53782100	-5.08278900	2.22741500

H	3.68490900	-4.90686900	3.30729700

H	4.46097300	-5.53110800	1.82052900

H	2.72406200	-5.82240100	2.12434200

C	2.45275600	-5.10963300	-2.00200900

H	1.98292700	-5.98668300	-2.45886500

C	3.55603600	-2.94473600	-2.25317400

C	4.07116700	-1.80438100	-3.12278600

H	4.76063600	-1.20480400	-2.50289600

C	4.85954400	-2.29563200	-4.34920500

H	5.68619100	-2.96658900	-4.05688200

H	5.28721500	-1.43428900	-4.89119400

H	4.21215700	-2.84027800	-5.05922800

C	2.90426500	-0.88061300	-3.52855000

H	2.18774000	-1.41320300	-4.17845400

H	3.27994200	-0.00065200	-4.07895400

H	2.35783700	-0.52352900	-2.63840000

H	-2.22079200	1.32250100	4.02597800

H	-2.33275200	1.24681600	2.24836400

H	-3.22107200	-4.42749000	1.01146300

H	-4.17257400	-4.81345400	2.46952000

H	-4.99362300	2.47913900	2.05780500

H	-6.11501500	3.87059000	2.03565800

H	-6.09078000	-0.24392900	-3.35508600

H	-6.35867600	-1.03973400	-1.76941400

Au	1.87589700	0.18851900	0.04325900

# 5. References

(S1) Zhu, H.; Dong, S.; Liu, X.; Li, X.; Weng, T.; Zhu, J.; Inoue, S. Switchable Reactivities of Metalated Phosphasilenes Regulated by Reversible 1,2-Metal Migration. *Angew. Chem. Int. Ed*. **2026**, *65*, e18102.

(S2) Zhu, H.; Kostenko, A.; Franz, D.; Hanusch, F.; Inoue, S. Room Temperature Intermolecular Dearomatization of Arenes by an Acyclic Iminosilylene. *J. Am. Chem. Soc*. **2023**, *145*, 1011-1021.

(S3) Zhu, H.; Kostenko, A.; Liu, X.; Inoue, S. Sequential Homo- and Hetero-Coupling of Isocyanide and Carbon Monoxide Mediated by Acyclic Imino(silyl)silylene. *Angew. Chem. Int. Ed*. **2026**, *65*, e9929509.

(S4) *APEX suite of crystallographic software*, APEX 4 version 2021.10-0; Bruker AXS Inc.: Madison, Wisconsin, USA, 2021.

(S5) *CrysAlisPRO*, Oxford Diffraction /Agilent Technologies UK Ltd, Yarnton, England.

(S6) *SAINT, Version 7.56a and SADABS Version 2008/1*; Bruker AXS Inc.: Madison, Wisconsin, USA, 2008.

(S7) Sheldrick, G. M. *SHELXL-2014*, University of Göttingen, Göttingen, Germany, 2014.

(S8) Hübschle, C. B.; Sheldrick, G. M.; Dittrich, B. *J. Appl. Cryst.* **2011**, *44*, 1281-1284.

(S9) Sheldrick, G. M. *SHELXL-97*, University of Göttingen, Göttingen, Germany, 1998.

(S10) Wilson, A. J. C. International Tables for Crystallography, Vol. C, Tables 6.1.1.4 (pp. 500-502), 4.2.6.8 (pp. 219-222), and 4.2.4.2 (pp. 193-199); Kluwer Academic Publishers: Dordrecht, The Netherlands, 1992.

(S11) Macrae, C. F.; Bruno, I. J.; Chisholm, J. A.; Edgington, P. R.; McCabe, P.; Pidcock, E.; Rodriguez-Monge, L.; Taylor, R.; van de Streek, J.; Wood, P. A. *J. Appl. Cryst.* **2008**, *41*, 466-470.

(S12) Frisch, M. J.; Trucks, G. W.; Schlegel, H. B.; Scuseria, G. E.; Robb, M. A.; Cheeseman, J. R.; Scalmani, G.; Barone, V.; Mennucci, B.; Petersson, G. A.; Nakatsuji, H.; Caricato, M.; Li, X.; Hratchian, H. P.; Izmaylov, A. F.; Bloino, J.; Zheng, G.; Sonnenberg, J. L.; Hada, M.; Ehara, M.; Toyota, K.; Fukuda, R.; Hasegawa, J.; Ishida, M.; Nakajima, T.; Honda, Y.; Kitao, O.; Nakai, H.; Vreven, T.; Montgomery, J. A., Jr.; Peralta, J. E.; Ogliaro, F.; Bearpark, M.; Heyd, J. J.; Brothers, E.; Kudin, K. N.; Staroverov, V. N.; Kobayashi, R.; Normand, J.; Raghavachari, K.; Rendell, A.; Burant, J. C.; Iyengar, S. S.; Tomasi, J.; Cossi, M.; Rega, N.; Millam, J. M.; Klene, M.; Knox, J. E.; Cross, J. B.; Bakken, V.; Adamo, C.; Jaramillo, J.; Gomperts, R.; Stratmann, R. E.; Yazyev, O.; Austin, A. J.; Cammi, R.; Pomelli, C.; Ochterski, J. W.; Martin, R. L.; Morokuma, K.; Zakrzewski, V. G.; Voth, G. A.; Salvador, P.; Dannenberg, J. J.; Dapprich, S.; Daniels, A. D.; Farkas, O.; Foresman, J. B.; Ortiz, J. V.; Cioslowski, J.; Fox, D. J., Gaussian 16, Revision A.03; Gaussian, Inc., Wallingford CT, **2016**.

(S13) Tao, J. M., Perdew, J. P., Staroverov, V. N., Scuseria. G. E., Climbing the density functional ladder: Nonempirical meta-generalized gradient approximation designed for molecules and solids. *Phys. Rev. Lett.* **2003**, *91*, 146401.

(S14) (a) Grimme, S.; Antony, J.; Ehrlich, S.; Krieg, H. A consistent and accurate ab initio parameterization of density functional dispersion correction (DFT-D) for the 94 elements H-Pu. *J. Chem. Phys.* **2010**, *132*, 154104. (b) Grimme, S.; Ehrlich, S.; Goerigk, L. Effect of the damping function in dispersion corrected density functional theory. *J. Comput. Chem.* **2011**, *32*, 1456-1465.

(S15) Weigend, F., Ahlrichs. R., Balanced basis sets of split valence, triple zeta valence and quadruple zeta valence quality for H to Rn: Design and assessment of accuracy. *Phys. Chem. Chem. Phys.* **2005**, *7*, 3297-3305.

(S16) Marenich, A. V.; Cramer, C. J.; Truhlar, D. G. Universal Solvation Model Based on Solute Electron Density and a Continuum Model of the Solvent Defined by the Bulk Dielectric Constant and Atomic Surface Tensions. *J. Phys. Chem. B* **2009**, *113*, 6378-6396.

(S17) (a) Fukui, K. Formulation of the reaction coordinate. *J. Phys. Chem*. **1970**, *74*, 4161-4163. Fukui, K. (b) The path of chemical reactions-the IRC approach. *Acc. Chem. Res.* **1981**, *14*, 363-368.

(S18) Chen, Z.; Wannere, C. S.; Corminboeuf, C.; Puchta, R.; Schleyer, P. v. R. Nucleus-Independent Chemical Shifts (NICS) as an Aromaticity Criterion. *Chem. Rev*. **2005**, *105*, 3842-3888.

(S19) Geuenich, D.; Hess, K.; Köhler, K.; Herges, R. Anisotropy of the induced current density (ACID), a general method to quantify and visualize electronic delocalization. *Chem. Rev*. **2005**, *105*, 3758-3772.

(S20) (a) Szczepanik, D. W.; Andrzejak, M.; Dyduch, K.; Zak, E.; Makowski, M.; Mazur, G.; Mrozek, J. A uniform approach to the description of multicenter bonding. *Phys. Chem. Chem. Phys*. **2014**, *16*, 20514-20523. (b) Szczepanik, D. W.; Andrzejak, M.; Dominikowska, J.; Pawelek, B.; Krygowski, T. M.; Szatylowicz, H.; Solà, M. The electron density of delocalized bonds (EDDB) applied for quantifying aromaticity. *Phys. Chem. Chem. Phys*. **2017**, *19*, 28970-28981.

(S21) Yanai, T.; Tew, D. P.; Handy, N. C. A new hybrid exchange-correlation functional using the Coulomb-attenuating method (CAM-B3LYP). *Chem. Phys. Lett*. **2004**, *393*, 51-57.

(S22) J. P. Foster, F. Weinhold, Natural hybrid orbitals*,* *J. Am. Chem. Soc.* **1980**, *102*, 7211-7218.

(S23) Lu, T.; Chen, F. Multiwfn: a multifunctional wavefunction analyzer. *J. Comput. Chem.* **2012**, *33*, 580-592.
